# First-in-human Phase I studies of PRS-080#22, a hepcidin antagonist, in healthy volunteers and patients with chronic kidney disease undergoing hemodialysis

**DOI:** 10.1371/journal.pone.0212023

**Published:** 2019-03-27

**Authors:** Lutz Renders, Klemens Budde, Christian Rosenberger, Rachel van Swelm, Dorine Swinkels, Frank Dellanna, Werner Feuerer, Ming Wen, Christiane Erley, Birgit Bader, Claudia Sommerer, Matthias Schaier, Karoline Meurer, Louis Matis

**Affiliations:** 1 Nephrologie, Klinikum Rechts der Isar, Munich, Germany; 2 Nephrologie und Internistische Intensivmedizin, Charité Universitätsmedizin, Berlin, Germany; 3 Department of Laboratory Medicine, Translational Metabolic Laboratory, Radboud University Medical Center, Nijmegen, The Netherlands; 4 Hepcidinanalysis.com, Nijmegen, The Netherlands; 5 MVZ DaVita, Düsseldorf, Germany; 6 Nuvisan GmbH, Neu-Ulm, Germany, retired; 7 Medizinische Klinik II, St. Joseph Krankenhaus Berlin-Tempelhof, Germany; 8 Nierenzentrum, Heidelberg, Germany; 9 Pieris Pharmaceuticals, GmbH, Freising, Germany; 10 Pieris Pharmaceuticals, Inc., Boston, Massachusetts, United States of America; Medizinische Universitat Graz, AUSTRIA

## Abstract

In chronic kidney disease both renal insufficiency and chronic inflammation trigger elevated hepcidin levels, which impairs iron uptake, availability. and erythropoiesis. Here we report the two first-in-human phase 1 trials of PRS-080#22, a novel, rationally engineered Anticalin protein that targets and antagonizes hepcidin. A single intravenous infusion of placebo or PRS-080#22 was administered to 48 healthy volunteers (phase 1a) and 24 patients with end stage chronic kidney disease (CKD) on hemodialysis (phase 1b) at different doses (0.08-16mg/kg for the phase 1a study and 2-8mg/kg for the phase 1b study) in successive dosing cohorts. The primary endpoint for both randomized, double-blind, phase 1 trials was safety and tolerability. Following treatment, all subjects were evaluable, with none experiencing dose limiting toxicities. Most adverse events were mild. One serious adverse event occurred in the phase 1b (CKD patient) study. There were no clinically significant changes in safety laboratory values or vital signs. PRS-080#22 showed dose-proportional pharmacokinetics (PK), with a terminal half-life of approximately three days in healthy volunteers and 10 to 12 days in CKD patients. Serum hepcidin levels were suppressed in a dose dependent manner and remained low for up to 48 hours after dosing. PRS-080#22 dose-dependently mobilized serum iron with increases in both serum iron concentration and transferrin saturation. No consistent changes were observed with regard to ferritin, reticulocytes, hemoglobin, and reticulocyte hemoglobin. Low titer anti-drug-antibodies were detected in five healthy volunteers but in none of the CKD patients. PRS-080#22, a novel Anticalin protein with picomolar affinity for hepcidin, was safe and well-tolerated when administered to healthy volunteers and CKD patients at all doses tested. The drug exhibited linear pharmacokinetics, longer half-life in CKD patients in comparison to healthy volunteers as well as expected pharmacodynamic effects which hold promise for further clinical studies.

## Introduction

Anemia is a frequent complication of chronic kidney disease (CKD). The incidence and prevalence of anemia increases in patients with more advanced stages of CKD, as kidney function declines [1, 2]. The most common causes are iron deficiency and insufficient erythropoietin (EPO) production [2]. Consequently, current treatment regimens consist of iron (oral or intravenous (IV)), erythropoietin stimulating agents (ESAs), or a combination of both. Adequate iron stores are essential for achieving maximum benefit from ESAs; therefore, most end-stage CKD patients also receive IV iron supplementation. However, IV iron may cause infrequent but severe adverse reactions and concerns about long-term safety have been raised [3,4]. A clear relationship between iron therapy and hepatic iron overload has been demonstrated by hepatic MRI [5]. ESA therapy, particularly at high doses, has been associated with an increased risk of cardiovascular complications and mortality [6]. Finally, a significant number of patients remain anemic despite combination therapy with ESAs and IV iron. In this light, there is a significant need for novel therapeutic approaches to address ESA/iron resistant anemia in CKD patients.

Hepcidin, a liver-derived 25 amino acid peptide hormone, is a central regulator of iron homeostasis. Many disorders of iron imbalance can be attributed to aberrant hepcidin production [7]. Hepcidin binds to and degrades the iron export channel ferroportin in both the gut and the plasma membranes of reticuloendothelial cells, thereby inhibiting iron transport. Elevated plasma levels of hepcidin cause iron sequestration in macrophages, which may lead to functional iron deficiency despite replete iron stores [8]. Hepcidin is frequently elevated in CKD patients [9–11] and is thought to represent a root cause of the hypoferremia, iron-restricted erythropoiesis, and refractory anemia in these patients. As hepcidin levels correlate directly with creatinine levels and inversely with glomerular filtration rate (eGFR), elevated hepcidin in CKD patients may in part be due to decreased renal clearance [12]. Chronic inflammation may also contribute to increased levels of hepcidin in CKD [13–15].

In this light neutralizing hepcidin activity could represent a promising approach for the treatment of functional iron deficiency anemia in CKD patients.

PRS080#022 is a PEGylated (polyethylene glycol bound) Anticalin protein that antagonizes hepcidin with picomolar affinity. Anticalin proteins are a novel class of small, highly stable proteins with designed ligand-binding properties derived from the natural human lipocalin scaffold [16]. Lipocalins are a widespread family of low molecular weight binding proteins that transport, store, or sequester small biological compounds like vitamins and hormones in many organisms, including humans [17].

PRS-080#022 showed a benign toxicity profile in preclinical studies, including in non-human-primates; the results of the toxicity studies did not point to a target organ of toxicity. In preclinical studies the elimination route was mainly renal.

Further, repeated PRS-080#022 dosing in cynomolgus monkeys potently suppressed hepcidin resulting in sustained iron mobilization [18]. In this light, hepcidin inhibition by PRS-080#022 has the potential to ameliorate functional iron deficiency anemia in CKD patients. Here we are reporting the results of the first two (phase 1a and 1b) clinical trials of PRS-080#022 in humans.

## Methods

**Fig 1:**
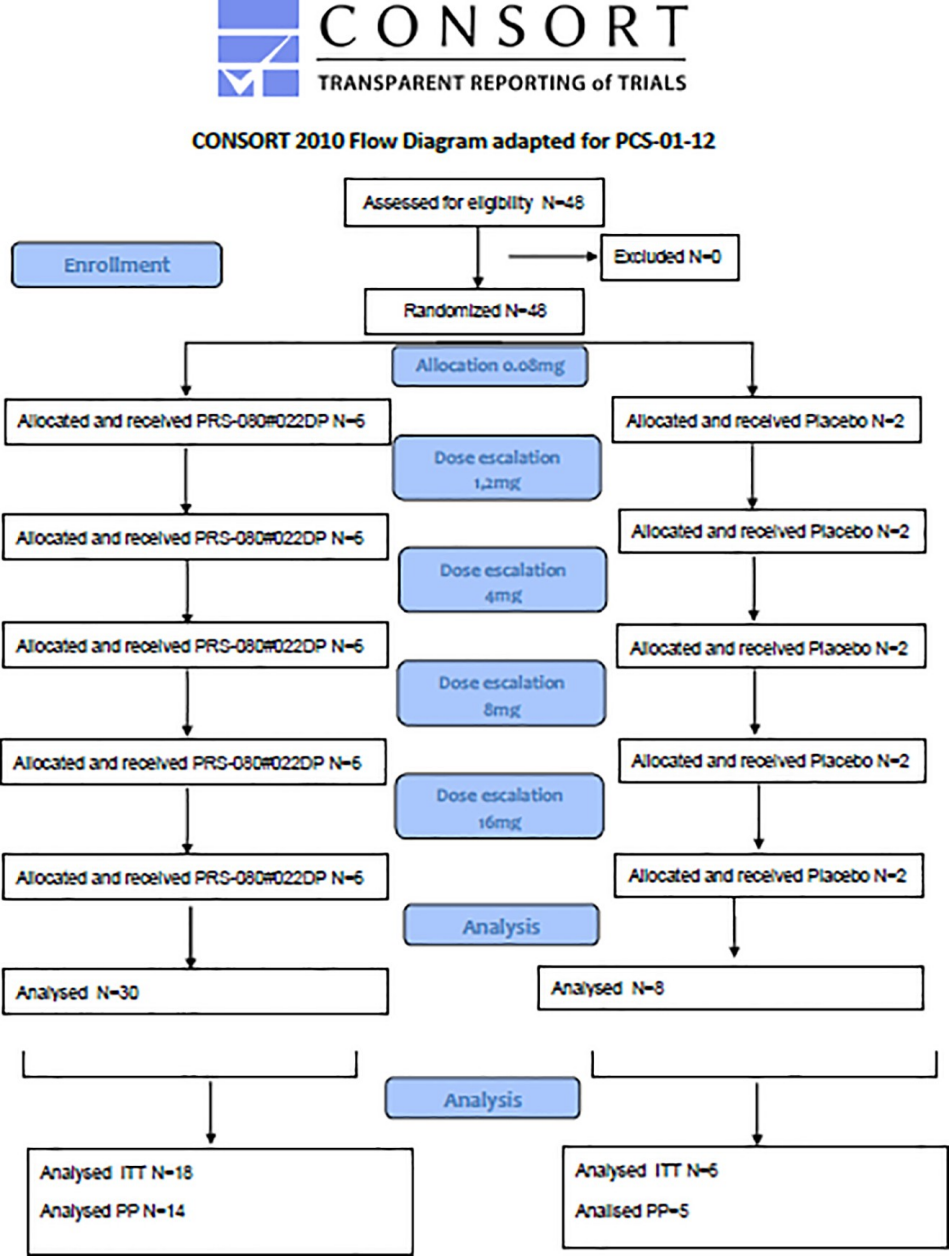
CONSORT flowchart for study PCS-01-12 in healthy volunteers.

**Fig 2:**
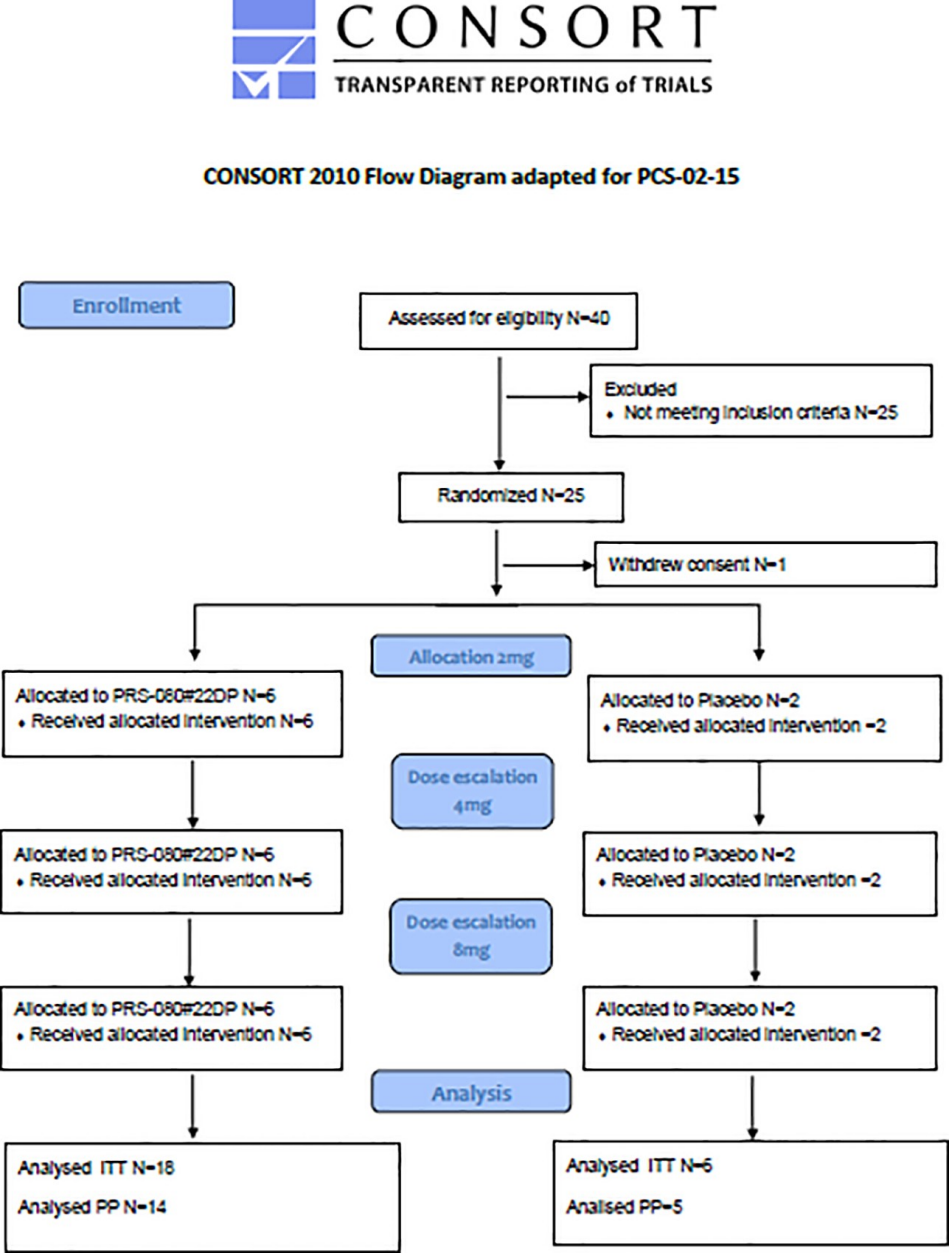
CONSORT flowchart for study PCS-02-15 in CKD patients.

The phase 1a study in healthy volunteers was conducted by the phase 1 unit of Nuvisan Pharma Services GmbH in Neu-Ulm/Germany and reviewed and approved by the Ethical Committee (EC) of the Bavarian State Medical Council, Muehlbaurstrasse 16, 81677 Munich (approval 03 Nov 2014), Germany; the multi-center phase 1b CKD study was performed in 5 clinical centers in Germany and approved by applicable regional independent ethics committee (IEC) according to German regulations (approval 07 Mar 2016). Both studies were supervised by the German Federal Ministry for Drugs and Medical Products (BfArM). Originally the first study was planned in two stages, the first one to be conducted as single administration, the second as multiple administrations. After consultations with BfArM the second stage was cancelled and is currently being performed in CKD patients as separate study. The first part was conducted as planned and is presented in this manuscript. All subjects gave their signed, informed consent to participate in the study. Study periods (first subject in–last follow up visit) were for the healthy volunteer study: December 04, 2014 –June, 8 2015 and for the CKD study: June 07, 2016 –March 17, 2017. The protocols for these trials are available as supporting information; S1 and S2 Protocol. Consort flowcharts for both studies are provided as Figs 1 and 2. Both single ascending dose studies were randomized, double-blind and placebo controlled. Both studies are registered on Clintrials.gov (https://clinicaltrials.gov/, NCT02340572 and NCT02754167). As there is no regulatory obligation to publish early phase 1 studies, this was not considered a priority at the time of the start of the first trial. Registration was done after recruitment of volunteers had started. The authors however confirm that all related and following trials have been appropriately registered.

### Subjects

For the healthy volunteer study, common inclusion criteria for healthy volunteers were applied. Additionally, ferritin levels had to be between 10 and 300ng/mL. Iron treatment or blood transfusions within 90 days prior to treatment, as well as EPO treatment within 1 year, were prohibited. All screened volunteers met the inclusion and exclusion criteria and were included in the study.

The main inclusion criteria for the CKD study were: Stage 5 CKD having been on hemodialysis for at least 90 days, stable (less than 30% change) ESA dose for 6 weeks prior to study medication administration, hemoglobin (Hb) 9.0–12.0 g/dL with no changes greater than 1.5 g/dL over the last 6 weeks prior to study medication administration, ferritin ≥300 ng/mL, transferrin saturation (TSAT) ≤40%, hepcidin 5–75nM. Any iron medication had to be stopped 7 days before the administration of study medication. Forty patients were screened, of which 24 were included and treated. Fifteen patients did not meet the inclusion criteria and one patient withdrew informed consent before treatment.

### Trial design and treatment

PRS080#022 was dissolved in 0.9% NaCl solution by an independent pharmacy and administered intravenously by slow infusion. For placebo only 0.9% NaCl was provided in identical vials, labelled in a blinded fashion. In the healthy volunteers study a volume of 250 mL was given over 2 hours. Six dose groups (0.08, 0.4, 1.2, 4, 8, 16 mg/kg) were assessed in the healthy volunteer study. Six subjects received active drug and 2 subjects received placebo in each dose group. Dosing was performed stepwise in sub-groups of 2 subjects each for dose group 1 and for the following dose groups in one subgroup of 2 and three sub-groups of 3 subjects each. Drug administration started sequentially between each individual subject with an interval of 150 minutes after start of infusion. Safety data were reviewed after each step of the staggered dosing and before dose escalation. The volunteers were closely monitored (confined to the phase 1 unit for 72 hours), follow up evaluations were performed at 120 hours, 240 hours and 28 days (± 2).

In the CKD study, a volume of 100 mL was given over 1 hour and 3 dose groups (2, 4, 8 mg/kg) were included. Six subjects received active drug and 2 subjects received placebo in each dose group. Patients were observed for at least 4 hours after the end of infusion. Follow-up visits were performed to assess safety, PK and PD at days 1, 2, 3, 5, 7, 14, 21, and 28 after treatment. The decision to escalate dose (by the dose escalation committee, DEC) was based on a review of safety and pharmacodynamic data (PD) after the last patient in a cohort had completed day 7.

### Safety assessments

Safety assessments were performed on admission to the clinical unit for the HV study and within the 4 week screening period for the CKD patient study, before dosing, and at scheduled intervals after dosing. These assessments included: monitoring for adverse events (as specified by the study protocols and according to GCP guidelines), vital signs including blood pressure, pulse rate, respiratory rate and body temperature, 12-lead ECG, local tolerability at injection site, and clinical safety laboratory tests including biochemistry, hematology, coagulation, and urinalysis (only the healthy volunteer study) parameters. In addition, blood samples in the healthy volunteer study were immediately frozen and processed for inflammatory cytokines (Il-1β, IL-6, IFN-γ, and TNF-α) by a validated multiplex assay (Meso Scale Discovery) before dosing and at scheduled intervals after dosing.

### Pharmacokinetic assessments

Venous blood samples were collected for total and free PRS-080#022 assays. Blood samples were collected before (*t = 0*) and at fixed intervals for up to 240 hours after single intravenous dosing. Concentrations of free and total PRS-080#022 in plasma were assessed using validated enzyme linked immunosorbent assay and electro-chemi-luminescence methods, respectively. The free PRS-080#022 assay employed capturing via hepcidin and detection via a PRS-080#022 specific antibody; the total PRS-080#22 assay employed 2 different PRS-080#022 antibodies for capturing and detection, independent of bound hepcidin. The lower limit of detection was 0.1ng/mL.

PK parameters were derived by non-compartmental methods [19] using programs developed in SAS version 9.3.

Parameters comprised maximal concentration (C_max_), time of C_max_ (t_max_), area under the concentration time curve (AUC) from time 0 to last sample with a quantifiable concentration (AUC_0-t_), AUC from 0 to infinity (AUC_0-inf_), terminal half-life (t_1/2_), apparent mean residence time (MRT), total plasma clearance (CL), volume of distribution during the terminal phase (V_z_) and apparent volume of distribution at steady state (V_ss_).

Based on the single dose pharmacokinetic behavior in CKD patients, simulations of steady-state conditions using a– 2 compartment model, were performed to assess the risk of drug accumulation in future studies under repeated dosing conditions.

### Pharmacodynamic assessments

Free plasma hepcidin concentrations were measured by weak cation exchange enrichment of hepcidin followed by matrix-assisted laser desorption/ionization time-of-flight mass spectrometry. [20,21] This assay specifically detects free hepcidin which is not bound to PRS-080#022. Samples were taken at the same time points as the PK samples plus additional measurements at admission and 28 days after dosing.

The following pharmacodynamic variables were also measured before and up to 28 days after dosing by common methods: iron, transferrin saturation and ferritin, and Hb, reticulocytes and reticulocyte Hb. Blood sampling was done at the same time of the day (before/after dialysis) except the early timepoints during day 1.

### Immunogenicity assessments

Blood samples for the determination of serum concentrations of anti-drug antibodies (ADA) were taken pre-dose and 28 days after dosing. ADAs were determined by using a bridging ligand binding assay, which was developed for PRS-080#022 DP and validated [22–24]. The sensitivity of the test was 12.5 ng/mL. Samples were initially screened at a dilution of 1:100. Then a confirmative assay was performed and positive samples were further analyzed in a titration assay.

### Statistical methods

As the studies were exploratory in nature, no hypotheses were formulated and no number calculations were performed. All parameters were summarized descriptively by dose level. Patients were included in consecutive order by the investigators, randomly allocated and assigned to treatment groups with an interactive voice response system (IVRS). All statistical analyses were done using SAS version 9.3 (SAS Institute Inc, Cary, NC, USA) on a Windows XP personal computer.

The PK parameters AUC_0-t_, AUC_0-inf_ and C_max_ of total PRS-080#022 were assessed for dose-proportionality using the respective linear regression model relating the logarithm of dose as independent variable to the logarithm of the PK parameters. In order to investigate dose-proportionality, an estimate for the slope of the regression line with a 95% confidence interval was presented. A value which was appreciably different from 1 provided evidence for non-proportionality.

## Results

Differences with regard to age and weight between the dosing groups in both studies were small and without clinical relevance. CKD patients had to be on hemodialysis for at least 90 days before entering the phase 1b study. Blood transfusions within 2 months of screening were prohibited. They all followed a dialysis regimen of 3 times a week; different dialysis protocols were allowed (e.g., overnight dialysis).

The disposition of subjects is provided in Table 1, additional descriptive statistics are provided in S1 and S2 Tables.

**Table 1:** Disposition of subjects and selected baseline laboratory parameters in healthy volunteers and CKD patients, mean values and [ranges].

	Mean BMI	Mean Age (years)	Mean Weight(kgs)	Gender	Creatinine mg/dl	Albumine g/dl	Iron μg/dl	Ferritin ng/mL	eGFR* mL/min
Healthy volunteers (N = 48)	24.5 [20.4–28.8]	36.1 [18–50]	76.8 [60.1–90.0]	100% male	0.9 [0.7–1.2]	4.7 [4.2–5.5]	109.7 [39.1–252.42]	74.2 [14.3–225.4]	123.1
CKD (N = 18)	26.6 [18.3–35.5]	55.4 [28–72]	77.1 [50–107.4]	71% male, 29% female	8.6 [4.2–14.5]	4.2 [3.6–4.9]	85.5 [29–106]	732.7 [310–1603]	10.2

*calculated using the Cockroft & Gault Formula based on mean values, therefore a range is not meaningful

### Safety

PRS-080#022 was well tolerated in both studies. There were no serious adverse events (SAEs) observed in the healthy volunteer study. One SAE was reported in the CKD study. This patient suffered from worsening of dry gangrene at one of the toes. The illness of diabetic foot syndrome was known for 10 years and the patient had previously undergone amputation of a toe due to this condition. Prior to the study’s initiation the patient had suffered from episodic deterioration, such that a progression of the disease could be expected. The investigator assessed this event as not related to study medication but to the underlying disease. However, the German competent authorities requested to relabel the AE as possibly related as a causal relationship could not be ruled out.

Apart from infusion-related reactions like flushing and injection site erythema (2 cases in the healthy volunteers’ study) the related AE’s were unspecific in nature (headache, abdominal discomfort, decreased exercise tolerance) and did not occur more than once per dose group, apart from headache (2 cases in the 16mg dose group) (Tables 2 and 3).

**Table 2:** Adverse events (frequency) in healthy volunteers.

-	Placebo N = 12	0.08mg/kg N = 6	0.4 mg/kg N = 6	1.2 mg/kg N = 6	4 mg/kg N = 6	8 mg/kg N = 6	16 mg/kg N = 6
Subjects with any AE	5 (41.7%)	1 (16.7%)	3 (50%)	3 (50%)	3 (50%)	3 (50%)	4 (66%)
Number of AEs	7	1	6	7	5	8	5
Mild AE	6 (86%)	0	5 (83%)	5 (71%)	5 (100%)	5 (63%)	4 (80%)
Moderate AE	1 (14%)	1 (100%)	1 (17%)	2 (29%)	0	3 (38%)	1 (20%)
ADR*	1 (14%)	0	0	0	2 (40%)	2 (25%)	2 (40%)
Kind of ADR	Headache	na	na	na	Headache, Injection site erythema	Abdominal discomfort, Flushing	Headache

*related or possibly related ADR = adverse drug reaction; na = not applicable

**Table 3:** Adverse events (frequency) in patients with CKD.

-	Placebo, N = 6	2 mg/kg, N = 6	4 mg/kg, N = 6	8 mg/kg, N = 6
Subjects with any AE	2 (33.3%)	5 (83.3%)	4 (66.7%)	1 (16.7%)
Number of AEs AE	3	8	10	1
Mild AE	3 (100%)	7 (88%)	10 (100%)	0
Moderate AE	0	1 (13%)	0	1 (100%)
ADR*	0	1 (13%)	2 (20%)	0
Kind of ADR		Decreased exercise tolerance Worsening of dry gangrene	Abdominal discomfort, Headache	
SAE**	na	1	na	na

*related or possibly related

** The ADR “worsening of dry gangrene” was reported as SAE

na = not applicable

In both studies, individual data related to biochemistry, hematology, coagulation and urinalysis parameters showed no clinically relevant changes after administration of PRS-080#022. There were no effects on vital signs (e.g., heart rate, body temperature, blood pressure,) and ECG in comparison to placebo in any of the patients.

There were no hypersensitivity responses and no infusion reactions in either study, nor were there any changes in inflammatory cytokine levels in healthy volunteers.

Local tolerability was excellent: One mild injection site erythema after administration of 4 mg/kg in the HV study was the only local finding (reported as AE, see Table 2).

### Pharmacokinetic evaluations

The main PK parameters (mean values and SD) are summarized in Table 4, additional descriptive statistics are provided in S3 and S4 Tables. Time concentration curves for the arithmetic mean of PRS-080#022 are presented in S1 and S2 Figs.

**Table 4:** Pharmacokinetic parameters in healthy volunteers and CKD patients, mean values and (SD, ranges for t_max_).

	CKD Study			Healthy volunteer Study			
Doses	2 mg/kg	4 mg/kg	8 mg/kg	1.2 mg/kg	4 mg/kg	8 mg/kg	16 mg/kg
Free PRS-080#022							
AUC _0-t_ [h*μg/mL]	484 (39.7)	1713 (48.4)	4 242 (33.0)	748 (68.7)	4 460 (55.0)	10 216 (40.0)	18 497 (42.3)
AUC _0-∞_ [h*μg/mL]	466 (37.2)	1 765 (46.3)	4 558 (32.4)	718 (78.9)	4 581 (57.9)	10 185 (47.6)	22 051 (19.4)
C _max_ [μg/mL]	29.0 (25.0)	128.4 (103.3)	159.6 (26.0)	28.7 (20.8)	114.9 (24.7)	202.0 (18.6)	397.2 (21.2)
t _max_ [h]	1.0 (1.0–5.1)	1.0 (1.0–1.0)	1.0 (1.0–5.0)	3.0 (2.0–4.0)	3.0 (2.0–10.0)	2.0 (2.0–6.0)	2.0 (2.0–3.0)
t _1/2_ [h]	286.1 ^b^ (24.4)	251.6 (28.6)	218.1 ^b^ (37.0)	61.6 ^a^ (30.6)	44.6 (30.3)	46.5 ^a^ (51.5)	41.0 ^a^ (28.1)
V _ss_ [L/kg]	0.51 ^b^ (47.5)	0.16 (93.2)	0.11 ^b^(42.7)	0.08 ^a^ (39.7)	0.04 (30.0)	0.05 ^a^(21.5)	0.05 ^a^ (6.4)
Total PRS-080#022							
AUC _0-t_ [h*μg/mL]	6 912 (28.7)	16 205 (37.3)	33 265 (21.5)	2 259 (7.4)	7 460 (10.1)	14 899 (16.4)	25 253 (18.3)
AUC _0-∞_ [h*μg/mL]	8 268 (26.8)	18 651^a^ (45.7)	40 601 (23.2)	2 560 (9.9)	8 263 (11.7)	16 940 (16.5)	27 339 (17.9)
C _max_ [μg/mL]	43.1 (29.8)	211.6 (102.9)	233.3 (49.1)	33.7 (13.0)	119.0 (16.9)	240.9 (23.6)	364.1 (12.2)
t _max_ [h]	1.0 (1.0–20.3)	1.0 (1.0–5.0)	1.0 (1.0–20.8)	2.5 (2.0–4.0)	2.5 (2.0–10.0)	3.0 (3.0–10.0)	3.0 (2.0–4.0)
t _1/2_ [h]	259.1 (21.8)	237.4 ^a^ (15.8)	270.2 (21.6)	79.7 (13.1)	72.6 (12.7)	79.2 (11.4)	79.5 (15.2)
V _ss_ [L/kg]	0.09 (32.5)	0.07 ^a^ (60.6)	0.07 (25.5)	0.05 (8.4)	0.05 (11.8)	0.05 (18.1)	0.06 (22.4)

For the healthy volunteer study the two lowest dose groups (0.04 and 0.08) are not included. Geometric mean and coefficient of variation in percent are presented. except for t_max_ for which median and range are presented. N = 6 for each treatment group if not indicated otherwise.

^a^ N = 4.

^b^ N = 5.

AUC_0-t_ = area under the concentration time curve, time 0 to last quantifiable sample; AUC_0-∞_ = area under the concentration time curve, time 0 extrapolated to infinity; C_max_ = measured maximum concentration; N = number of patients; t_max_ = time of observed maximum concentration; t_1/2_ = terminal half-life, V_ss_ = volume of distribution at steady state.

In both studies AUC’s increased with the dose of study medication. The AUC’s for free PRS-080#022 were approximately 2-fold higher in the healthy volunteer study, whereas for total PRS-080#022 the AUC’s were higher in the CKD study. Free PRS-080#022 increased slightly more than dose-proportionally (especially in the lower dose groups), whereas total PRS-080#022 showed a dose proportional AUC increase. C_max_ increased with dose as well, with very similar results in both studies. Elimination half-life (t _½_) ranged from 41–62 hours for free PRS-080#022 and 73–80 hours for total PRS-080#022 in healthy volunteers. In CKD patients the elimination half-life was 5–6 times longer, between 218 and 286 hours (9–12 days). For total PRS-080#022 the volume of distribution did not differ between doses, or between healthy volunteers and end stage CKD patients, for free PRS-080#022volume of distribution was slightly higher for the CKD patients. However, the volume of distribution was very low in general suggesting no significantly different patterns of distribution between healthy volunteers and CKD5 patients.

The regression analysis for total PRS-080#022 to evaluate dose proportionality did not show a deviation from the proportionality assumption (the 95% confidence intervals slopes for AUC_0-t_, AUC_0-∞_, and C_max_ included the “1”-value.)

The 2-compartmental analysis mainly confirmed the results presented above. Additionally, the bi-exponential elimination of free PRS-080#022 in each dose group was analyzed, with a fast first disposal half-life [t_1/2(α)_] of 6.1 hours (2 mg/kg), 12.5 hours (4 mg/kg), and 16.5 hours (8 mg/kg). For total PRS-080#022-DP, the t_1/2(α)_ was 9.9 hours (2 mg/kg), 18.3 hours (4 mg/kg), and 13.9 hours (8 mg/kg). The increase in half-life and AUC of total and free PRS-080#022 was more pronounced between the 2 mg/kg dose group and both higher dose groups.

Steady-state simulations were performed in order to evaluate the risk for accumulation in case of multiple administration for future studies. They showed an accumulation factor of approximately 2.5 and indicate that steady-state of total and free PRS-080#022 will be reached after approximately 6 weekly infusions of 2, 4, or 8 mg/kg PRS-080#022-DP. The simulation curve for free PRS-080#022-DP is presented as S3 Fig.

In order to evaluate a potential influence of dialysis on the time concentration curves, an analysis between the 44 and 48 hour time points (dialysis window at day 2) was performed in the CKD study. No consistent changes were observed between 44 and 48 hours after administration in any of the dose groups.

### Fast response Pharmacodynamic Parameters (Iron, TSAT, Ferritin)

In the healthy volunteer study, the two lowest dose groups did not show any response in iron and TSAT, while in the 4 higher dose groups responses were dependent on the pre-dose hepcidin levels. Eight subjects did not show any response, all having hepcidin values below the lower limit of quantification (0,5 nM). Three subjects with hepcidin values below 1 nM showed a weak response, However, those subjects who had higher hepcidin values showed a response pattern similar to the CKD patients. The dependence of the iron/TSAT response on detectable hepcidin levels in the healthy volunteers indicated that the drug’s mechanism of action was target dependent.

**Fig 3:**
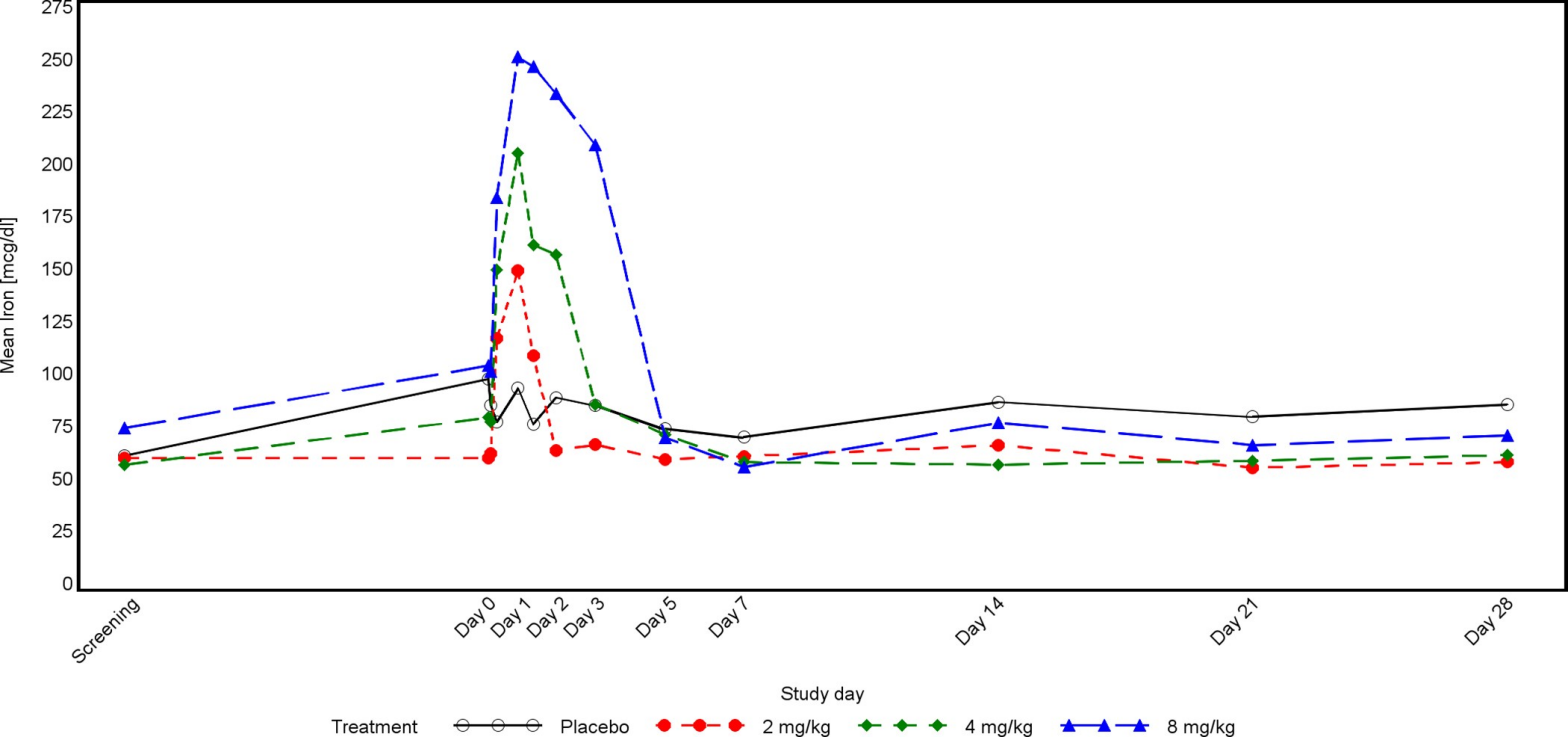
Time concentration curve for iron in CKD patients.

**Fig 4:**
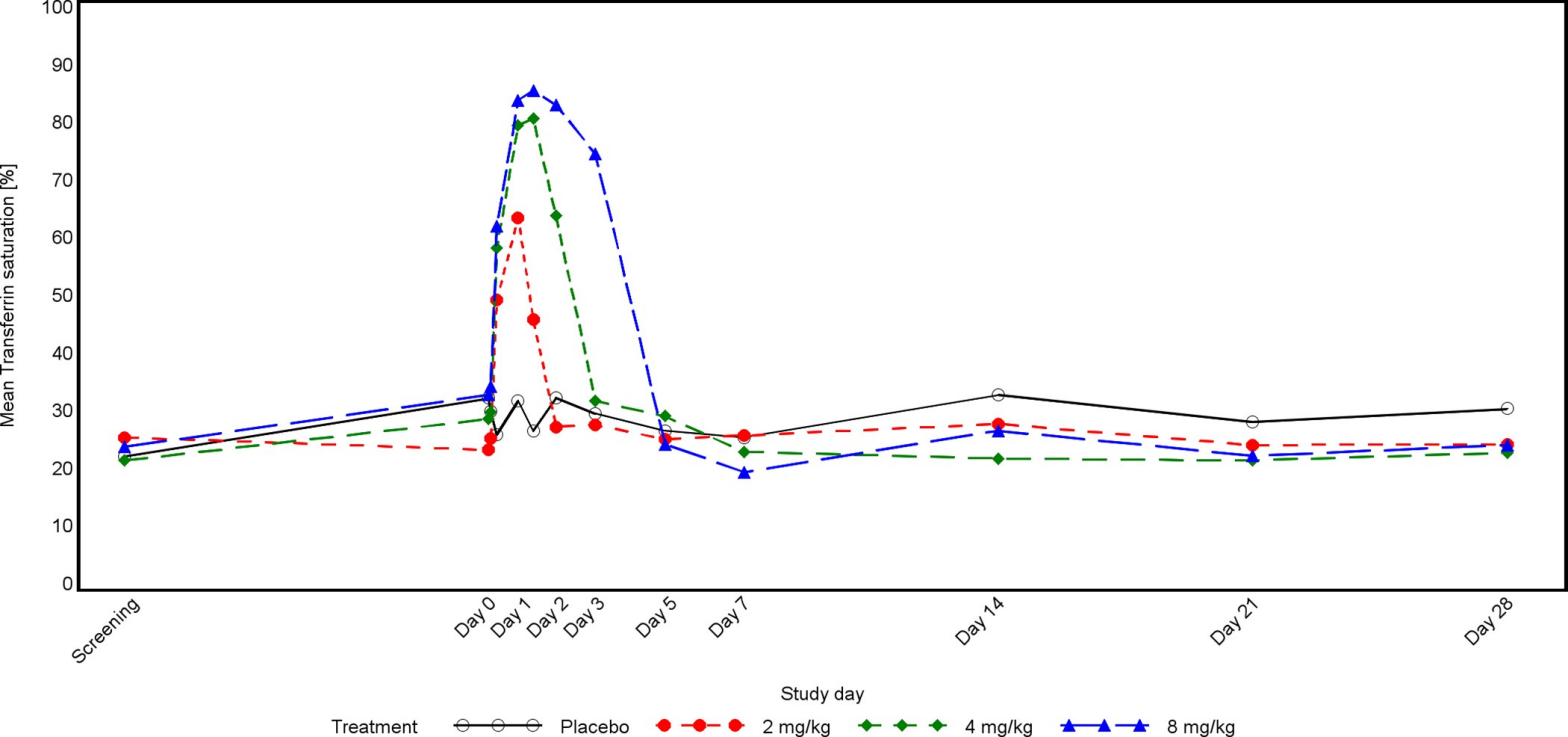
Time concentration curve for TSAT in CKD patients.

In the CKD study, patients’ serum iron concentrations were not balanced at baseline, they seemed to increase with dose, which is related to the small sample size. Serum iron and TSAT increased markedly after dosing with PRS-080#022, with peak values at 19 hours post-dose for the lower dose groups and at 29 hours in the 8mg dose group (Figs 3 and 4). The duration of the effect was also dose dependent and lasted until 72 hours post PRS-080 administration in the highest dose group. In this group a transient decrease below the mean baseline value was observed at Day 7 (Fig 1). Iron and TSAT values are reported in S5 Table.

There were no consistent dose-related post-dose changes for ferritin in either of the two studies (S6 Table)

### Hepcidin (free) analysis

Hepcidin plasma levels showed some variation between screening and day 1 (time 0) with a decline observed in the CKD patients. As expected, the CKD5 patients had higher baseline hepcidin values compared to the healthy volunteers. Following dosing with PRS-080-#022 in both studies, free hepcidin plasma concentrations decreased rapidly and remained suppressed in a dose-dependent fashion. A subsequent dose related elevation (rebound) of hepcidin levels above their initial baseline was then observed, persisting beyond the return of iron levels to baseline and then gradually declining also in a dose dependent fashion. (Table 5, S4 and S5 Figs, S7 and S8 Tables). The magnitude and duration of the hepcidin elevation were greater in the CKD patients than in the healthy volunteers (Table 5).

**Table 5:** Free hepcidin, mean values (SD) over time in healthy volunteers and CKD patients.

Hepcidin nM (SD)	CKD Study			Healthy volunteer Study			
	2mg/kg N = 6	4mg/kg N = 6	8mg/kg N = 6	1,2mg/kg N = 6	4mg/kg N = 6	8mg/kg N = 6	16mg/kg N = 6
Screening	18.1 (7.7)	22.1 (11.8)	30.7 (9.2)	1.9 (3.7)	1.7 (2.0)	0.8 (1.3)	2.6 (3.8)
0 h	7.4 (8.5)	14.4 17.3)	21.1 (4.5)	2.1 (2.3)	2.3 (2.2)	1.1 (1.4)	6.1 (12.1)
1 h	3.1 (6.2)	0.8 (0.9)	1.1 (0.7)	0.3 (0)	0.3 (0)	0.3 (0)	0.7 (1.0)
5/6 h*	9.3 (20.6)	1.6 2.3)	1.6 (0.6)	0.3 (0)	0.34 (0.3)	0.5 0.3)	1.1 (1.3)
18/19 h*	50.1 (31.6)	14.0 (18.5)	12.1 (3.1)	0.8 (0.7)	1.1 0.9)	1.1 (1.0)	2.6 (2.4)
44/48 h*	42.4 (7.3)	75.0 (45.2)	62.33 (45.7)	10.0 (12.0)	2.5 (2.6)	2.3 (2.6)	6.7 (12.5)
72 h	48.0 (22.2)	74.8 (40.3)	123.8 (52.8)	7.8 (8.4)	19.8 (29.1)	28.3 (66.0)	48.2 (111.6)
5 d /120h	44.25 (30.8)	69.1 (32.6)	124.7 (32.8)	5.8 (5.2)	8.2 (10.8)	28.9 (41.4)	33.5 (73.2)
10 d/240h				2.4 (2.2)	5.2 (5.0)	21.1 (23.1)	38.5 (30.6)
14 d/336h	28.3 (13.2)	34.6 (10.5)	79.9 (23.1)				
28 d/672h	26.5 (20.7)	27.1 (13.7)	47.5 (11.5)	2.0 (3.8)	2.2 (2.0)	6.6 (10.8)	4.8 (2.9)

*In CKD patients, Iron and TSAT were assessed at 5, 19 and 44 hours, in healthy volunteers at 6, 18 and 48 hours

### Slow response Pharmacodynamic Parameters (ret, Hb, retHb)

Reticulocyte count as well as reticulocyte and erythrocyte hemoglobin did not appear to be affected by single administration of PRS-080#022 in both trials (S8 Table).

### Immunogenicity (development of ADA)

In the HV study 12 samples were identified (screening assay) and confirmed as potentially positive for ADA, they were therefore analyzed in a titration assay. Five (Day 28) samples were shown positive for anti-PRS-080 antibodies with titers of 1:400 (n = 4, one subject in the 4mg/kg, one in the 8 and 2 in the 16mg/kg dose groups) to 1:1600 (n = 1 in the 8mg/kg dose group).

In the CKD study the assay for anti-PRS-080#22 specific antibodies did not show a positive result in any of the dose groups.

## Discussion and conclusion

Elevated levels of hepcidin, often related to inflammation, are considered to have a major causal role in CKD anemia, as hepcidin suppresses both iron uptake and availability, and hence, erythropoiesis. In epidemiologic studies hepcidin levels correlate with renal anemia [25], and hepcidin knockout mice are protected from renal anemia [26]. Therefore, direct hepcidin antagonism may represent a potentially effective therapeutic approach to ameliorating renal anemia and its concurrent morbidities. Here we have presented first-in-human data with the novel and highly selective hepcidin antagonist PRS-080#022, demonstrating safety, pharmacokinetics, and pharmacodynamics of this novel compound.

In two single ascending dose trials, IV PRS-080#022 administrations were well tolerated up to the highest tested dose of 16 mg/kg in healthy volunteers (HV) and up to the highest tested dose of 8 mg/kg in end-stage CKD patients undergoing hemodialysis. Most AEs were mild and there was only one SAE (worsening of dry gangrene in a patient with a long history of diabetic foot syndrome, which was considered possibly related) reported in the CKD study. There were no clinically significant changes in normal laboratory values including liver transaminases. Local tolerance was excellent, with no infusion related reactions reported. Inflammatory cytokines, which were only assessed in the HV study, remained unaffected.

PRS-080#022 also mediated the expected pharmacodynamic effects of dose-dependently mobilizing serum iron with increases in both serum iron concentration and TSAT. This suggests that the mobilized iron was almost entirely transferrin bound and therefore highly functional, which favorably differentiates this therapeutic approach from IV iron administration.

Serum ferritin levels were largely unaffected by the treatment, and as ferritin is an acute phase protein and plays different roles apart from iron storage and transport [27] it is likely that the characteristics of subject populations have more impact on ferritin changes than the hepcidin antagonism. Nevertheless, the treatment with PRS-080#022 did not deplete body iron stores.

In our studies, no consistent dose-related changes in the slow response parameters Hb, Ret, and RetHb were shown after a single administration of PRS-080#022; a further multi-dose study is in progress to show whether repeated administration can sustain enhanced iron mobilization sufficiently to generate new red blood cells and ameliorate anemia.

The observations that AUCs for free PRS-080#022 Anticalin protein were considerably higher in the healthy volunteer study and that free PRS-080#022 increased slightly more than dose-proportionally (especially in the lower dose groups), whereas total PRS-080#022 showed a more dose proportional increase in AUC’s, could be explained by the low levels of hepcidin in the healthy volunteers. It can be assumed that the initial phase represents a protein binding process which is not yet saturated with the lowest doses of 1.2 and 2 mg/kg; this process becomes more saturated with increasing doses. From a safety perspective this finding is quite relevant because it contributes to the safety profile of the compound; i.e. with higher doses the pharmacokinetics become more linear, which is supported by the results of the regression analysis for total PRS-080#022. This analysis did not show a deviation from the proportionality assumption for AUC_0-t_, AUC_0-∞_, and C_max_. However, it has to be noted that the small sample size does not allow for definitive conclusions. Based on preclinical animal data it is known that the main elimination route of PRS-080 is via the kidneys [18]; therefore, the difference in elimination half-life between healthy volunteers (approx.3 days) and patients with end stage renal disease on hemodialysis (approx. 10–12 days) would be expected. The simulations based on the 2-compartmental PK evaluation show an accumulation factor of 2.5, with steady state reached after 5–6 doses. Therefore 5 administrations of PRS-080#22-DP given at weekly intervals in a multiple dose study should not exceed the plasma concentrations of total and free PRS-080#022-DP measured after a single administration of 16 mg/kg in healthy volunteers.

Hepcidin plasma levels showed some variation at baseline, especially in the CKD study where there was a decline between Screening and Day 1 (pre-dose). Hepcidin is known to follow a diurnal rhythm [28,29], but the CKD patients were treated and evaluated following their regular dialysis scheme (usually at the same time of the day). The protocol however required that iron administration be stopped one week before treatment, which could have had an influence on the treatment day 1 hepcidin values.

The dose related rebound effect of hepcidin plasma concentrations in both studies followed as expected the increase of iron and TSAT. This has also been observed with other hepcidin antagonists such as Lexaptepid and the monoclonal antibody LY2787106 [30,31]. It is known that hepcidin levels are physiologically increased by a homeostatic feedback loop in response to elevated plasma iron concentrations and cellular iron stores [32,33]. Chronic inflammation, which is often present in patients with CKD [17–19], may contribute to hepcidin induction in the CKD patients as the rebound was prolonged relative to the healthy volunteers. It remains to be determined in further clinical trials whether this rebound effect can be suppressed by repeated administrations of PRS-080#022.

Anti-drug-antibodies were assessed before treatment and at day 28, as preclinical studies have shown that ADA to PRS-080#22 do not develop before day 21. In the healthy volunteer study ADA were detected in 5 of 36 subjects (14%), which were considered unlikely to interfere with the hepcidin neutralizing activity of the drug. In the healthy volunteer study ADA were detected in 5 of 36 subjects (14%), which were low titer and considered unlikely to interfere with the hepcidin neutralizing activity of the drug. The ADA rate observed for monoclonal antibodies has been reported to range between 0 and 12% for fully human products [34] and the study with LY2787106 showed an ADA rate of 24% [31]. The incidence of ADA is dependent on the immune status of the subject and expected to be higher in healthy volunteers and immuno-competent patients compared to patients with a compromised immune system [35]. The test for Anti-PRS-080#22 specific antibodies in the CKD study did not show a positive result in any of the dose groups, potentially due to alterations in the immune system in end stage renal disease, as uremia is associated with a state of immune dysfunction [36]. Therefore, the immunogenic potential of PRS-080#022 in CKD patients appears to be low.

In summary, the excellent safety profile and the confirmed activity of PRS-080#022 on iron metabolism in anemic, dialysis dependent, end-stage CKD patients warrant further investigation of PRS-080#022 in a multiple dosing regimen to explore potential amelioration of anemia in these patients. A multiple-ascending dose study in dialysis dependent CKD5 patients is currently underway.

## Supporting information

S1 Protocol Study PCS-01-012 in healthy volunteers. (PDF)

S2 Protocol Study PCS-02-015 in CKD patients. (PDF)

S1 Fig Free Anticalin, time-concentration curve in CKD patients, arithmetic mean, linear-linear. (JPG)

S2 Fig Free Anticalin, time-concentration curve in healthy volunteers, arithmetic mean, linear-linear. (JPG)

S3 Fig Average simulated plasma concentration time curves of free PRS-080 #022 (semi-logarithmic scale) in CKD patients. (PDF)

S4 Fig Free Hepcidin, time-concentration curve in healthy volunteers. (PNG)

S5 Fig Free Hepcidin, time-concentration curve in CKD patients. (PNG)

S1 Table Patient disposition, healthy volunteers. (PDF)

S2 Table Patient disposition, CKD patients. (PDF)

S3 Table PK parameters, healthy volunteers, free and total Anticalin. (PDF)

S4 Table PK parameters, CKD patients, free and total Anticalin. (PDF)

S5 Table Iron and TSAT, absolute mean values for healthy volunteers and CKD patients. (PDF)

S6 Table Ferritin (ng/mL), change from baseline for healthy volunteers and CKD patients. (PDF)

S7 Table Free Hepcidin healthy volunteers. (PDF)

S8 Table Free Hepcidin CKD patients. (PDF)

S9 Table Slow response parameters/change from baseline for healthy volunteers and CKD patients. (PDF)
