# Supplementary material for: First-in-human Phase I studies of PRS-080#22, a hepcidin antagonist, in healthy volunteers and patients with chronic kidney disease undergoing hemodialysis
Source: PLoS One. 2019 Mar 27;14(3):e0212023. doi: 10.1371/journal.pone.0212023 (PMC6436791; doi:10.1371/journal.pone.0212023)
Supplement: S1 Protocol — (PDF) [file pone.0212023.s001.pdf]

# PROTOCOL

## FIRST-IN-HUMAN STUDY TO EVALUATE THE SAFETY, TOLERABILITY, PHARMACOKINETICS AND PHAMACODYNAMICS OF PRS-080 (HEPCIDIN ANTAGONIST).

## "UMBRELLA PROTOCOL" FOR THE SAD AND MAD STAGES WITH PRS-080#022-DP DOSED BY IV ADMINISTRATION.

**Stage 1**: A First-in-Human (FIH), Randomized, Dose-Escalation, Double-Blind, Placebo-Controlled single ascending dose study (SAD) in healthy volunteers to establish safety, lack of immunogenicity, tolerability, pharmacokinetic parameters, dose-dependent target engagement and pharmacodynamic effects.

**Stage 2**: Randomized, Dose-Escalation, Double-Blind, Placebo-Controlled multiple ascending dose study (MAD) in healthy volunteers to establish safety, lack of immunogenicity, tolerability, pharmacokinetic parameters, dose-dependent target engagement and pharmacodynamic effects.

| | |
|---|---|
| EudraCT number: | 2012-001450-26 |
| Sponsor's study code: | PCS_01_12 |
| Nuvisan study code: | N-A-PH1-11-014 |
| Phase: | Phase I |
| Names of investigational product(s): | Test drug: PRS-080#022-DP<br>Reference drug: Placebo |
| Principal Investigator: | Dr. med. Werner Feuerer Nuvisan GmbH<br>Wegenerstraße 13<br>89231 Neu-Ulm<br>Germany |
| Sponsor: | PIERIS AG<br>Lise-Meitner-Strasse 30<br>85354 Freising-Weihenstephan<br>Germany |
| Version/Date: | 3.0 / 20.02.2014 |

This protocol is the property of Nuvisan GmbH. The recipient agrees that no unpublished information contained herein will be published or disclosed without prior written approval of Nuvisan GmbH, except that this protocol may be disclosed to the appropriate Independent Ethics Committee or Institutional Review Board and appropriate Authority(ies).
The rights to the information in this protocol and the resulting study become the property of the Sponsor only upon completion of contractual obligations of Nuvisan GmbH and the Sponsor.

## SIGNATURE PAGE CRO

The people signing hereby declare that they have read this protocol and agree to its contents. They confirm that the study will be conducted and documented in full accordance with the protocol, standards of Good Clinical Practice specified herein, and the national drug law.

**Principal Investigator:**

Dr. med. Werner Feuerer
Nuvisan GmbH — Date (dd-MMM-yyyy) — Signature

**Responsible Investigator:**

Michael Lissy, Physician
Nuvisan GmbH — Date (dd-MMM-yyyy) — Signature

**Responsible for Bioanalytics:**

Dr. Eddy Bruyns
Nuvisan GmbH — Date (dd-MMM-yyyy) — Signature

**Responsible for the Clinical Laboratory:**

Dr. Jutta Michael-Hepp
Nuvisan GmbH — Date (dd-MMM-yyyy) — Signature

**Statistician:**

Dr. Dirk Lehnick
Nuvisan GmbH — Date (dd-MMM-yyyy) — Signature

## SIGNATURE PAGE SPONSOR

The people signing hereby declare that they have read this protocol and agree to its contents. They confirm that the study will be conducted and documented in full accordance with the protocol, standards of Good Clinical Practice specified herein, and the national drug law.

**Sponsor's responsible representatives:**

| | | |
|---|---|---|
| Dr. Shane Olwill<br>(VP Development, Pieris AG) | Date (dd-MMM-yyyy) | Signature |
| Dr. Ulrich Moebius<br>(CSO, Pieris AG) | Date (dd-MMM-yyyy) | Signature |
| Dr. Edgar Fenzl<br>(Managing Director FGK,<br>QPPV Pieris AG) | Date (dd-MMM-yyyy) | Signature |

## LIST OF ABBREVIATIONS AND DEFINITION OF TERMS

| | | |
|---|---|---|
| $\lambda_z$ | = | terminal rate constant |
| AAI | = | Applied Analytical Industries |
| AB | = | Antibodies |
| ACD | = | anemia of chronic disease |
| ADA | = | Anti-drug antibodies |
| ADR | = | Adverse drug reaction |
| Ae | = | Amount excreted into the urine |
| AE | = | Adverse event |
| ALT/GPT | = | alanine-amino-transferase |
| ALB | = | albumin |
| ALC | = | ethanol |
| AP | = | alkaline phosphatase |
| AMG | = | Arzneimittelgesetz (German Drug Law) |
| AMPH | = | amphetamines |
| ANOVA | = | analysis of variance |
| APTT | = | activated partial thromboplastin-time |
| AST/GOT | = | aspartate-amino-transferase |
| ATC | = | Anatomical Therapeutic Chemical Classification |
| AUC | = | area under the curve |
| $AUC_{0\text{-}t}$ | = | area under the concentration time curve (time 0 to last sample with a quantifiable concentration) |
| $AUC_{0\text{-}\tau}$ | = | area under the curve over a dosing interval at steady-state |
| $AUC_{0\text{-}\infty}$ | = | area under the concentration time curve from time 0 extrapolated to infinity |
| BA | = | bioavailability |
| BARB | = | barbiturates |
| BASO | = | basophil leukocytes |
| BE | = | bioequivalence |
| BENZO | = | benzodiazepines |
| BfArM | = | Bundesinstitut für Arzneitmittel und Medizinprodukte (German Federal Insitute for Drugs and Medical Devices) |
| BILI | = | bilirubin |
| BMI | = | body mass index |
| BP | = | blood pressure |
| BUN | = | blood urea nitrogen |
| BW | = | body weight |
| Ca | = | calcium |
| CA | = | competent authority(ies) |
| CANNAB | = | cannabinoides |
| CHE | = | cholinesterase |
| CHOL | = | cholesterol |
| CK | = | creatine kinase |
| Cl | = | chloride |
| $CL_R$ | = | renal clearance |
| CL | = | clearance (after i.v. infusion) |
| $C_{max}$ | = | maximal concentration |
| CREA | = | creatinine |
| CRF | = | case report form |

| | | |
|---|---|---|
| CRO | = | contract research organization |
| CTS | = | Clinical Trial Supply |
| DD | = | drug dictionary |
| DEC | = | Dose Escalation Committee |
| EC | = | European Community |
| ECG | = | electrocardiogram |
| EDTA | = | ethylene-diamine-tetra-acetate |
| EENT | = | eyes, ears, nose and throat |
| EMA | = | European Medicines Agency |
| EOS | = | eosinophil leukocytes |
| ERY | = | blood (in urinalysis) |
| ESA | = | erythropoietin stimulating agents |
| ESR | = | erythrocyte sedimentation rate |
| FDA | = | Food and Drug Administration |
| FERR | = | Ferritin |
| FIH | = | First-in-human |
| GCMS | = | Gas chromatography mass spectrometry |
| GCP | = | Good Clinical Practice |
| G-GT | = | gamma-glutamyl-transferase |
| GLM | = | general linear models |
| GLOB | = | Globulin |
| GLUC | = | glucose |
| GMP | = | Good Manufacturing Practice |
| GOT (AST) | = | glutamate oxaloacetate transaminase (aspartate-amino-transferase) |
| GPT (ALT) | = | glutamate pyruvate transaminase (alanine-amino-transferase) |
| Hb | = | hemoglobin |
| HBs-AG | = | hepatitis B antigen |
| HCV | = | hepatitis C virus |
| HCT | = | hematocrit |
| HED | = | human equivalent dose |
| HIV | = | Human immunodeficiency virus |
| HPLC | = | high performance liquid chromatography |
| HR | = | heart rate |
| hs CRP | = | high sensitivity C-reactive protein (assay) |
| $IC_{50}$ | = | half maximal inhibitory concentration |
| IEC | = | Independent Ethics Committee |
| IMP | = | Investigational Medicinal Product |
| IRB | = | Institutional Review Board |
| ITT | = | intention-to-treat |
| Investigator | = | A doctor or a person following a profession agreed in the Member State for investigations because of the scientific background and the experience in patient care it requires |
| i.v. | = | intravenous |
| K | = | potassium |
| KETON | = | ketones |
| L | = | Liter |
| LCMS | = | Liquid chromatography mass spectrometry |
| LDH | = | Lactate dehydrogenase |
| LEUCO | = | leukocytes |

| | | |
|---|---|---|
| LYMPH | = | lymphocytes |
| MAD | = | multiple ascending dose |
| MCH | = | mean corpuscular hemoglobin |
| MCHC | = | mean corpuscular hemoglobin concentration |
| MCV | = | mean corpuscular volume |
| MedDRA | = | Medical Dictionary for Regulatory Activities |
| MONO | = | monocytes |
| MRT | = | mean residence time |
| MS | = | mass spectrometry |
| MTD | = | maximum tolerated dose |
| Na | = | sodium |
| NEUT | = | neutrophil leukocytes |
| NGAL | = | neutrophil gelatinase-associated lipocalin |
| NITRIT | = | nitrites |
| NOAEL | = | No-Observed-Adverse-Effect Level |
| OPIATE | = | opiates |
| PBC | = | platelet blood count |
| PD | = | pharmacodynamics |
| PEG | = | polyethylene glycol |
| pH | = | hydrogen ion concentration |
| PI | = | Principle Investigator (A person/investigator that bears the overall responsibility for the conduct of the clinical trial at the clinical site) |
| PK | = | pharmacokinetics |
| PROT | = | protein |
| QAg | = | Quality Agreement |
| QUICK INR | = | prothrombin time |
| RBC | = | red blood count |
| Responsible Investigator | = | Leader responsible for the team involved in the conduct of a clinical trial at the trial site. |
| RIA | = | radioimmunoassay |
| SAD | = | single ascending dose |
| SAE | = | serious adverse event |
| SAR | = | serious adverse reaction |
| SOP | = | standard operating procedure |
| SP-WEIGHT | = | specific weight |
| SUSAR | = | suspected unexpected serious adverse reaction |
| $t_{½}$ | = | terminal half-life |
| T-BILI | = | total bilirubin |
| TRSF | = | transferrin |
| TG | = | triglyceride |
| $t_{max}$ | = | time of observed maximum concentration |
| TP | = | total protein |
| URIC | = | uric acid |
| UROBIL | = | urobilinogen |
| $V_z$ | = | volume of distribution (after i.v. infusion) |
| VEGF-A | = | vascular endothelial growth factor A |
| WBC | = | white blood count |
| WHO | = | World Health Organization |

## SYNOPSIS

| | |
|---|---|
| Study Title | First-in-Human Study to Evaluate the Safety, Tolerability, Pharmacokinetics and Pharmacodynamics of PRS-080 (Hepcidin Antagonist). |
| Stage description | Stage 1: A First-in-Human (FIH), Randomized, Dose-Escalation, Double-Blind, Placebo-Controlled single ascending dose study (SAD) in healthy volunteers to establish safety, lack of immunogenicity, tolerability, pharmacokinetic parameters, dose-dependent target engagement and pharmacodynamic effects.<br>Directly after completion and evaluation of the SAD a repeat dose stage will follow titled:<br>Stage 2: Randomized, Dose-Escalation, Double-Blind, Placebo-Controlled multiple ascending dose (MAD) in healthy volunteers to establish safety, lack of immunogenicity, tolerability, pharmacokinetic parameters, dose-dependent target engagement and pharmacodynamic effects. |
| Investigational product(s) | Test drug: PRS-080#022-DP<br>Reference drug: Placebo |
| Study design | **Single Ascending Dose (SAD):**<br>• First-in-Human (FIH), Randomized, Dose-Escalation, Double-Blind, Placebo-Controlled Single Dose in healthy volunteers.<br>The single rising dose stage will enrol 8 subjects per cohort (6 verum, 2 placebo), up to a maximum tolerated dose, defined by the stopping rules. 6 dose levels are anticipated. Study drug will be administered as a 120 min i.v. infusion on Day 1. The decision to escalate the dose by the dose escalation committee (DEC) will be based on an interim analysis of clinical safety and safety laboratory data 24 hours and 72 hours after the start of infusion.<br><br>**Multiple Ascending Dose (MAD):**<br>• Randomized, Dose-Escalation, Double-Blind, Placebo-Controlled Repeat Dose in healthy volunteers.<br>The multiple rising dose stage will enrol 2 cohorts of 12 subjects each (9 verum, 3 placebo). Dose levels will be limited to 1 to 2 dose levels below the MTD of the SAD stage or, if no MTD was observed, 1 dose level below the highest dose group tested. Study drug will be administered over 13 days (7 times, every second day) as a 120 min i.v. infusion to reach steady state. The dosing frequency will be adjusted depending on the PK/PD relationship established during the SAD phase (human half-life, target inhibition and iron response). |

| | |
|---|---|
| | **The following assessments will be performed in each of the two stages:**<br>Safety and tolerability (adverse events, vital signs, 12-lead ECGs, respiration rate, and local tolerability assessment)<br>Body temperature (ear)<br>Blood sampling for Anticalin pharmacokinetics in plasma<br>Urine sample collection for eventual PK purposes will be taken and stored for analysis during the SAD-stage only.<br>Blood sampling for target engagement (hepcidin) in serum<br>Urine and blood sampling for clinical safety laboratory including high-sensitivity C-reactive protein (hs CRP)<br>Blood sampling for pharmacodynamic markers with a fast response kinetic (i.e. iron, transferrin saturation, and ferritin) in serum<br>Blood sampling for pharmacodynamic markers with a slow response kinetic (i.e. Hb, reticulocytes, reticulocyte Hb) in whole blood<br>Blood samples will be taken and stored for analysis of protein based biomarkers if warranted by the results of clinical safety laboratory, hepcidin or pharmacodynamic marker analysis<br>Blood sampling for ADA responses (immunogenicity). |
| Objectives | Primary objectives:<br>• to determine the safety and maximum tolerated dose (MTD) of single and repeated intravenous infusion with PRS-080#022-DP<br>• to collect data concerning pharmacokinetics of PRS-080#022-DP upon intravenous dose<br>• to collect data concerning clinical safety and tolerability<br>• to collect data on immunogenicity<br>Secondary objectives:<br>• to evaluate pharmacodynamic parameters including dose-dependent iron response<br>• to evaluate dose-dependent target engagement (hepcidin)<br>The results will be used to define safe and potentially effective dose levels for a phase IIa study in patients with anemia for therapeutic proof of concept. |
| Primary variable(s) | Safety: AEs, vital signs (BP, HR), ECG, laboratory parameters including hs CRP after single and multiple dose administration. |
| Additional variables | Secondary variables:<br>Anticalin Pharmacokinetics: $C_{max}$, $t_{max}$, $AUC_{0-t}$, $AUC_{0-\infty}$, $AUC_{0-\tau}$, $t_{1/2}$, MRT, after single and multiple ascending dose administration.<br>ADA responses (immunogenicity) after single and multiple ascending dose administration |

| | |
|---|---|
| | Target engagement: serum hepcidin after single and multiple ascending dose administration<br>Pharmacodynamics: iron response markers including but not limited to total iron, transferrin saturation, Hb, reticulocytes, and reticulocyte Hb, after single and multiple ascending dose administration. |
| Treatments | Test treatment: PRS-080#022-DP<br>Formulation: PBS pH 6.5 (20 mM $NaH_2PO_4$; 115 mM NaCl; pH 6.5)<br>Strength: 8.1 mg/mL<br>Reference treatment: Placebo (PBS pH 6.5 formulation)<br>Stage 1: Single intravenous administration of PRS-080#022-DP/placebo over a dose range of 0.08 to 16 mg/kg BW.<br>Stage 2: Multiple intravenous doses (7 administrations) of PRS-080#022-DP/placebo over 2 dose levels (dose level and frequency determined by Stage 1 results) |
| Subject population | Subjects should be healthy Caucasian male subjects, with a BMI 18-30 kg/m$^2$ and body weight 60-90 kg inclusive, 18-50 years old. Subjects with iron overload or disturbance in utilization of iron, or who have received i.v. iron treatment or blood transfusion within last 90 days prior to the planned first drug administration or during trial will be excluded. |
| Number of subjects | Single Ascending Dose Study (SAD): Stage 1<br>6 or more cohorts of 8 subjects each (6 verum, 2 placebo).<br>Multiple Ascending Dose Study (MAD): Stage 2<br>2 or more cohorts of 12 subjects each (9 verum, 3 placebo). |
| Number of centers | One |
| Planned time schedule | JAN 2014 – AUG 2014 |

# STUDY DESIGN AND SCHEDULE OF ASSESSMENTS

STUDY DESIGN – Stage 1 Single ascending dose (SAD) study

**DOSE COHORT 1 (0.08 mg/kg BW)**

**SUBGROUP 1**
**1x Verum**
**1x Placebo**

based on 24 h safety data →

**SUBGROUP 2**
**1-2x Verum**
**0-1x Placebo**

**150 min between start of dosing individuals**

based on 24 h safety data →

**SUBGROUPS 3 and 4**
**1-2x Verum**
**0-1x Placebo**
(per subgroup)

**150 min between start of dosing**

→

**DEC MEETING**

based on 72 h safety data

**NEXT DOSE / STOP / INTERMEDIATE DOSE**

**APPROX. 1 WEEK**

→

**DOSE COHORTS 2 – 6 (0.4 mg/kg BW – 16 mg/kg BW)**

**SUBGROUP 1**
**1x Verum**
**1x Placebo**

based on 24 h safety data →

**SUBGROUPS 2 and 3**
**2-3x Verum**
**0 -1x Placebo**
(per subgroup)

**150 min between start of dosing individuals**

→

**DEC MEETING**

based on 72 h safety data

**NEXT DOSE / STOP / INTERMEDIATE DOSE**

**APPROX 1 WEEK**

STUDY DESIGN – Stage 2 Multiple ascending dose (MAD) study

**DOSE COHORT 1** **DOSE COHORT 2**

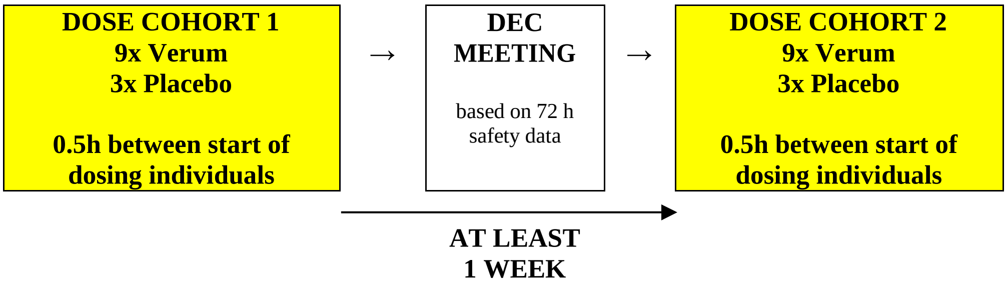

# Table 1: Flow Chart – Stage 1

| **Flow Chart - Stage 1** | **SCR** | | | | | | | | | | | | | | | | | | | | **6 dose levels (cohorts)** | | | **8 Subjects per dose level (cohort)** | | | |
|---|---|---|---|---|---|---|---|---|---|---|---|---|---|---|---|---|---|---|---|---|---|---|---|---|---|---|---|
| Relative time [Day] | ≤ 21 | | Day 1 | | | | | | | | | | | | | | | | | | 2 | | 3 | 4 | 6 | 11 | Day 28 ± 2 |
| Relative Time [hour] | | -24 | 0 | 0.25 | 0.5 | 0.75 | 1 | 1.25 | 1.5 | 1.75 | 2 | 2.5 | 3 | 3.5 | 4 | 6 | 8 | 10 | 12 | 18 | 24 | 36 | 48 | 72 | 120 | 240 follow up | Additional visit |
| Hospitalization days From | | x | | | | | | | | | | | | | | | | | | | | | | | | | |
| To | | | | | | | | | | | | | | | | | | | | | | | | x | | | |
| Ambulatory visits | | | | | | | | | | | | | | | | | | | | | | | | | x | x | x |
| Informed Consent meeting | x | | | | | | | | | | | | | | | | | | | | | | | | | | |
| Alcohol breath test | x | x | | | | | | | | | | | | | | | | | | | | | | | | | |
| Demographic data | x | | | | | | | | | | | | | | | | | | | | | | | | | | |
| Medical history | x | | | | | | | | | | | | | | | | | | | | | | | | | | |
| Height, weight (BMI) | x | | | | | | | | | | | | | | | | | | | | | | | | | | |
| Weight for dose calculation | | x | | | | | | | | | | | | | | | | | | | | | | | | | |
| Inclusion/exclusion criteria | x | | | | | | | | | | | | | | | | | | | | | | | | | | |
| Adverse event questioning | x | x | x | x | x | x | x | x | x | x | x | x | x | x | x | | x | | x | | x | | x | x | x | x | x |
| Concomitant medication | x | x | x | | x | | x | | | | x | | | | x | | x | | x | | x | | x | x | x | x | x |
| Vital signs (blood pressure + pulse rate) | x | | x | x | x | x | x | x | x | x | x | x | x | x | x | | x | | x | | x | | x | x | x | x | |
| Body temperature (ear) | x | | x | x | x | x | x | x | x | x | x | x | x | x | x | | x | | x | | x | | x | x | x | x | |
| Respiratory rate | x | | x | x | x | x | x | x | x | x | x | x | x | x | x | | x | | x | | x | | x | x | x | x | |
| ECG (12-lead) | x | | x | | x | | x | | | | x | | | | x | | x | | x | | x | | x | x | x | x | |
| Drug administration (i.v.) | | | x | | | 120 min | | | | | | | | | | | | | | | | | | | | | |
| Physical examination | x | | | | | | | | | | | | | | | | | | | | | | | | | x | |
| Local tolerability | | | x | | x | | x | | | | x | | | | x | | x | | x | | x | | x | x | x | x | |
| Biochemistry | x | x | | | | | | | | | | | | | | | | | | | x | | | x | x | x | |
| Hematology | x | x | | | | | | | | | | | | | | | | | | | x | | | x | x | x | |
| Coagulation | x | x | | | | | | | | | | | | | | | | | | | x | | | x | x | x | |
| Urinalysis | x | x | | | | | | | | | | | | | | | | | | | x | | | x | x | x | |
| Serology | x | | | | | | | | | | | | | | | | | | | | | | | | | | |
| Drug screening | x | x | | | | | | | | | | | | | | | | | | | | | | | | | |
| Blood sampling for Anticalin PK | | | x | | | | x | | | | x | | x | | x | x | | x | | x | x | x | x | x | x | x | |
| Blood sampling for Hepcidin | x | x | x | | | | x | | | | x | | x | | x | x | | x | | x | x | x | x | x | x | x | x |
| Blood sampling for PD (i.e. iron, transferrin saturation and ferritin) | x | x | x | | | | | | | | x | | | | x | | | x | | x | x | x | x | x | x | x | x |
| Blood sampling for PD (i.e. Hb, reticulocytes, reticulocyte Hb) | x | | x | | | | | | | | | | | | | | | x | | | x | | x | x | x | x | x |
| Blood samples biomarker analysis | | | x | | | | | | | | x | | | | | x | | | | | x | | | x | | | |
| Urine sampling for Anticalin PK (pooled and discrete samples) | | | x | | | | | | | | | | | | | | | | | | x | | x | x | | | |
| Blood sampling for ADA | | | x | | | | | | | | | | | | | | | | | | | | | | | | x |

# Table 2: Flow Chart – Stage 2

| Flow Chart - Stage 2 | | 2 dose levels (cohorts) | | | | | | | | | | | | | | | | | | | | | | | | | | | | | 12 Subjects per dose level (cohort) | | | | | | | | | | | | | | | |
|---|---|---|---|---|---|---|---|---|---|---|---|---|---|---|---|---|---|---|---|---|---|---|---|---|---|---|---|---|---|---|---|---|---|---|---|---|---|---|---|---|---|---|---|---|---|---|
| Time relative to dosing[hour] | SCR | -24 | 0 | 0,5 | 1 | 2 | 3 | 4 | 6 | 8 | 10 | 12 | 18 | 24 | 36 | 0 | 4 | | 0 | 4 | | 0 | 4 | | 0 | 4 | | 0 | 4 | | 0 | 0,5 | 1 | 2 | 4 | 6 | 10 | 12 | 18 | 24 | 36 | 48 | 72 | 120 | 240 | 672 |
| Study Day [Day] | ≤ 21 | -1 | 1 | 1 | 1 | 1 | 1 | 1 | 1 | 1 | 1 | 1 | 1 | 2 | | 3 | 3 | 4 | 5 | 5 | 6 | 7 | 7 | 8 | 9 | 9 | 10 | 11 | 11 | 12 | 13 | 13 | 13 | 13 | 13 | 13 | 13 | 13 | 13 | 14 | 14 | 15 | 16 | 18 | 23 | 41 ±2 |
| Hospitalization days from | | x | | | | | | | | | | | | | | | | | | | | | | | | | | | | | | | | | | | | | | | | | | | | |
| to | | | | | | | | | | | | | | | | | | | | | | | | | | | | | | | | | | | | | | | | | | | x | | | |
| Ambulatory visits | | | | | | | | | | | | | | | | | | | | | | | | | | | | | | | | | | | | | | | | | | | | x | x | x |
| Informed Consent meeting | x | | | | | | | | | | | | | | | | | | | | | | | | | | | | | | | | | | | | | | | | | | | | | |
| Alcohol breath test | x | x | | | | | | | | | | | | | | | | | | | | | | | | | | | | | | | | | | | | | | | | | | | | |
| Demographic data | x | | | | | | | | | | | | | | | | | | | | | | | | | | | | | | | | | | | | | | | | | | | | | |
| Medical history | x | | | | | | | | | | | | | | | | | | | | | | | | | | | | | | | | | | | | | | | | | | | | | |
| Height, weight (BMI) | x | | | | | | | | | | | | | | | | | | | | | | | | | | | | | | | | | | | | | | | | | | | | | |
| Weight for dose calculation | | x | | | | | | | | | | | | | | | | | | | | | | | | | | | | | | | | | | | | | | | | | | | | |
| Inclusion/exclusion criteria | x | | | | | | | | | | | | | | | | | | | | | | | | | | | | | | | | | | | | | | | | | | | | | |
| Adverse event questioning[a] | x | x | x | x | x | x | | x | | x | | x | | x | | x[a] | x | x | x[a] | x | x | x[a] | x | x | x[a] | x | x | x[a] | x | x | x | | x | x | x | | | | | x | | x | x | x | x | x |
| Concomitant medication | x | x | x | x | x | x | | x | | x | | x | | x | | x | x | x | x | x | x | x | x | x | x | x | x | x | x | x | x | | x | x | x | | | | | x | | x | x | x | x | x |
| Vital signs (blood pressure + pulse rate)[a] | x | | x | x | x | x | | x | | x | | x | | x | | x[a] | x | x | x[a] | x | x | x[a] | x | x | x[a] | x | x | x[a] | x | x | x | | x | x | x | | | | | x | | x | x | x | x | |
| Body temperature (ear) [a] Every 15 min during infusion on day 1 and at | x | | x | x | x | x | | x | | x | | x | | x | | x[a] | x | x | x[a] | x | x | x[a] | x | x | x[a] | x | x | x[a] | x | x | x | | x | x | x | | | | | x | | x | x | x | x | |
| Respiratory rate [a] | x | | x | x | x | x | | x | | x | | x | | x | | x[a] | x | x | x[a] | x | x | x[a] | x | x | x[a] | x | x | x[a] | x | x | x | | x | x | x | | | | | x | | x | x | x | x | |
| ECG (12-lead)[a] | x | | x | x | x | x | | x | | x | | x | | x | | x[a] | x | x | x[a] | x | x | x[a] | x | x | x[a] | x | x | x[a] | x | x | x | | x | x | x | | | | | x | | x | x | x | x | |
| Drug administration i.v. (120min) | | | x | 120 min | | x | | | | | | | | | | x | | | x | | | x | | | x | | | x | | | x | 120 min | | x | | | | | | | | | | | | |
| Physical examination | x | | | | | | | | | | | | | | | | | | | | | | | | | | | | | | | | | | | | | | | | | | | | x | |
| Local tolerability | | | x | | x | | | x | | | | x | | x | | x | x | | x | x | | x | x | | x | x | | x | x | | x | | | | x | | | x | | x | | | | | x | |
| Biochemistry | x | x | | | | | | | | | | | | x | | | | x | | | | | | x | | | | | | x | | | | | | | | | | x | | | x | x | x | |
| Hematology | x | x | | | | | | | | | | | | x | | | | x | | | x | | | x | | | x | | | x | | | | | | | | | | x | | | x | x | x | |
| Coagulation | x | x | | | | | | | | | | | | x | | | | x | | | | | | x | | | | | | x | | | | | | | | | | x | | | x | x | x | |
| Urinalysis | x | x | | | | | | | | | | | | x | | | | x | | | | | | x | | | | | | x | | | | | | | | | | x | | | x | x | x | |
| Serology | x | | | | | | | | | | | | | | | | | | | | | | | | | | | | | | | | | | | | | | | | | | | | | |
| Drug screening | x | x | | | | | | | | | | | | | | | | | | | | | | | | | | | | | | | | | | | | | | | | | | | | |
| Blood sampling for Anticalin PK [b] | | | x | | x | x | x | x | x | | x | | x | x | x | x | | | x[b] | | | x[b] | | | x[b] | | | x[b] | | | x | | x | x | x | x | x | x | x | x | x | x | x | x | x | |
| Blood sampling for Hepcidin [b] | x | x | x | | x | x | x | x | x | | x | | x | x | x | x | | | x[b] | | | x[b] | | | x[b] | | | x[b] | | | x | | x | x | x | x | x | x | x | x | x | x | x | x | x | x |
| Blood sampling for PD (e.g. iron and transferrin saturation and ferritin) [c] | x | x | x | | | x | | x | | | x | | x | x | x | x[c] | | | x[c] | | | x[c] | | | x[c] | | | x[c] | | | x[c] | | | x | x | | x | | x | x | x | x | x | x | x | x |
| Blood sampling for PD (e.g. Hb, reticulocytes, reticulocyte Hb) [c] | x | | x | | | | | | | | x | | | x | | x[c] | | | x[c] | | | x[c] | | | x[c] | | | x[c] | | | x[c] | | | | | | x | | | x | | x | | x | x | x |
| Blood samples biomarker analysis [c] | | | x | | | x | | | x | | | | | x | | x[c] | | | x[c] | | | x[c] | | | x[c] | | | x[c] | | | x | | | x | | x | | | | x | | | x | | | |
| Blood sampling for ADA | | | x | | | | | | | | | | | | | | | | | | | | | | | | | | | | | | | | | | | | | | | | | | | x |

a: Additional assessments of adverse events, vital signs, 12-lead ECGs, respiratory rate and body temperature are to be carried out prior to dosing (0 h) and at 1h and 2h after each study drug administration (i.e. Days 3, 5, 7, 9, 11 and 13)
b: Samples will be collected at 0 h (prior to dosing) and at 2 h (end of infusion), for trough and peak assessment
c: Samples will be collected at 0 h (prior to dosing)

# TABLE OF CONTENTS


**TABLE OF CONTENTS** ..... 14

**LIST OF TABLES** ..... 18

**1 INVESTIGATORS AND STUDY-ADMINISTRATIVE STRUCTURE** ..... 19
**1.1 REQUIREMENTS CONCERNING THE INVESTIGATOR(S) AND STUDY CENTER(S)** ..... 19
**1.2 DOSE ESCALATING COMMITTEE (DEC)** ..... 20
**1.3 ADMINISTRATIVE STRUCTURE** ..... 21
**1.4 REGULATIONS AND GUIDELINES** ..... 23

**2 INTRODUCTION** ..... 24
**2.1 BACKGROUND** ..... 24
2.1.1 ANEMIA OF CHRONIC DISEASE ..... 24
2.1.2 HEPCIDIN ..... 24
2.1.3 PRS-080 ..... 25
2.1.4 NON-CLINICAL DATA WITH PRS-080 ..... 25
**2.2 RATIONALE** ..... 26
**2.3 JUSTIFICATION FOR DOSES TO BE EVALUATED** ..... 26

**3 STUDY OBJECTIVES** ..... 27

**PRIMARY OBJECTIVES** ..... 27

**SECONDARY OBJECTIVES** ..... 27

**PRIMARY VARIABLES TO BE STUDIED IN DETAIL ARE** ..... 27

**SECONDARY VARIABLES TO BE STUDIED ARE** ..... 28

**4 ETHICS** ..... 28
**4.1 INDEPENDENT ETHICS COMMITTEE (IEC) AND COMPETENT AUTHORITY (CA)** ..... 28
**4.2 SUBJECT INFORMATION AND CONSENT** ..... 29
**4.3 GENERAL SAFETY** ..... 29
**4.4 BENEFIT-RISK EVALUATION** ..... 30

**5 OVERALL STUDY DESIGN AND PLAN** ..... 31
**5.1 OVERALL STUDY DESIGN** ..... 31
5.1.1 STAGE 1 ..... 31
5.1.2 STAGE 2 ..... 32
**5.2 OVERALL STUDY PLAN** ..... 33
5.2.1 SCREENING EXAMINATION ..... 33
5.2.2 STUDY PERFORMANCE ..... 34
5.2.2.1 Duration of Confinement Periods ..... 34

5.2.2.2 Day -1 ... 35
5.2.2.3 Treatment Phase – Pre-Dose ... 36
5.2.2.4 Treatment Phase – Post-Dose ... 36
5.2.2.5 Treatment Phase – Ambulatory Visits ... 37
5.2.3 END OF STUDY EXAMINATION ... 37

**6 DISCUSSION OF STUDY DESIGN, INCLUDING THE CHOICE OF CONTROL GROUPS ... 38**

**7 SELECTION OF STUDY POPULATION ... 39**
**7.1 INCLUSION CRITERIA ... 39**
**7.2 EXCLUSION CRITERIA ... 40**
**7.3 REMOVAL OF SUBJECTS FROM THERAPY OR ASSESSMENT ... 41**
7.3.1 PREMATURE DISCONTINUATION OF THE STUDY PER SUBJECT ... 41
7.3.2 PREMATURE DISCONTINUATION OF THE STUDY AT THE TRIAL SITE ... 43
7.3.3 PREMATURE DISCONTINUATION OF THE COMPLETE STUDY ... 43

**8 TREATMENTS ... 43**
**8.1 TREATMENTS TO BE ADMINISTERED ... 43**
**8.2 IDENTITY OF INVESTIGATIONAL PRODUCTS ... 44**
**8.3 METHOD OF ASSIGNING SUBJECTS TO TREATMENT GROUPS ... 45**
**8.4 SELECTION OF DOSES IN THE STUDY ... 46**
**8.5 SELECTION AND TIMING OF DOSE FOR EACH SUBJECT ... 46**
8.5.1 DOSE ESCALATION IN STAGE 1 AND STAGE 2 ... 47
8.5.2 STAGE 1: SINGLE ASCENDING DOSE STUDY STAGE (SAD) ... 48
8.5.3 STAGE 2: MULTIPLE ASCENDING DOSE STUDY STAGE (MAD) ... 48
**8.6 LABELING AND PACKAGING OF THE INVESTIGATIONAL PRODUCT(S) ... 49**
**8.7 BLINDING AND BREAKING THE BLIND ... 50**
**8.8 PRIOR AND CONCOMITANT THERAPY ... 51**
**8.9 TREATMENT COMPLIANCE ... 52**

**9 VARIABLES TO BE ASSESSED ... 52**
**9.1 EFFICACY VARIABLES ... 52**
**9.2 PHARMACODYNAMIC VARIABLES ... 52**
9.2.1 SUMMARY OF SUBSTANCES TO BE ANALYZED ... 52
9.2.2 TIME POINTS/INTERVALS FOR SAMPLE COLLECTION ... 53
9.2.3 SAMPLE HANDLING PROCEDURES ... 56
9.2.4 APPROPRIATENESS AND DESCRIPTION OF ANALYTICAL METHODS ... 57
9.2.5 BLOOD COLLECTION FOR PHARMACODYNAMIC ASSESSMENT ... 57
**9.3 PHARMACOKINETICS ... 57**
9.3.1 ANALYTICAL MEASUREMENTS ... 57

9.3.1.1 Summary of Substances to be Analyzed ..... 57
9.3.1.2 Time Points/Intervals for Sample Collection ..... 58
9.3.1.3 Sample Handling Procedures ..... 59
9.3.1.4 Appropriateness and Description of Analytical Method(s) ..... 59
9.3.1.5 Blood Collection for Pharmacokinetic Assessment ..... 60
9.3.2 PHARMACOKINETIC VARIABLES ..... 60
9.3.2.1 Description of the Pharmacokinetic Parameters ..... 60
9.3.2.2 Methods for Derivation of Pharmacokinetic Parameters ..... 60
9.3.2.3 Appropriateness of the Pharmacokinetic Parameters ..... 60

**9.4 SAFETY VARIABLES ..... 61**
9.4.1 DESCRIPTION OF THE SAFETY VARIABLES ..... 61
9.4.1.1 Laboratory Assessments ..... 61
9.4.1.2 Immunogenicity ..... 62
9.4.2 TIME POINTS/INTERVALS FOR MEASURING THE SAFETY VARIABLES ..... 62
9.4.3 APPROPRIATENESS AND DESCRIPTION OF MEASUREMENTS OF THE SAFETY VARIABLES ..... 65

**9.5 CHANGES IN LABORATORY PARAMETERS ..... 66**

**10 SAFETY ASPECTS ..... 67**
**10.1 ADVERSE EVENTS (AE) ..... 67**
**10.2 ADVERSE DRUG REACTIONS (ADR) AND UNEXPECTED ADVERSE DRUG REACTIONS ..... 67**
**10.3 SERIOUS ADVERSE EVENTS (SAE) AND SERIOUS ADVERSE DRUG REACTIONS (SAR) ..... 67**
**10.4 SUPECTED UNEXPECTED SERIOUS ADVERSE REACTION (SUSAR) ..... 68**
**10.5 ELICITING, DOCUMENTATION AND REPORTING OF ADVERSE EVENTS ..... 68**
**10.6 RATING OF ADVERSE EVENTS ..... 69**
**10.7 DOCUMENTATION AND REPORTING OF IMMEDIATELY REPORTABLE ADVERSE EVENTS ..... 70**
**10.8 WARNINGS AND PRECAUTIONS ..... 72**

**11 QUALITY ASSURANCE AND QUALITY CONTROL ..... 73**
**11.1 MONITORING ..... 73**
**11.2 AUDIT AND INSPECTIONS ..... 73**

**12 DOCUMENTATION, RECORD ACCESS, AND RECORD KEEPING ..... 74**
**12.1 DOCUMENTATION AT THE STUDY CENTER ..... 74**
**12.2 SUBJECTS' DATA AND DATA PROTECTION ..... 74**
**12.3 DATA MANAGEMENT/CODING ..... 74**
**12.4 RECORD KEEPING / ARCHIVING ..... 75**

**13 STATISTICAL EVALUATION ..... 75**
**13.1 STATISTICAL HYPOTHESES ..... 75**
**13.2 DETERMINATION OF SAMPLE SIZE ..... 75**

**13.3 STUDY SUBJECTS ........ 76**
13.3.1 STUDY POPULATIONS ........ 76
13.3.2 PROTOCOL DEVIATIONS ........ 76
13.3.3 DEMOGRAPHIC AND OTHER BASELINE CHARACTERISTICS ........ 76
13.3.4 DECODING OF RANDOMIZATION ........ 77
**13.4 EFFICACY EVALUATION ........ 77**
**13.5 PHARMACODYNAMIC EVALUATION ........ 77**
**13.6 PHARMACOKINETIC EVALUATION ........ 77**
13.6.1 STATISTICAL/ANALYTICAL ISSUES ........ 77
13.6.1.1 Summary of Pharmacokinetic Evaluations ........ 77
13.6.1.2 Evaluation of Steady-State ........ 77
13.6.1.3 Adjustments for Covariates ........ 78
13.6.1.4 Handling of Dropouts or Missing Data ........ 78
13.6.1.5 Interim Analyses and Data Monitoring ........ 78
13.6.1.6 Multicenter Studies ........ 78
13.6.1.7 Multiple Comparison/Multiplicity ........ 78
13.6.1.8 Examination of Subgroups ........ 78
13.6.2 TABULATION OF INDIVIDUAL DATA ........ 78
13.6.3 DRUG DOSE, DRUG CONCENTRATION, AND RELATIONSHIPS TO PHARMACOKINETIC DATA ........ 79
13.6.4 BY-SUBJECT DISPLAYS ........ 79
**13.7 SAFETY EVALUATION ........ 79**
13.7.1 STATISTICAL/ANALYTICAL ISSUES ........ 79
13.7.1.1 Adjustments for Covariates ........ 79
13.7.1.2 Handling of Dropouts or Missing Data ........ 79
13.7.1.3 Interim Analyses and Data Monitoring ........ 79
13.7.1.4 Multicenter Studies ........ 80
13.7.2 EXTENT OF EXPOSURE ........ 80
13.7.3 ADVERSE EVENTS (AES) ........ 80
13.7.3.1 Display of Adverse Events ........ 80
13.7.3.2 Analysis of Adverse Events ........ 80
13.7.3.3 Listing of Adverse Events by Subject ........ 80
13.7.4 DEATHS, OTHER SERIOUS ADVERSE EVENTS, AND OTHER SIGNIFICANT ADVERSE EVENTS ........ 80
13.7.5 CLINICAL LABORATORY EVALUATION ........ 81
13.7.6 VITAL SIGNS, PHYSICAL FINDINGS, AND OTHER OBSERVATIONS RELATED TO SAFETY ........ 81
**14 ADMINISTRATIVE PROCEDURES ........ 81**
**14.1 AMENDMENTS TO THE PROTOCOL ........ 81**
**14.2 CONFIDENTIALITY AND PUBLICATION ........ 82**
**14.3 REGISTRATION OF STUDY AND INVESTIGATORS ........ 82**
**14.4 INSURANCE, CONTRACTS, FINANCES ........ 82**
**15 REPORTING ........ 83**
**16 REFERENCE LIST ........ 84**

# LIST OF TABLES

Table 1: Flow Chart – Stage 1 .......... 12
Table 2: Flow Chart – Stage 2 .......... 13
Table 3: Stage 1 dose cohorts .......... 48
Table 4: Stage 2 dose cohorts .......... 48
Table 5: Pharmacodynamic sample collection .......... 53
Table 6: Timing of blood sampling for Hepcidin assessment .......... 54
Table 7: Timing of blood sampling for pharmacodynamic assessment – fast response kinetic .......... 55
Table 8: Timing of blood sampling for pharmacodynamics assessment during hospitalization – slow response kinetic .......... 55
Table 9: Timing of blood sampling for exploratory biomarker assessment .......... 56
Table 10: Timing of blood sampling for Anticalin PRS-080#022-DP pharmacokinetics .......... 58
Table 11: Time deviations requiring CRF comment .......... 58
Table 12: Timing of vital signs, respiratory rate and ECG assessments during hospitalization .......... 62
Table 13: Timing of temperature assessments during hospitalization .......... 63
Table 14: Timing of laboratory assessments during hospitalization .......... 63
Table 15: Timing of adverse event questioning and concomitant medication during hospitalization .......... 64
Table 16: Timing of local tolerability assessments during hospitalization .......... 65
Table 17: Injection site reaction score .......... 65

# 1 INVESTIGATORS AND STUDY-ADMINISTRATIVE STRUCTURE

## 1.1 REQUIREMENTS CONCERNING THE INVESTIGATOR(S) AND STUDY CENTER(S)

Nuvisan GmbH began operation under the name L.A.B. GmbH & Co as a privately owned contract research institute in 1979 and has been performing clinical pharmacological and pharmacokinetic studies since that time. In January 1997, L.A.B. became a wholly owned subsidiary of AAI (Applied Analytical Industries, Inc), a pharmaceutical development company based in Wilmington, North Carolina. In March 2010, the company name "AAIPharma Deutschland GmbH & Co. KG" was changed to "Nuvisan Pharma Services GmbH & Co. KG". In June 2010 Nuvisan Pharma Services GmbH & Co KG was acquired from the company ADCURAM. In July 2010 under a transfer of operations, the name of the company was changed to Nuvisan GmbH.

Appropriateness of the Study Center:

The Clinical Center of Nuvisan GmbH (Clinical Pharmacology Phase I / IIa) comprises overnight facilities with full catering services including special diets as well as separate function rooms for drug administration, blood sampling, sample work up, ECG recording, interim storage of study medication as well as an "intensive care unit" with 3-lead ECG telemetry equipment for 18 subjects etc. and a screening examination unit. An in-house clinical laboratory unit is available providing the option of approximately 200 established clinical laboratory parameters. An emergency carriage including defibrillator is available at the intensive care unit. Transportable emergency equipment is available on the other floors. All equipment as required for measurement of vital signs, ECG recording, parenteral drug administration etc. is available and maintained according to the "Medizinprodukte Gesetz". If necessary, additional or special equipment is rented for the required time.

Qualifications of the Investigators and Team members:

Nuvisan team members conducting the study are medically trained physicians, research nurses and laboratory technicians in cooperation with non-medical team members.

Physicians, taking on medical responsibility for the studies as responsible investigator, or principal investigator, have to have at least 2 years of experience in the conduct of clinical trials as well as study nurses experienced in the preparation and performance of clinical trials, the majority of them for many years. They regularly undergo training in SOPs, GCP guidelines, newly implemented legislation etc. They are practically and theoretically trained in the treatment of medical emergencies.

Specialists on a specific field or medicinal specialty, e.g. anesthesiology, join the group of investigators if considered necessary for the study. Such specialists also serve as consultants.
The investigators are employed by Nuvisan GmbH and are fully financially compensated by their salary.

The study is financially covered by the sponsor on the basis of the Clinical Services Agreement as of the 17th day of May 2013 between sponsor and Nuvisan. The contract defines the responsibilities of the sponsor, Sponsor's QPPV and Monitors and Nuvisan (CRO) including Nuvisan's investigators.

The Analytical Research Center concentrates on the analysis of biological samples from absorption, distribution, metabolism, elimination as well as bioavailability (BA)/bioequivalence (BE) studies using state of the art high performance liquid chromatography (HPLC), liquid chromatography mass spectrometry (LCMS), gas chromatography mass spectrometry (GCMS), mass spectrometry (MS) and radioimmunoassay (RIA) techniques. More than 300 validated methods are available to the clients while other proprietary methodology has been developed jointly with pharmaceutical manufacturers.

For Nuvisan staff members' roles in the study please refer to Section 1.3.

The Principal Investigators involved in the clinical services described in this protocol agree to conduct the clinical study in compliance with the protocol agreed with by Pieris AG and which was approved by the Ethics Committee and the Competent Authority. Not less than the Principal Investigator and a sponsor's representative will sign the protocol (and protocol amendments) to confirm this agreement.

## 1.2 DOSE ESCALATING COMMITTEE (DEC)

A Dose Escalation Committee (DEC) will provide recommendations about stopping, modifying or continuing the trial. The DEC will meet for cohort safety review to provide dose escalation recommendations for both stages and will give recommendation for dose level and frequency for the MAD stage.

The committee will be composed of:

- the QPPV
- the Principal Investigator,
- the responsible Investigator
- a representative of the sponsor.

Details of the DEC's roles and responsibilities will be described in a safety review procedure manual.

## 1.3 ADMINISTRATIVE STRUCTURE

Principal Investigator

Dr. med. Werner Feuerer
Nuvisan GmbH
Wegenerstraße 13
89231 Neu-Ulm
Germany
E-mail: 264606.599146@kabelbw.de
Telephone number: 0170-2387547
Fax number: 0731/9840-355

Responsible Investigator

Michael Lissy, Physician
Nuvisan GmbH
Wegenerstraße 13
89231 Neu-Ulm
Germany
E-mail: Michael.Lissy@nuvisan.com
Telephone number: 0731/9840-125
Fax number: 0731/9840-355

Project Manager:

Gabriele Piskol
Nuvisan GmbH
Wegenerstraße 13
89231 Neu-Ulm
Germany
E-mail: Gabi.Piskol@nuvisan.com
Telephone number: 0731/9840-336
Fax number: 0731/9840-355

Sponsor's responsible medical expert (QPPV):

Dr. Edgar Fenzl
(Managing Director)
FGK Clinical Research GmbH
Heimeranstr. 35, 80339 Munich
Germany
E-Mail: Edgar.fenzl@fgk-cro.de
Telephone number: +49 (0) 89 893119-22
Fax number: +49 (0) 89 893 119-20

Monitor:

Dr. Zurab Koberidze
(Director Medical Safety)
FGK Clinical Research GmbH
Heimeranstr. 35
80339 Munich
Germany
E-Mail: zurab.koberidze@fgk-cro.de
Telephone number: +49 (0) 89 893119-133
Fax number: +49 (0) 89 893119-20

Statistician:

Dr. rer. pol. Dirk Lehnick
Director Biostatistics
Nuvisan GmbH
Wegenerstraße 13
89231 Neu-Ulm
Germany
E-mail: Dirk.Lehnick@nuvisan.com
Telephone number: 0731/9840-340
Fax number: 0731/9840-280

Responsible Bioanalyst:

Dr. Eddy Bruyns
Director Biopharmaceuticals Nuvisan GmbH
Wegenerstraße 13
89231 Neu-Ulm
Germany
E-mail: Eddy.Bruyns@nuvisan.com
Telephone number: 0731/9840-282
Fax number: 0731/9840-226

Responsible Bioanalyst: (for Hepcidin)

Dr. D.W. Swinkels
Professor of Experimental Clinical Chemistry
Universitair Medisch Centrum St Radboud
Laboratoriumgeneeskunde,
LGEM – sectie IJzercentrum
Huispost 830, route 830
Geert Grooteplein 10
6525 GA Nijmegen
The Netherlands
E-mail: D.Swinkels@labgk.umcn.nl
Telephone number: + 31 24 3618947
Fax number: + 31 24 35 44 1743

Clinical Laboratory:

Dr. rer. nat. Jutta Michael-Hepp
Director Clinical Laboratory
Nuvisan GmbH
Wegenerstraße 13
89231 Neu-Ulm
Germany
E-mail: Jutta.Hepp@nuvisan.com
Telephone number: +49 731/9840-230
Fax number: +49 731/9840-465

Quality Assurance Unit:

Sigrid Hopp
Director Quality Management, Europe
Nuvisan GmbH
Wegenerstraße 13
89231 Neu-Ulm
Germany
E-mail: Sigrid.Hopp@nuvisan.com
Telephone number: 0731/9840-298
Fax number: 0731/9840-280

## 1.4 REGULATIONS AND GUIDELINES

This study will be conducted in compliance with the Declaration of Helsinki in the valid version and the ICH Guideline of Good Clinical Practice (GCP)(1).
In addition, the EU Clinical Trial Directive, 2001/20/EC(2), the German Drug Law ('Arzneimittelgesetz' = AMG)(3), the GCP Ordinance (GCP-Verordnung)(4), the Professional Code for Physicians of Bavaria ('Berufsordnung für Ärzte Bayerns')(5) and the provisions of the data protection laws will be followed.

# 2 INTRODUCTION

## 2.1 BACKGROUND

### 2.1.1 ANEMIA OF CHRONIC DISEASE

Anemia is caused by iron deficiency and a subsequent impairment of hematopoiesis. It can result from insufficient uptake of iron, or from insufficient release of iron from the body's iron stores, or from the combination of both. Anemia of chronic disease (ACD) is a hypo-proliferative chronic anemia that develops in response to systemic illness or inflammation, and is commonly associated with inflammatory diseases and long term infections (including HIV/AIDs and hepatitis B or C), autoimmune disease (including Crohn's disease and rheumatoid arthritis), cancer (including lymphoma and Hodgkin's disease) and with chronic kidney disease. Patients with ACD sustain a relatively normal level of function at significantly reduced hemoglobin (Hb) levels, but will suffer from symptoms of hypoxia, including fatigue, headache, dizziness, tinnitus, syncope, and chest pain.
Treatment of ACD with oral iron supplements is ineffective, as intestinal iron adsorption is inhibited. The use of erythropoietin stimulating agents (ESAs) such as epoietin alfa is limited due to known side effects of ESAs, but also due to the need for adequate iron stores for ESAs to function effectively. Intravenous iron is being used increasingly. However, increases in the understanding of iron metabolism and its regulation, following the discovery of the hepatic hormone hepcidin, have brought the use of i.v. iron into question, and introduced a new target pathway for the treatment of ACD.

### 2.1.2 HEPCIDIN

Hepcidin has been identified as the systemic iron regulatory hormone. Mature hepcidin is a 25-amino acid peptide hormone synthesized primarily in hepatocytes. Hepcidin regulates the absorption of dietary iron across the duodenum, iron recycling from senescent erythrocytes, and the recovery of iron from storage in hepatocytes and macrophages, providing iron for the synthesis of heme and hemoglobin in bone marrow erythrocyte precursors (6). Hepcidin regulates the protein ferroportin, which acts as a transmembrane channel for the passage of cellular iron to plasma; binding of hepcidin to ferroportin causes its internalization and degradation, leading to a reduction of ferroportin on the membrane, and a decrease in movement of iron to plasma. The production of hepcidin is homeostatically regulated by plasma iron concentrations and iron stores (7) by means of a typical homeostatic negative-feedback loop, but its synthesis is also increased in infection and inflammation (8). This increase in hepcidin leads to the retention of iron in macrophages, reducing the amount of iron available for erythropoiesis, thereby leading to anemia. Thus, the hepcidin-ferroportin axis has become an attractive target for the therapeutic management of ACD, with numerous strategies for intervention available (9), and a number of agents are already in development.

### 2.1.3 PRS-080

PRS080#022-DP belongs to a new class of therapeutic proteins, Anticalins®, which are artificial lipocalins, developed by Pieris AG (Pieris) and based on a proprietary lipocalin platform. Lipocalins are low molecular weight proteins that are abundantly expressed in human tissues and body fluids. Anticalins are homologous to naturally occurring lipocalins (e.g., tear lipocalin and neutrophil gelatinase-associated lipocalin (NGAL)) and can be generated against a variety of targets using mutation and selection processes. PRS080#022-DP is the second Anticalin entering clinical development. It is composed of the protein moiety PRS-080 which is derived from the human lipocalin, NGAL (Lcn2, Uniprot # P80188) and a polyethylene glycol (PEG30) moiety to extend the half-life of the protein in plasma. The drug substance PRS080#022 is principally identical to the drug product since it is only sterile filtered and filled to become the drug product PRS080#022-DP.
PRS-080 is directed against human hepcidin (HAMP, Uniprot # P81172) protein and was selected via phage display technology. Hepcidin is a central regulator of iron homeostasis and functions by binding to and initiating the internalization and degradation of ferroportin, the only known cellular iron exporter. Many disorders of iron imbalance can be attributed to aberrant hepcidin production (Andrews 2012). Elevated plasma levels of hepcidin are regarded as the root cause of the hypoferremia and iron-restricted erythropoiesis seen in ACD. PRS-080 is obtained by bacterial expression in E. coli. The chosen expression system was optimized for efficient soluble expression in the cytoplasm. After cell harvest, PRS-080 is purified via two chromatographic steps. The highly pure intermediate obtained after these 2 steps is subject to PEGylation. After a further chromatographic removal of free PEG the final API, PRS-080#022, is obtained in PBS pH 6.5. A concentration of 8.1mg/mL protein content is adjusted. No excipients are added. The final product PRS-080#022-DP is stored at -20 °C +/- 5°C.
PRS-080#022-DP is a sterile solution to be administered by slow intravenous infusion over a time period of up to 2 hours.

### 2.1.4 NON-CLINICAL DATA WITH PRS-080[1]

*In-vitro* kinetic assays using surface plasmon resonance have demonstrated picomolar affinity and high specificity for the binding of PRS-080#012 to human hepcidin. A functional assay conducted in T-Rex-293 cells transfected with the human FPN-GFP-coding sequence showed that PRS-080 was capable of inhibiting the internalization and degradation of the FNP-GFP fusion protein with an $IC_{50}$ of ~14 nM.
Proof of concept for PRS-080 has been obtained in *in vivo* studies conducted in cynomolgus monkeys, wherein 4 mg/kg PRS-080#021 produced a robust, transient and reversible increase in total iron levels from ~27 µM at baseline to 52 µM after 24 hours, with iron responses detectable at doses as low as 0.4 mg/kg (minimal effective dose). The $C_{max}$ of the iron response was capped starting at 3 mg/kg and did not increase at higher doses of up to

---

[1] Please note: PRS-080#022 is a PEGylated protein. The dosage in non-clinical testing was always calculated on the basis of the whole product (protein + PEG moiety). All data in the non-clinical section of the investigator brochure (IB) relate to the whole product (strength of product 20mg/mL). In view of the "Guideline on the description of the composition of PEGylated (conjugated) proteins in the SPC" the dosage for administration to humans is provided on the basis of the protein content only, which is by a factor of 2.46 (rounded to 2.5) lower than the content of the whole product. Therefore, for this protocol the non-clinical dose levels and concentrations stated in the IB have been converted to protein concentrations (see Sections 2.1.4, 2.3 and 4.4) to link them directly to the proposed dose levels for Stage 1 of the first in human study.

150 mg/kg also during repeat dose administrations. PK properties were assessed in three preclinical species and used for allometric scaling to predict a human half-life of 50-60 hours.
An MTD study was performed in Cynomolgus monkeys to establish the tolerability of PRS-080#022 in the relevant toxicology species, to determine the toxicokinetics and toxicity of the test article following intravenous (30-minute infusion) administration. This single-dose phase with dose levels ranging from 8- 60 mg/kg was followed by a repeat dose phase at 60 mg/kg with four administrations every second day. No clinical signs were observed, other than bruising (inguinal and at infusion sites), and there were no macroscopic findings. Microscopic examination showed minimal to moderate tubular vacuolation in the kidney, and minor levels of hemosiderin in the liver. There were minor levels of fasciitis and periphlebitis consistent with venipuncture. Overall, PRS-080 was well tolerated, with a No-Observed-Adverse-Effect Level (NOAEL) of 60 mg/kg/dose for the i.v. route. In a 4-week GLP repeat-dose study in cynomolgus monkeys, animals were treated every other day at 8, 24, or 48 mg/kg, i.v. There were no clinical observations, and clinical chemistry and hematology was unremarkable. Drug-specific anti-drug antibody (ADA) responses were detected in the majority of animals, but these antibodies did not neutralize the pharmacodynamic activity of PRS-080. Microscopic examination showed hemosiderin in the periportal hepatocytes, consistent with the pharmacodynamic activity of PRS-080, and additional findings that were consistent with PEG administration. The NOAEL in this study was determined as 48 mg/kg given every other day.

## 2.2 RATIONALE

Anticalins are engineered human proteins that are able to bind either to proteins or to small molecules. The Anticalin PRS-080#022-DP to be investigated in this study is directed against hepcidin and is supposed to treat patients with anemia of chronic disease. This phase I FIH study shall investigate safety and pharmacokinetics in healthy human volunteers.
The design has been chosen as a classical dose escalation design as required in this type of trial. Based on the pre-clinical toxicological and safety pharmacology studies, the first dose to administer was considered to be justified.

## 2.3 JUSTIFICATION FOR DOSES TO BE EVALUATED

According to the EU guideline “Strategies to identify and mitigate risks for first-in-human clinical trials with investigational medicinal products”, PRS-080 is not classified as a high risk product, based on its mode of action, the nature of the target and the relevance of the animal models used during its non-clinical development. PRS-080 is a protein with an 8-stranded beta-barrel structure, and has a known mechanism of action, exerting its effect by functional antagonism of hepcidin, a peptide which acts to regulate the transmembrane channel ferroportin; as such, administration of this IMP is not expected to induce a biological cascade or cytokine release. Safety studies were conducted in cynomolgus monkeys which, as a non-human primate, can be considered as a relevant species, with non-clinical data demonstrating comparable binding affinity of PRS-080 to human and cynomolgus hepcidin, which was orders of magnitude greater than to that of rat, mouse, rabbit and dog. Findings of the toxicology study in this species were unremarkable (see Section 2.1.4). In addition, the pharmacodynamic effects of PRS-080 were demonstrated to be transient in this species. Previous experience of Anticalins in human subjects, whilst

limited to one trial with the product Angiocal®, showed this Anticalin to be overall safe and well tolerated; the principal toxicity was infusion reactions, which were shown to be eliminated by using a slow infusion rate (120 min duration). Based on the absence of risk factors that would warrant adoption of a “Minimal Anticipated Biological Effect Level” (MABEL), use of the “No Observed Adverse Effect Level” (NOAEL) approach is considered to be justified for this first-in-man study with PRS-080. The NOAEL was determined as 48 mg/kg given every other day (see Section 8.4).

In the Stage 1, dosing can only commence for the next cohort after satisfactory review of the safety data from the proceeding cohort by the Dose Escalation Committee (DEC). Safety data (adverse events, vital signs, laboratory safety tests and objective test measures) from the 72-hour treatment period, of all subjects in the cohort, must be reviewed by the Investigators and DEC as part of the review of the safety data. Approval by the Investigator and the DEC will be required to escalate the dose.

At the conclusion of the meeting after the last dose of the SAD stage, the DEC will provide recommendation to continue the trial, corresponding next dosage level and dosing frequency for the MAD stage.

In the Stage 2, dosing can only commence for the next cohort after satisfactory review of the safety data from the proceeding cohort by the Dose Escalation Committee (DEC). Safety data (adverse events, vital signs, laboratory safety tests and objective test measures) from the last treatment period 72h, from all subjects in the cohort, must be reviewed by the DEC as part of the review of the safety data. Approval by the Investigators and the DEC will be required to escalate the dose.

# 3 STUDY OBJECTIVES

**Primary Objectives**

The primary objectives of the study are:

- to determine the safety and maximum tolerated dose (MTD) of single and repeated intravenous infusion with PRS-080#022-DP
- to collect data concerning pharmacokinetics of PRS-080#022-DP upon intravenous dose
- to collect data concerning clinical safety and tolerability
- to collect data on immunogenicity

**Secondary Objectives**

The secondary objectives of the study are:

- to evaluate pharmacodynamic parameters including dose-dependent iron response
- to evaluate dose-dependent target engagement (hepcidin)

The results are to define safe and potentially effective dose levels for a phase IIa study in patients with anemia for therapeutic proof of concept.

<u>Primary variables to be studied in detail are:</u>

- Safety: AEs, vital signs (BP, HR), ECG, laboratory parameters including hs CRP after single and multiple dose administration.

<u>Secondary variables to be studied are:</u>

- Anticalin Pharmacokinetics: $C_{max}$, $t_{max}$, $AUC_{0-t}$, $AUC_{0-\infty}$, $AUC_{0-\tau}$, $t_{1/2}$, MRT after single and multiple ascending dose administration.
- ADA responses (immunogenicity) after single and multiple ascending dose administration
- Target engagement: serum hepcidin after single and multiple ascending dose administration
- Pharmacodynamics: iron response markers including but not limited to total iron, transferrin saturation, Hb, reticulocytes, reticulocyte Hb, after single and multiple ascending dose administration.

# 4 ETHICS

## 4.1 INDEPENDENT ETHICS COMMITTEE (IEC) AND COMPETENT AUTHORITY (CA)

Before the start of the study the sponsor or authorized applicant will apply for approval for the performance of the study at the Competent Authority (Federal Institute for Drugs and Medical Products (BfArM)). Nuvisan GmbH will apply for approval for the performance of the study at the Ethics Committee of the Bavarian Chamber of Physicians which is constituted according to local law. All documents required by the Ethics Committee and by the Competent Authority will be submitted.
Any notification / submission has to be dated and to contain sufficient information to identify the respective protocol.

The study will only be started after receipt of the written approval of the Ethics Committee of the Bavarian Chamber of Physicians and the Competent Authority.

The Principal Investigator and the sponsor are responsible for maintaining the approval documents in the study documentation files.
The Principal Investigator or the sponsor will report promptly to the Ethics Committee new information that may adversely affect the safety of the subjects or the conduct of the trial.

The sponsor (or authorized applicant), or Nuvisan GmbH on behalf of the sponsor, should submit a written report about the safety of the subjects as well as a list of occurred suspected serious adverse drug reactions caused by the investigational medicinal product of the clinical study to the Ethics Committee and the Competent Authority annually, or more frequently if requested by the Ethics Committee or the Competent Authority.

A declaration of the end of trial should be forwarded by the sponsor (or authorized applicant), or Nuvisan GmbH on behalf of the sponsor, to the Competent Authority and to the Ethics Committee within 90 days after the study has been completed or in the event of premature termination of the study within 15 days.

The sponsor (or authorized applicant) or Nuvisan GmbH on behalf of the sponsor should provide a summary of the clinical study report to the Ethics Committee and Competent Authority within 1 year after completion of the study.

The reporting to the IEC and the Competent Authority is clearly defined in the Quality Agreement (QAg) and responsibility list for clinical study. Both are part of the clinical service agreement between Sponsor and Nuvisan.

## 4.2 SUBJECT INFORMATION AND CONSENT

Prior to the start of screening examinations, the written informed consent form must be signed and personally dated by the subject (or the legally acceptable representative) and by the investigator who conducted the informed consent discussion.
In obtaining and documenting informed consent, the investigators must comply with the applicable regulatory requirement(s), and must adhere to Good Clinical Practice (GCP) and to the ethical principles that have their origin in the Declaration of Helsinki.
The investigator must fully inform the subject of all pertinent aspects of the trial including the written information approved/favorably assessed by the Ethics Committee. Subjects should not be screened or treated until the subject has signed an approved informed consent form written in a language that is understandable to the subject.
The subjects should be informed of the possibility to withdraw consent without giving any reason. The subject information should include a statement that the consequence of the subject's withdrawal of consent will be that no new information will be collected from the subject and added to existing data or a database. The investigators should maintain a log of all subjects who sign the informed consent form and indicate if the subject received study drug or, if not, the reason why. One original of the signed and dated informed consent form will be provided to the subject. A second original will be retained in the investigator trial master file.

## 4.3 GENERAL SAFETY

The study participants will be carefully screened prior to study enrolment for their health status and suitability. Inclusion and exclusion criteria have been chosen appropriately to minimize possible risks for subjects participating in this trial. During the study the subjects will be closely monitored by a broad safety battery, including adverse event (AE) reporting, physical examination if required, clinical chemistry examination, hematology, vital signs measurements, electrocardiogram (ECG), and urinalysis. After study drug administration the subjects will be under close medical surveillance; a specialist in anesthesiology will be available during and for at least 2 hours after drug administration in order to treat infusion related reactions and a physician will be available at the trial site until 24 hour after drug administration[2]. The duration of hospitalization and the medical surveillance are considered adequate to ensure safety of the subjects. The clinic of Nuvisan GmbH is enclosed in the public emergency system of the city of Neu-Ulm/Ulm; an emergency physician can be on site within 10 minutes and provide a transport to the indicated hospital e.g. intensive care unit. In most cases, for NUVISAN this is either the University Hospital of Ulm or the Military Hospital of Ulm or the local hospital of Neu-Ulm (Donauklinik); the nature of the emergency is crucial for the selection of the hospital. Subjects will be discharged from the study unit if there are no withstanding safety concerns. In addition to this, subjects will receive an Emergency Card with information on their status and applied drug on leaving the study unit.

[2] For each first administration or as required.

General precautions include those related to protein-induced anaphylactic, infusion-related reactions of single individuals, which require emergency interventions by a physician including application of high dose corticosteroids, anti-histaminic drugs, volume resuscitation and catecholamine therapy as well as $O_2$-insufflation or general resuscitation in rare cases.

## 4.4 BENEFIT-RISK EVALUATION

There are no perceived direct benefits to healthy volunteers participating in this trial. However, thorough medical check-ups may be seen as an advantage.
Evaluation of the safety of PRS-080#022 in non-human primates demonstrated a favourable toxicity profile (10). In a 4-week repeat-dose study of doses from 8-48 mg/kg i.v. every other day, there were no clinical signs, no effects on organ weight or macroscopic observations, and no safety pharmacology findings; clinical chemistry and hematology results were unremarkable. Microscopic findings were reported (see Section 2.1.4), predominantly the presence of hemosiderin in the periportal hepatocytes (a pharmacodynamic effect) and additional effects consistent with PEG administration. These limited changes were considered to be sufficiently benign that the highest dose studied (48 mg/kg) was determined as the NOAEL.
Whilst PRS-080#022 has not yet been administered to humans, another Anticalin product, albeit one based on a modified lipocalin-1 backbone (rather than the lipocalin-2 backbone seen in PRS-080), has been studied in a phase I safety, tolerability and PK study in patients with solid tumors. This agent, an Anticalin targeting VEGF-A, called Angiocal, was demonstrated to be safe and well tolerated overall, with toxicity limited to dose-dependent infusion reactions, including fever and chills. Such reactions, although not prevented by prophylactic treatment with clemastin, ranitidine and fortecortin, were successfully eliminated by extending the infusion time from 20 min to 120 min (11). In addition, the strategy of targeting hepcidin has been shown to be safe and well tolerated in a Phase 1 study with the anti-hepcidin spiegelmer Nox-H94 in healthy volunteers, where headache and fatigue were the only treatment-related adverse events reported more than once; there were no SAEs (12).
Based on the experimental animal studies that were carried out, investigation of the safety and tolerance of the PRS-080#022 showed no special dangers for humans. Therefore, based on the safety profile of the PRS-080#022 the risks in participating in the trial are considered acceptable. However, they include the usual risks of participating in clinical trials, which are related to possible allergic reactions, blood drawing via venipuncture or indwelling catheter. The safety of the subjects will be observed during all study phases. Before the first drug administration, the participants of the subsequent groups will be informed about the adverse effects that occurred in the previous group(s).
Medical progress is based on research which ultimately must rest in part on experimentation involving humans. Eligible subjects may consider participation in this clinical trial because they want to contribute to the advancement of medical knowledge. Still, considerations related to the well-being of the individual subjects enrolled into this clinical study must take precedence over the interests of science and society. Based on available information and the design of the study, the sponsor and the Principal investigator consider the trial to be ethically acceptable. The duration of hospitalization and the medical surveillance are considered adequate to ensure safety of the subjects.

# 5 OVERALL STUDY DESIGN AND PLAN

## 5.1 OVERALL STUDY DESIGN

This “umbrella” protocol describes the planned conduct of a SAD and MAD stage to be performed with the novel anti-hepcidin Anticalin PRS-080#022-DP. These two study stages are to be conducted sequentially.

### 5.1.1 STAGE 1

The first stage is entitled “A First-in-Human (FIH), Randomized, Dose-Escalation, Double-Blind, Placebo-Controlled single ascending dose study (SAD) in healthy volunteers to establish safety, lack of immunogenicity, tolerability, pharmacokinetic parameters, dose-dependent target engagement and pharmacodynamic effects”.
The study will enroll 8 subjects (6 verum, 2 placebo) per cohort, up to a maximum tolerated dose, defined by the stopping rules (see Section 7.3.1). Six (6) dose levels are anticipated (see Section 8.5).
The decision to dose subsequent sub-groups at the same dose level will be based on an interim analysis of clinical safety and safety laboratory data 24 hours after start of infusion.
Drug administration will start sequentially between each individual subject with an interval of 150 minutes after start of infusion (except pilot group of each cohort). The study drug will be administered as a 120 min i.v. infusion on Day 1. The study drug will be added to an infusion bag resulting in a total volume of 250 mL of a clinical grade infusion solution and administered with a constant volume rate of 125 mL per hour using an Infusomat.
The study performance of the cohorts is staggered by approximately 2 week between the cohorts.
The decision to escalate the dose will be made by a dose escalation committee (DEC) based on an interim analysis of clinical safety and safety laboratory data 24 hours and 72 hours after the start of infusion.
Subjects will be hospitalized from 24 hours before until 72 hours after start of infusion.
The study will involve the following assessments:

- Safety and tolerability will be assessed by recording of adverse events, vital signs, 12-lead ECGs, respiration rate, and local tolerability assessment at the time points indicated in the Flow Chart in Table 1.
- Body temperature (ear) will be measured at the time points indicated in the Flow Chart in Table 1.
- Blood sampling for Anticalin pharmacokinetics will comprise 240 hours (assuming $T_{max}$ of 5 min at the end of infusion and $t_{1/2}$ of 50 hours), see Flow Chart in Table 1. (14° samples)
- Urine samples will be collected 0h (pre-dose), from 0h (pre-dose) to 24h and at 48 and 72 hours after start of infusion.
- Blood sampling for target engagement (Hepcidin) in serum will comprise 240 hours after start of infusion (same time points as for PK plus samples at screening, at -24 h [i.e. at admission], and on Day°28; this should be taken in the morning similar to the pre-dose sample due to the diurnal rhythm of hepcidin). (17 samples)

- Urine and blood sampling for clinical safety laboratory including hs CRP will comprise assessment at screening, on Day -1, and at 24, 72, 120 and 240 hours after start of infusion. (6 samples)
- Blood sampling for pharmacodynamic markers with a fast response kinetic (i.e. iron, transferrin saturation, and ferritin) in serum will be performed at screening, at -24 h (i.e. on admission) and then regularly over 240 hours after start of infusion, see Flow Chart in Table 1. (14 samples)
- Blood sampling for pharmacodynamic markers with a slow response kinetic (i.e. Hb, reticulocytes, reticulocyte Hb) in whole blood at screening and regularly over 240 hours after start of infusion, see Flow Chart in Table 1. (9 samples)
- Blood samples will be taken and stored for analysis of protein-based biomarkers e.g. soluble transferrin receptor or cytokines in serum and plasma at selected PK time points (pre-dose, 2 hours, 6 hours and 24 hours and 72 hours) if warranted by the results of clinical safety laboratory, hepcidin or pharmacodynamic marker analysis. (5 samples)
- Blood sampling for ADA responses (immunogenicity) pre-dose and at an appropriate time (4 weeks) after dosing. (2 samples)

Following each dose level, an interim analysis based on safety and tolerability results up to 72 hours after dosing (including AEs, safety laboratory, vital signs) will be performed on which the next dose increment will be decided on by the DEC.

<u>SAD/MAD transition:</u>
Following the completion of the SAD part including assessments of safety and tolerability, Anticalin PK, PD responses and ADA responses, an unblinded evaluation will be performed to support the decision process of the DEC in terms of the MAD stage. All needed steps (including monitoring, data validation, data review) will be performed in order to close the database after Stage 1 prior to unblinding. Prior to entering into Stage 2 (the MAD stage), at least a selection of evaluations based upon the SAD part should be completed which will help the DEC and the sponsor to determine the dose levels and corresponding dosing schemes for the MAD stage.

### 5.1.2 STAGE 2

Directly after completion and evaluation of the SAD, a repeat dose study stage will follow entitled “Randomized, Dose-Escalation, Double-Blind, Placebo-Controlled multiple ascending dose study (MAD) in healthy volunteers to establish safety, lack of immunogenicity, tolerability, pharmacokinetic parameters, dose-dependent target engagement and pharmacodynamic effects”.
The MAD study stage, considered to be “Stage 2”, will be a randomized, dose-escalation, double-blind, placebo-controlled repeat dose study conducted in healthy volunteers.
This multiple rising dose study will enroll 2 cohorts of 12 subjects each (9 verum, 3 placebo). The dose levels will be limited to 1 to 2 dose levels below the MTD dose of the SAD study or, if no MTD was observed, 1 dose level below the highest dose group tested.
The study drug will be administered over 13 days (7 times, every second day) as a 120-min i.v. infusion on Days 1 to 13, to reach steady state. The dosing frequency may be adjusted depending on the PK/PD relationship established during the SAD phase (human half-life, target inhibition and iron response).

The study performance of the cohorts is staggered by at least 1 week between the last dose of the previous cohort and the first dose of the next cohort.
The study will involve the following assessments:

- Safety and tolerability will be assessed by recording of adverse events, vital signs, 12-lead ECGs, respiration rate and local tolerability assessment at the time points indicated in the Flow Chart in Table 2.
- Body temperature (ear) will be measured at the time points indicated in the Flow Chart in Table 2.
- Laboratory safety tests will be assessed at the time point indicated in the Flow Chart in Table 2.
- Blood sampling time points to measure Anticalin PK from pre-dose Day 1 over 240 hours from the start of the last infusion are described in Table 2. (33 samples)
- Pharmacodynamic markers with a fast response kinetic (i.e. iron and transferrin saturation) will be assessed at screening, at -24 h (i.e. on admission), pre-dose on Day 1, over 36 hours from the start of the first infusion, then prior to dosing on subsequent treatment days, and over 240 hours from the start of the last infusion and Day 41, see Flow Chart in Table 2. (26 samples)
- Blood sampling will be performed to measure pharmacodynamic markers with a slow response kinetic at screening, from pre-dose Day 1 (start of the first infusion), over 36 hours from the start of the first infusion, prior to dosing on subsequent treatment days, and over 240 hours from the start of the last infusion and Day 41 (see Flow Chart in Table 2). (16 samples)
- Blood samples to assess target engagement (hepcidin) will be collected at screening, at -24 h (i.e. on admission), from pre-dose Day 1 (start of the first infusion), over 36 hours from the start of the first infusion, with additional sampling on subsequent treatment days, and over 240 hours from the start of the last infusion and Day 41, see Flow Chart in Table 2. (36 samples)
- Blood sampling for assessment of exploratory biomarkers will be collected over 24 hours from the start of the first infusion, with additional sampling on subsequent treatment days, and over 72 hours from the start of the last infusion (see Flow Chart in Table 2, and Section 9.2.2). (14 samples)
- Blood sampling to measure potential ADA after drug wash out (pre-dose and 4 weeks after last dose). (2 samples)

## 5.2 OVERALL STUDY PLAN

### 5.2.1 SCREENING EXAMINATION

In each of the two study stages described in this umbrella protocol, all volunteer subjects will undergo an entry examination to evaluate their health status. This examination will be conducted not more than twenty-one (21) days prior to the planned first drug administration. Only subjects meeting the inclusion and exclusion criteria will be admitted to the study.
During the screening examination, the subjects are identified by a 5-digit Nuvisan subject number. This number is assigned to a subject for all studies he has participated in at Nuvisan GmbH.

Measures to prevent study participation within a time span defined by a preceding study participation and to avoid participation in a study in parallel to another study:

Subjects are not allowed to have participated in a clinical trial within the last thirty (30) days prior to the planned first drug administration or during this trial. There is a notification on the subject’s card as well as in the electronic volunteer data base.
In addition, before inclusion into the study all subjects are reported to a central checking organization (VIP Check).

This screening examination will consist of the following:

- Medical history, including collection of demographic data
- Complete physical examination: including vital signs (blood pressure, pulse rate), respiratory rate, review of systems (eyes, ears, nose and throat [EENT], cardiac, peripheral vascular, pulmonary, musculoskeletal, neurologic, abdominal, lymphatic, dermatologic), height, weight, and BMI
- Assessment of compliance with inclusion/exclusion criteria
- Assessment of body temperature (ear)
- ECG (12-lead)
- Evaluation of laboratory results
- Blood sampling for pharmacodynamic markers (iron status)
- Blood sampling for hepcidin assessment
- Laboratory tests, to include hematology, biochemistry, coagulation, serology, urinalysis, and exclusion tests (see Section 9.4.1.1 for details)
- Alcohol breath test
- Drug screening (for drugs of abuse)

### 5.2.2 STUDY PERFORMANCE

The subjects willing to participate in the study will only be included when all screening examination procedures have demonstrated that all inclusion criteria and none of the exclusion criteria apply. They will be assigned a random number within the study. For detailed information about the procedure of assigning random numbers to subjects, please refer to Section 8.3.

#### 5.2.2.1 Duration of Confinement Periods

In each of the 2 study stages described in this protocol, subjects will be hospitalized the day before drug administration (-24 hr). The next morning (study Day 1), the investigational drug will be administered. For details about drug administration, please refer to Section 8. Subjects will be discharged from the Center for Human Pharmacology according to the study they participated in; subjects taking part in Stage 1 (SAD) will be discharged 72 h after initiating study treatment; subjects in the Stage 2 study (MAD) will be discharged 72 h after the start of the last study drug infusion (Day 16); All following activities will be performed on an ambulatory basis.

Dietary Regimen:
On Day 1 SAD and Days 1 and 13 MAD, the subjects will be fasting from at least 10 hours before until 4 hours after drug administration, with the exception of mineral water which may be consumed ad lib.
Water is allowed as desired. On admission to the clinic, the subjects will receive a bottle containing 1.5 liters of water. They will be asked to stay well hydrated during their stay in the clinic.
Four (4) hours after infusion start a lunch will be served, after the completion of scheduled assessments, and an afternoon snack at 7 hours after infusion start. Dinner will be served approximately 10 hours after infusion start and an evening snack at 12 hours after infusion start.
On the other dosing study days, the subjects will be fasting from at least 10 hours before drug administration, with the exception of mineral water which may be consumed ad lib. A light breakfast will be served about two hours after start of infusion and other meals will be served at a convenient time.
On the other study days meals will be served at a convenient time. Nuvisan standard menus will be used for all subjects while resident at the study site.
Subjects will be instructed to abstain from any xanthine-containing food or beverages (e.g. chocolates, tea, coffee or cola drinks), from grapefruit or its products, quinine containing beverages or food (bitter lemon, tonic water), poppy seed-containing beverages or food, and from alcohol and alcoholic products for 24 hours prior to first dose administration and throughout their stay at the study site.
Subjects will be instructed to abstain for 12 hours prior to any ambulatory study visit from:

- Caffeine-containing beverages or food (tea, coffee, cola, chocolate, etc.)
- Quinine-containing beverages or food (bitter lemon, tonic water)
- Grapefruit juice (sweet or sour)
- Poppy seed-containing beverages or food

Strenuous physical exercise is not allowed within 72 hours prior to first dose administration and throughout the subjects' stay at the study site.

### 5.2.2.2 Day -1

Subjects will be hospitalized on Day -1, approximately 24 h prior to the initiation of study drug administration. Subjects will be questioned regarding any changes in their health or any violation of protocol compliance since the last assessment. If there is any doubt regarding a subject's health status, the subject will not be dosed. Subjects testing positive for alcohol (breath test) or drugs (urine test) will be excluded from participation in the trial.
The following assessments will be performed for subjects in each of the two studies:

- Urine drug screen and an alcohol breath test will be performed
- Body weight
- Safety laboratory assessments
- Adverse events and concomitant medication
- Blood sampling for hepcidin
- Blood sampling for pharmacodynamics (i.e. fast response kinetic, iron, transferrin saturation and ferritin)

#### 5.2.2.3 Treatment Phase – Pre-Dose

For subjects enrolled in each of the studies described in this protocol, the following assessments are to be performed prior to the initiation of study drug administration; the time at which administration starts will be designated as time 0 h.

- Adverse event questioning
- Concomitant medication
- Vital signs (blood pressure and pulse rate)
- Body temperature (ear)
- Respiratory rate
- 12-Lead ECG
- Local tolerability
- Biochemistry
- Hematology
- Coagulation
- Urinalysis
- Blood sampling for Anticalin pharmacokinetics
- Blood sampling for hepcidin
- Blood sampling for pharmacodynamics (fast response kinetic, i.e. iron, transferrin saturation and ferritin)
- Blood sampling for pharmacodynamics (slow response kinetic, i.e. Hb, reticulocytes, reticulocyte Hb)
- Blood samples for exploratory biomarker analysis
- Pre-dose urine sample collection (Stage 1 only)
- Initiation of pooled urine sample collection for the period 0 h – 24 h (Stage 1 only)
- Blood sampling for ADA

Following completion of the pre-dose assessments, administration of study drug will be initiated (see Section 8.1).

#### 5.2.2.4 Treatment Phase – Post-Dose

In each of the two study stages described in this protocol, the following assessments will be performed at regular intervals following the initiation of study drug administration (including some assessments to be performed during the study drug infusion) during the subject's period of confinement to the study center.

- Adverse event questioning and concomitant medication
- Vital signs, ECG, and respiratory rate
- Body temperature (ear)
- Local tolerability
- Laboratory assessments (including biochemistry, hematology, coagulation and urinalysis)
- Blood sampling for Anticalin pharmacokinetics
- Blood sampling for hepcidin
- Blood sampling for pharmacodynamic assessment – fast components
- Blood sampling for pharmacodynamic assessment – slow components
- Exploratory biomarker analysis

The timing of these assessments, for each of the two study stages, is presented in full in Section 9.2.2 (for pharmacodynamic variables), Section 9.3.1.2 (for pharmacokinetic sampling) and Section 9.4.2 (for safety assessments).
All assessments scheduled for conduct at 72 h post-dose are to be performed prior to the subject's discharge from the study center.

#### 5.2.2.5 Treatment Phase – Ambulatory Visits

For each of the two study stages, an ambulatory visit will be conducted 5 days after administration of the last study drug. In Stage 1, this visit will take place on Day 6, 120 h post-dose. For those subjects enrolled in the Stage 2 study (MAD), this visit will be conducted 5 days after the subject's last study drug administration, taking place on Day 18.
During this visit, the following assessments will be conducted:

- Adverse event questioning
- Concomitant medication questioning
- Vital signs (blood pressure and pulse rate)
- Body temperature (ear)
- Respiratory rate
- 12-Lead ECG
- Local tolerability (Stage 1 [SAD] and Stage 3 [bioavailability] only)
- Biochemistry
- Hematology
- Coagulation
- Urinalysis
- Blood sampling for Anticalin pharmacokinetics
- Blood sampling for hepcidin
- Blood sampling for pharmacodynamics (fast response kinetic, i.e. iron and transferrin saturation)
- Blood sampling for pharmacodynamics (slow response kinetic, i.e. Hb, reticulocytes, reticulocyte Hb, and ferritin)

### 5.2.3 END OF STUDY EXAMINATION

In each of the two study stages described in this protocol, a follow-up examination will be performed 240 hr after the last dosing. This will be an ambulatory visit.

This examination will consist of:

- Complete physical examination: including review of systems (eyes, ears, nose and throat [EENT], cardiac, peripheral vascular, pulmonary, musculoskeletal, neurologic, abdominal, lymphatic, dermatologic)
- Adverse event questioning
- Concomitant medication questioning
- Vital signs (blood pressure and pulse rate)
- Assessment of body temperature (ear)
- Respiratory rate

- ECG (12-lead)
- Assessment of local tolerability
- Laboratory assessments (including biochemistry, hematology, coagulation and urinalysis)
- Blood sampling for Anticalin PK
- Blood sampling for hepcidin assessment
- Blood sampling for PD, both fast components (i.e. iron, transferrin saturation and ferritin) and slow components (i.e. reticulocytes, reticulocyte Hb)
- Laboratory tests as listed in Section 9.4.1.1, with the exception of serology (HIV-AB, HBs-AG, and HCV-AB)

In addition to the 240-hr follow-up examinations, for each of the two study stages a final visit will be conducted 28 days (±2) after the last administration of study drug; this visit will be conducted to collect a final blood sample for ADA, hepcidin and pharmacodynamic assessments.
During this visit, the following assessments will be conducted:

- Blood sampling for ADA, hepcidin, and pharmacodynamic assessments
- Adverse event questioning
- Concomitant medication questioning

No medical treatment is planned after the end of the study.
However, subjects exhibiting either subjective or objective abnormalities when the study has been completed will be followed up. Any AE which remains unresolved after completion of the study requires detailed evaluation, follow-up, and if necessary specific medical treatment until the AE is resolved or a reasonable explanation for its persistence is found.

If the subject refuses to follow the instructions of the investigator, the latter is released from responsibility.

For each of the two study stages, the end of the study is defined as the last visit (defined as last contact) of the last subject undergoing the study.

# 6 DISCUSSION OF STUDY DESIGN, INCLUDING THE CHOICE OF CONTROL GROUPS

The study described in this protocol will be conducted to establish the safety, pharmacokinetics, and pharmacodynamics of the novel Anticalin PRS-080#022-DP in healthy volunteers. Since non-clinical data are not always predictive of clinical behavior of the compound tested, a study in healthy human subjects has to be performed.
The individual stages of the study are designed to determine the safety and maximum tolerated dose of single and repeated intravenous infusion with PRS-080#022-DP (Stage 1 and Stage 2, respectively).
A double-blind design is used to eliminate reporting bias. Placebo is considered an appropriate control in the absence of an active comparator with a mechanism of action comparable to that of PRS-080#022-DP.
The Stage 1 study stage (SAD) has been designed to provide maximum safety to the subjects, limiting exposure of previously untested doses to one subject in the first 24 h period. Using a standard 6+2 design, doses will be explored in cohorts of 8 subjects, with 6 subjects receiving the study drug and 2 subjects receiving placebo. Dose cohorts will be split into 2 sub-groups (2+6, 1 placebo-treated subject in each sub-group; the main group of 6 subjects will be also

divided into two subgroups), treated sequentially at 24 h intervals to allow sufficient time for observation of acute toxicity in the first study-drug treated subject to receive any given dose; additional caution is applied to the first dose level, with this cohort being divided into 4 subgroups (2+2+2+2). With exception of the pilot group in each cohort the remaining subjects will be treated sequentially at 150 minutes intervals between each start of infusion to allow sufficient time for observation of acute toxicity of the IMP. A Dose Escalation Committee will provide recommendations about stopping, modifying or continuing the trial; the decision to escalate the dose will be based on an interim analysis of safety and tolerability results up to 72 hr after dosing.
To maintain safety of subjects treated in the Stage 2 study stage (MAD), the starting dose will be two dose levels below the MTD observed in the SAD study stage or, if no MTD was observed, 1 dose level below the highest dose group tested.
Healthy males (18 to 50 years old) are chosen as study population. Potential gender-dependent PK/PD effects due to significantly different hepcidin levels in males and females that are 18 to 50 years old will be investigated in subsequent clinical trials.

# 7 SELECTION OF STUDY POPULATION

The following inclusion and exclusion criteria are chosen to select the appropriate study population regarding homogeneity in order to meet the requirements for reliable evaluation of the data collected.

The following recruitment measures will be used:
- Pre-selection of subjects from the Nuvisan electronic volunteer's database which fit to key criteria. Subjects will be contacted by mail or/and by phone asking for their interest to participate in this study
- Advertisement in the lobby of Nuvisan's clinical facilities
- Advertisement on Nuvisan's volunteer home page
- Advertisement in the local and regional newspapers, if necessary.

Eligible subjects will be included in the study after having given voluntary written informed consent before the first screening examination procedure.

## 7.1 INCLUSION CRITERIA

Subjects are required to meet all of the following inclusion criteria:
1. Subjects should be healthy Caucasian male subjects: based on a screening examination including medical history, physical examination, 12-lead ECG, vital signs and clinical laboratory profiles, age 18-50 years, inclusive
2. BMI 18-30 kg/$m^2$ and body weight 60-90 kg inclusive
3. Subjects must be using two acceptable methods for contraception (e.g. spermicide and condom) during the study and refrain from fathering a child in the 3 months following the last dosing.
4. Subject is able to read the subject information sheet, to understand information about the study and has signed the informed consent sheet.

## 7.2 EXCLUSION CRITERIA

Subjects meeting any of the following criteria will be excluded from the present study:

1. Known or suspected of not being able to comply with the trial protocol and/or clinical unit restrictions
2. Any uncontrolled or active major systemic disease including, but not limited to: pulmonary, gastrointestinal, metabolic, urogenital, neurological, immunological, psychiatric, or neoplastic disorder
3. Diseases or conditions known to interfere with the absorption, distribution, metabolism or excretion of drugs
4. History or presence of malignancy
5. Definite or suspected history of drug allergy or drug hypersensitivity or intolerance to PEG
6. Active acute or chronic infection, including, but not limited to: upper airway infection, urinary tract infection, and skin infection
7. Clinically significant illness within 30 days prior to the planned first drug administration
8. Use of any investigational drug within 30 days, or 5 half-lives, whichever is longer, prior to the planned first drug administration
9. Subjects who have participated in a previous cohort/study stage
10. Use of prescription medication within 14 days prior to the planned first drug administration and throughout the study (with the exception of medications given to treat an adverse event (e.g., paracetamol 2 g or ibuprofen 800 mg per day for 3 consecutive days are permissible, but not within 48 hours prior to the planned first drug administration)
11. Use of non-prescription or over-the-counter medications is prohibited within 7 days prior to the planned first drug administration and throughout the study. This includes all vitamins, herbal supplements, or remedies
12. Smoking greater than 20 cigarettes per week
13. History of alcohol or substance abuse within the past 6 months prior to the planned first drug administration
14. History of increased bleeding risk
15. Clinically relevant abnormalities found in physical examination, vital signs measurements, laboratory safety tests or ECG, e.g. QTc according to Bazett: QTc > 450ms, PQ > 220ms, QRS > 120ms;
16. Supine pulse rate not within 45 to 90 beats per minute and/or supine blood pressure Systolic < 90 >145 mmHg and diastolic < 40 > 95 mmHg
17. Blood donation within the last 60 days prior to the planned first drug administration
18. Positive results on the following Screening laboratory tests: alcohol breath test, urine test on drugs of abuse, hepatitis B surface antigen, hepatitis C antibody, and human immunodeficiency virus (HIV1/2) antibodies
19. Any condition that would jeopardize the subject’s appropriate participation in this study
20. Iron overload or disturbance in utilization of iron (defined as ferritin > 300.0 ng/mL and < 10.0 ng/mL)
21. i.v. iron treatment or blood transfusion within last 90 days prior to the planned first drug administration or during trial
22. ESA (e.g. Erythropoietin) treatment within the last year

23. Surgery or trauma with significant blood loss within 2 months before the planned first drug administration
24. Not able to abstain from consumption of food or beverages known to influence dietary iron absorption 12 hours prior to any study visit and during the study confinement periods:
    - Caffeine-containing beverages or food (tea, coffee, cola, chocolate, etc.)
    - Quinine-containing beverages or food (bitter lemon, tonic water)
    - Grapefruit juice (sweet or sour)
    - Poppy seeds-containing beverages or food
25. Known intolerance against investigational medicinal product or excipients
26. Subject is an employee of the sponsor, the investigator or the institution of the investigator

## 7.3 REMOVAL OF SUBJECTS FROM THERAPY OR ASSESSMENT

### 7.3.1 PREMATURE DISCONTINUATION OF THE STUDY PER SUBJECT

A subject can decide to withdraw from the study participation at any time, for any reason, specified or unspecified, and without penalty or loss of benefits to which the subject is otherwise entitled. In this case, the subject must immediately contact the investigator and state that he is leaving the study.
The subjects should be informed of the possibility to withdraw consent without giving any reason and to require that all previously retained identifiable samples will be destroyed to prevent future analyses, according to national provisions. The subject information should include a statement that the consequence of the subject's withdrawal of consent will be that no new information will be collected from the subject and added to existing data or a database.

<u>The investigator has to withdraw a subject from the trial in the following cases:</u>
- occurrence of an adverse event which does not justify a continuation of the study
- impossibility to obtain samples
- protocol violation which jeopardizes the performance of the study

Individual subject stopping criteria (for dose escalation or continued dosing within a cohort):
1. Dosing will be stopped immediately if clinically relevant allergic reaction/ hypersensitivity (e.g. fever > 39°C, dyspnea) occurs which needs medical intervention.
2. Dosing will be stopped if body temperature increases by > 1° C during infusion (body temperature will be measured every 15 min during the infusion time).
3. Dosing will be stopped if ALT increases > 3 x ULN.
4. Dosing will be stopped if Conjugated Bilirubin increases ≥ 1.5 x ULN.
5. Dosing will be stopped if serum creatinine increases > 1.5 x ULN or any increase of 27 µmol/L from baseline.
6. Dosing will be stopped if subjects suffer from a drug related (assessed as possibly or probably) and clinically relevant AE of moderate or severe intensity.

Individual subject stopping criteria for the MAD phase only:
1. Increase of hemoglobin >2 g/dL above ULN or above baseline if baseline is above ULN.

2. Hematocrit value ≥ 55.0 %.

In the event of a subject deciding to stop participation in the study, he is requested to take part in the final medical examination including the required blood withdrawal (for the laboratory tests). This final examination is for the subject's safety. It is only by this examination that any impairment to the subject's health which may require treatment and could be related to the subject's participation in the study can be detected.

As the aim of this study is to generate information on the tolerability and pharmacodynamic properties of the substance under investigation as well as pharmacokinetic data, subjects who are withdrawn from the study for reasons other than safety issues may be replaced at the discretion of the Sponsor and the Investigator.

In case of subject withdrawal, the investigators will attempt to perform all protocol-defined end-of-treatment assessments and document the main reason for withdrawal in the CRF. If the subject is withdrawn for safety reasons, the investigator will make thorough efforts to document the final AE/SAE outcome.

No transition to the next dose cohort during the SAD phase can occur before blinded review of Laboratory and Safety data up to 72 hours by the DEC after dosing of the previous cohort.
Dosing to the next higher dose level will be suspended if any of the following occur:

1. 4 or more subjects in the dose cohort meet one of the individual stopping criteria.
2. 3 or more subjects in the dose cohort experience treatment related clinically relevant (i.e. moderate and/or severe) AEs,
3. 1 or more subjects in the dose cohort experience a treatment related SAE.

Dose de-escalation to an intermediate or lower dose level during the SAD will allow enrolment of up to 8 further patients, according to recommendations by the DEC.
If the SAD study was stopped at one dose level due to clinically relevant toxicity, the maximum dose appropriate for the MAD stage is defined in that case as the dose level below the dose inducing the relevant toxicity.

No transition to the next dose cohort during the MAD phase can occur before blinded review of Laboratory and Safety data up to 72 hours after dosing of the previous cohort.
Dosing to the next higher dose level will be suspended if any of the following occur:

1. 6 or more subjects in the dose cohort meet one of the individual stopping criteria.
2. 4 or more subjects in the dose cohort experience treatment related clinically relevant (i.e. moderate and/or severe) AEs,
3. 1 or more subjects in the dose cohort experience a treatment related SAE.

### 7.3.2 PREMATURE DISCONTINUATION OF THE STUDY AT THE TRIAL SITE

The sponsor may discontinue the conduct of the study at the selected study site under the following conditions:
- Failure of the investigators to comply with relevant regulations.
- Insufficient adherence to protocol requirements.
- Insufficient enrolment of subjects.

### 7.3.3 PREMATURE DISCONTINUATION OF THE COMPLETE STUDY

The sponsor may discontinue the complete study at any time, for ethical or scientific reasons, in agreement with Nuvisan GmbH. This includes the discovery of any unexpected, serious, or unacceptable risk to the subjects enrolled in the study. The principal investigator is entitled at any time to stop the study due to medical reasons. In such a case, he should consult the sponsor at the earliest opportunity.
If a trial is prematurely terminated or suspended, the sponsor will inform Nuvisan GmbH promptly.

# 8 TREATMENTS

## 8.1 TREATMENTS TO BE ADMINISTERED

Subjects in each stage and cohort will be randomized to receive either PRS-080#022-DP or placebo in the appropriate ratio. Randomization will be done by the Clinical Trial Supplies Department of Nuvisan. Subjects will receive 1 treatment (Stage 1) or 7 treatments (Stage 2) during the study. Drug administrations will take place in the morning with appropriate intervals between subjects to allow for sample collection and any necessary additional clinical observations. The respective treatments will consist of the following:

| Study | Treatment | Regimen | Dose | Number of subjects |
|---|---|---|---|---|
| Stage 1 | Intravenous dose of PRS-080#022-DP or placebo (Single dose) | Administration on Day 1 | 6 dose levels, from 0.08 mg/kg BW to 16 mg/kg BW | 6 verum + 2 placebo |
| Stage 2 | Intravenous dose of PRS-080#022-DP or placebo (Multiple dose) | Administration on Days 1, 3, 5, 7, 9, 11, 13 | 2 dose levels, to be determined after Stage 1 | 9 verum + 3 placebo |

PRS-080#022-DP (IMP) will be provided to Nuvisan GmbH by Pieris AG in sufficient quantity. IMP is supplied as frozen vials containing 8.1 mg/mL protein (+/- 15%), as solution for injection. IMPs will be stored at Nuvisan in a freezer at -20°C; after removal from freezer the IMP can be kept in a refrigerator/refrigeration unit at 2-8°C for 24 hours. **The IMP must not be allowed to freeze again.** The solution should be visually inspected prior to use. Only clear solutions without particles should be used.

Preparation of one single dose per subject per treatment day will be calculated as follows:

| mL [volume study drug] = XX x kg [BW] x Dose Level [mg/kg BW] x 1 / 8.1 [concentration] |
|---|

Calculations are based upon body weight measurements at Day -1; body weight (BW) is to be documented in the CRF.

Detailed information on IMP handling will be provided in an IMP-handling manual that is based on information provided in the Sponsor's pharmaceutical instruction.

Treatment with study drug or placebo will be administered intravenously into the arm contralateral to the arm used for blood collection at a constant rate of 125 mL per hour over 120 minutes. The volume of the infusion system will be filled with a sufficient quantity of diluted solution in order to guarantee that 250 mL of the diluted solution can be administered. The dispensing of medication for administration will follow the randomization list.

Intravenous administration:
Immediately prior to administration, the assigned unblinded personnel dispense the study drug according to the treatment arm and calculated dose of the subject. The 250 mL infusion bag, pre-filled with diluted IMP must be visually inspected for particulate matter and discoloration prior to administration. Infusion bags exhibiting particulate matter or discoloration must not be used. The calculated amount of PRS-080#022-DP will be transferred into an infusion bag and diluted in 0.9% NaCL solution to a final volume of 250 mL. The infusion bag should be labelled with the following minimum information: the subject identification, and prescribed PRS-080#022-DP dose. The label must not identify the treatment arm the subject is randomised to. The assigned unblinded personnel are responsible for ensuring that the treatment arm the subject has been randomised to at the baseline visit is adhered to.
The study drug should be used within 24 hours of filling the infusion bag, if stored at 2-8°C.
Intravenous administration will be performed into the arm contralateral to the arm used for blood collection by a 120-min infusion (rate of flow 125 mL/h). Infusions of the sterile solutions will be given through a infusion set and an i.v. catheter with the rate controlled by the Infusomat.
After the administration of 250 mL IMP solution, 5 mL 0.9% NaCl solution will be administered as bolus through the system.

## 8.2 IDENTITY OF INVESTIGATIONAL PRODUCTS

Test product

**Investigational Product 1**

| | | |
|---|---|---|
| Name | : | PRS-080#022-DP |
| Substance | : | PRS-080#022 |
| Formulation | : | PBS pH 6.5 (20 mM $NaH_2PO_4$; 115 mM NaCl; pH6.5) |
| Strength | : | 8.1 mg/mL |
| Batch/Lot no (Number). | : | to be noted in the trial master file |
| Expiry date | : | to be noted in the trial master file |
| Origin | : | Human NGAL protein |

Reference product

**Investigational Product 2**

| | | |
|---|---|---|
| Name | : | Placebo |
| Substance | : | PRS-080-Placebo#001 |
| Formulation | : | PBS pH 6.5 (20 mM $NaH_2PO_4$; 115 mM NaCl; pH6.5) |
| Strength | : | N/A |
| Batch/Lot no. | : | to be noted in the trial master file |
| Expiry date | : | to be noted in the trial master file |
| Holder of the Marketing authorization | : | N/A |

The sponsor will provide Nuvisan GmbH with investigational products manufactured and tested according to applicable GMP requirements for clinical trial supplies together with a certificate of analysis and a confirmation that the investigational products are released for human use in clinical trials.

The sponsor will provide Nuvisan GmbH concerning a positive BSE / TSE risk evaluation (BSE certificate), e.g. according to the standard provided by Nuvisan ("BSE TSE Risk Evaluation") or a BSE/TSE certificate provided by the manufacturer of the investigational products. He will confirm that the required safety of the drug intended for the clinical trial is warranted according to the "Note for Guidance on Minimizing the Risk of Transmitting Animal Spongiform Encephalopathy Agents via Human and Veterinary Medicinal Products" (EMEA/410/01, Rev. 2, 2. Oct 2003). In addition, the sponsor will assure that for the manufacturing of active and inactive ingredients no substance of bovine source was used originating either from Great Britain or Switzerland.

The sponsor will assure that the drugs, PRS-080#022-DP and Placebo, are identical in their appearance, shape. Thus, neither the subject nor the investigators will be aware of whether the drug administered is the test or the reference drug.

Handling Requirements:
The designated unblinded person at the study site will be responsible for ensuring that the study drugs are stored in compliance with GMP in a freezer at -20°C, and in a locked refrigerator (+2ºC to +8ºC) after reconstitution prior to administration with limited access and in accordance with the instructions on the study medication labels.

## 8.3 METHOD OF ASSIGNING SUBJECTS TO TREATMENT GROUPS

A computer-generated randomization scheme will be provided by the designated unblinded personnel at the CRO.
Only subjects who comply with all selection criteria will be enrolled into the trial. Prior to the first administration the subjects will be given the lowest available number on the randomization list (number 001 …). This number assigns them to one of the treatment arms. The randomization number will be entered on the CRF. The subject's randomization number will be cross-referenced by the subject's unique Nuvisan subject number.

The lowest randomization number will be assigned to the first subject in the study; the second lowest number will be used for the second subject and so on. However, precedence will be given to subjects who participate in a trial at Nuvisan GmbH for the first time and for subjects who served as "stand-by" during a preceding trial
Each subject enrolled in the study will be uniquely identified by this random number. The subject's random number will appear on all study documents relating to that subject and will be cross-referenced by the subject's unique Nuvisan subject number.
Assignment of subjects to treatment will be dictated by the stage and dose cohort open at the time of the subjects' participation.
In case of replacement of subjects, the randomization of the replaced subjects will be the same as the randomization of the subjects they substitute for, starting with the No.100 (e.g. subject 005 is replaced with 105).

## 8.4 SELECTION OF DOSES IN THE STUDY

Based on the toxicology results of the 4-week study in cynomolgus monkeys the starting dose for the escalation study has been calculated using the NOAEL approach. The NOAEL approach was justified instead of the Minimum anticipated biological effective level (MABEL) approach since the target and mode of action of PRS-080#022-DP are known, and since toxicology studies were performed in a relevant animal species, cynomolgus monkeys, where the mode of action could be confirmed. Furthermore, PRS-080#022 has an antagonistic mode of action and does not possess an Fc domain of antibodies. Immunotoxicological evaluation during the 4-week safety study did not reveal any indication for the triggering of cytokine release in monkeys.
In the 4-week toxicology study in cynomolgus monkeys, a NOAEL of 48 mg/kg protein (120 mg/kg for the PEGylated product) when given every 48h was determined. This was the highest dose examined in the study. Based on this good tolerability of PRS-080#022 in the non-human primate, a safety factor of 10 appears justified to determine the starting dose. Thus, the HED of 15.5 mg/kg protein (39 mg/kg PEGylated product) would be reduced to 1.55 mg/kg protein (3.9 mg/kg PEGylated product) as a safe starting dose using the NOAEL approach. However, as a pharmacological effect, namely PRS-080#022-mediated iron mobilization in cynomolgus, is already observed at 0.4 mg/kg protein (1 mg/kg PEGylated product), but not at 0.2 mg/kg protein (0.5 mg/kg PEGylated product), 0.08 mg/kg protein is the recommended safe starting dose for the SAD phase of the FIH trial. This dose is not expected to elicit a pharmacodynamic response.
The benign toxicity profile and the linear dose-response relationship observed in the non-human primate also justifies an escalation profile of 0.08 – 0.4 – 1.2 – 4 – 8 – 12 – 16 mg/kg protein content in this prospective healthy volunteer study. Mean hepcidin levels in patients with ACD can be 10 to 20-fold higher than in healthy subjects. Therefore, in this Phase I study PCS_01_12 a dose range up to 16 mg/kg is targeted. The highest dose group corresponds to the NOAEL dose level.

## 8.5 SELECTION AND TIMING OF DOSE FOR EACH SUBJECT

The Stage 1 and Stage 2 studies described in this protocol are both ascending dose studies, designed to ascertain the maximum tolerated dose of intravenously administered PRS-080#022-DP in single dose and multiple dose settings, respectively.

The decision to escalate dose will be made by a specially appointed Dose Escalation Committee (DEC). The DEC will provide recommendations about stopping, modifying or continuing the trials. The DEC will meet for cohort safety review to provide dose escalation recommendations (see Section 1.2 for details of the DEC, and see Section 7.3.1 for details of stopping rules).
The decision to dose subsequent sub-groups at the same dose level will be based on an interim analysis of clinical safety and safety laboratory data 24 hours after start of infusion.
The decision to escalate dose will be made according to pre-defined stopping rules, and will be based on an interim analysis of clinical safety and safety laboratory data 24 hours and 72 hours after the start of infusion.
The maximum tolerated dose (MTD) in the Stage 1 and Stage 2 studies will be the dose level below that at which treatment is stopped due to one (or more) of the stopping rules described in Section 7.3.1.

### 8.5.1 DOSE ESCALATION IN STAGE 1 AND STAGE 2

Stage 1: SAD

*Cohort 1*
The first dose cohort consists of 4 sub-groups (A: 2 subjects; B: 2 subjects, C: 2 + 2 subjects). In this dose cohort, 2 subjects will be treated upfront (sub-group A); one of these subjects will receive placebo and the other subject will receive verum.
Only if this dose was well tolerated (based on 24 hours safety assessment), the next two subjects (sub-group B, 1 or 2 = verum, 1 or 0 = placebo) will be dosed to maintain blinding.
Only if this dose was well tolerated (based on 24 hours safety assessment) the remaining 4 subjects (sub-group C: 2+2, 3 or 4 = verum, 1 or 0 = placebo) will be dosed.
The time difference between sub-groups A, B and C must be at least 24 hours after dosing of the two preceding subjects.
Dosing in sub-groups B and C will be started sequentially with an interval of at least 1 hour150 minutes between each individual.

*Cohorts 2-6*
Dosing to the next higher dose level will be permitted in the absence of any of the stopping rule scenarios described in Section 7.3.1.
Subsequent dose cohorts will each consist of 2 sub-groups (A: 2 subjects; B: 6 subjects). In each subsequent dose cohort, 2 subjects will be treated upfront (sub-group A); one of these subjects will receive placebo and the other subject will receive verum.
Only if this dose was well tolerated (based on 24 hours safety assessment) the remaining 6 subjects (sub-group B, 5 = verum, 1 = placebo) will be dosed.
The time difference between sub-groups A and B must be at least 24 hours after dosing of the two leading subjects.
Due to possible circadian effects and the size of subgroup B, subjects in subgroup B will be dosed during the first half of two consecutive days (dosing started before 12 noon)[13].

[3]: In any case, the maximum storage conditions for the prepared infusion bags at different temperatures (-20°C, 2-8°C and RT) always must be considered and be in accordance with the pharmacy manual of Pieris AG.

With exception of the pilot group A, in each cohort the remaining subjects in each sub-group will be treated sequentially at 150 minutes intervals between each start of infusion to allow sufficient time for observation of acute toxicity of the IMP.

Stage 2: MAD
Dosing in each dose cohort will be started sequentially with an interval of at least 30 minutes between each individual.
Dosing to the next higher dose level will be suspended if any of the following occur:
1. 6 or more subjects in the dose cohort meet the stopping criteria (see Section 7.3.1).
2. 4 or more subjects in the dose cohort experience treatment related clinically relevant (i.e. moderate and/or severe) AEs,
3. 1 or more subjects in the dose cohort experience a treatment related SAE.

### 8.5.2 STAGE 1: SINGLE ASCENDING DOSE STUDY STAGE (SAD)

Each subject will be assigned to receive a single dose of either the study drug or placebo in a randomized fashion, at a dose of 0.08 - 16 mg/kg BW. Drug administrations will be performed in the morning on the day after admission to the study unit. The day of drug administration will be defined as Day 1.
This study will explore 6 dose levels from 0.08 mg/kg BW to 16 mg/kg BW (see Table 3). Subjects will be assigned to the dose cohort open at the time of their involvement in the study. The rules governing dose escalation are described in Section 7.3.1.

**Table 3: Stage 1 dose cohorts**

| Cohort | Dose Level | Number of subjects |
|---|---|---|
| 1 | 0.08 mg/kg BW | 6 verum + 2 placebo |
| 2 | 0.4 mg/kg BW | 6 verum + 2 placebo |
| 3 | 1.2 mg/kg BW | 6 verum + 2 placebo |
| 4 | 4 mg/kg BW | 6 verum + 2 placebo |
| 5 | 8 mg/kg BW | 6 verum + 2 placebo |
| 6 | 16 mg/kg BW | 6 verum + 2 placebo |

### 8.5.3 STAGE 2: MULTIPLE ASCENDING DOSE STUDY STAGE (MAD)

Each subject will be assigned in a randomized fashion to receive 7 doses of either the study drug or placebo. Drug administrations will start in the morning on the day after admission to the study unit. The day of first drug administration will be defined as Day 1. The study drug/placebo will be administered once every other day (48-h intervals) or less frequently depending on the PK/PD results obtained in the SAD stage (Stage 1), from Days 1 to 13.
This study will explore two dose levels (Table 4), with the doses to be determined based on findings of the Stage 1 study; dose levels will be limited to 1 to 2 dose levels below the MTD dose of the SAD study or, if no MTD was observed, 1 dose level below the highest dose group tested.

**Table 4: Stage 2 dose cohorts**

| Cohort | Dose Level | Number of subjects |
|---|---|---|
| 1 | TBD | 9 verum + 3 placebo |

| | | |
|---|---|---|
| 2 | TBD | 9 verum + 3 placebo |

Subjects enrolled in the Stage 2 study will be assigned to the dose cohort open at the time of their involvement in the study. The rules governing dose escalation are the same as those used in the Stage 1 study, and are described in Section 7.3.1.

## 8.6 LABELING AND PACKAGING OF THE INVESTIGATIONAL PRODUCT(S)

The drugs will be provided by the sponsor with appropriate labeling.
The sponsor will supply sufficient trial medication. The medication will be identified by project and protocol number, packing number, expiry date, storage requirements and contents.
The sponsor will provide a Certificate of Analysis.
The study products will be labeled in accordance with the GCP ordinance ("GCP-Verordnung") § 5.
The labels on the vial and secondary packaging (box) of the medicinal product include in German language:

- Sponsor's study code
- Sponsor's name, address and phone number
- CRO: Name, address and phone number
- EudraCT number
- Product name, strength and dosage form
- Application form
- Content by weight, volume, number of units
- Route of administration
- Directions for use
- Batch number
- Use-by date
- Storage instructions
- The term "For clinical trial use only"
- Instruction for return

Vial and Box label are printed as follows:

**Vial (10 ml) – IMP - Infusion**

CRO: Nuvisan, Wegenerstr. 13, D-89231 Neu-Ulm
Sponsor: Pieris AG, D-85354 Freising, Lise-Meitner-Str. 30
5 ml PRS-080#022-DP
Proteingehalt 8,1 mg pro ml
Konzentrat zur Herstellung einer Infusionslösung
Zur langsamen intravenösen Infusion
Anwendung und Dosierung gemäß Prüfplan und pharmazeutischer Anleitung
Prüfplancode: PCS_01_12
Probanden ID:
Ch.-B.:

**Vial (10 ml) – Placebo - Infusion**

CRO: Nuvisan, Wegenerstr. 13, D-89231 Neu-Ulm
Sponsor: Pieris AG, D-85354 Freising, Lise-Meitner-Str. 30
5 ml Placebo
Konzentrat zur Herstellung einer Infusionslösung
Zur langsamen intravenösen Infusion
Anwendung und Dosierung gemäß Prüfplan und pharmazeutischer Anleitung
Prüfplancode: PCS_01_12
Probanden ID:
Ch.-B.:

## Box - IMP - Infusion

CRO: Nuvisan, Wegenerstr. 13, D-89231 Neu-Ulm, Telefon Nr.: +49 (0)731 98 40 0
Sponsor: Pieris AG, D-85354 Freising, Lise-Meitner-Str. 30, Telefon Nr.: +49 (0) 8161 14 11 400
Nur zur klinischen Prüfung bestimmt
…….. Injektionsflaschen mit je 5 ml PRS-080#022-DP
Proteingehalt 8,1 mg pro ml
Konzentrat zur Herstellung einer Infusionslösung
Zur langsamen intravenösen Infusion
Anwendung und Dosierung gemäß Prüfplan und pharmazeutischer Anleitung
EudraCT Nr.: 2012-001450-26
Prüfplancode: PCS_01_12
Lagerung bei -20 +/- 5°C
Unverbrauchte Fläschchen samt Sekundärverpackung am Ende der Studie an den Sponsor zurückgeben.
Ch.-B.:
Verwendbar bis: mm/ yyyy

## Box - Placebo - Infusion

CRO: Nuvisan, Wegenerstr. 13, D-89231 Neu-Ulm, Telefon Nr.: +49 (0)731 98 40 0
Sponsor: Pieris AG, D-85354 Freising, Lise-Meitner-Str. 30, Telefon Nr.: +49 (0) 8161 14 11 400
Nur zur klinischen Prüfung bestimmt
........ Injektionsflaschen mit je 5 ml Placebo
Konzentrat zur Herstellung einer Infusionslösung
Zur langsamen intravenösen Infusion
Anwendung und Dosierung gemäß Prüfplan und pharmazeutischer Anleitung
EudraCT Nr.: 2012-001450-26
Prüfplancode: PCS_01_12
Lagerung bei -20 +/- 5°C
Unverbrauchte Fläschchen samt Sekundärverpackung am Ende der Studie an den Sponsor zurückgeben.
Ch.-B.:
Verwendbar bis: mm/ yyyy

Each manufacturing/packaging process will be performed and documented in conformity with Good Manufacturing Practice (GMP). If necessary, internal drug names and batch numbers will be assigned.
Nuvisan will be responsible for the relabeling of the IMP to extend the use-by date in compliance with regulatory requirements including its proper documentation in case this will be necessary and requested by Sponsor.

## 8.7 BLINDING AND BREAKING THE BLIND

Nuvisan will generate the randomization schedule for the study, which will be held under secure conditions of restricted access.
To keep the double-blind design of the study it is necessary to involve unblinded person(s) at the study site for handling, and dispensing of the study drug. Unblinded person(s) must not perform any assessments.

Subjects, Investigators and investigators staff, persons performing the assessments or being responsible for determining dosing regimen/adjustments, and staff of the sponsor or data analysts, will remain blinded from the time of randomization until database lock, using the following methods: randomization data, including any documentation identifying the treatment allocation, are kept strictly confidential until the time of unblinding with the following exceptions; staff responsible for study drug management (i.e. the staff in Nuvisan's CTS unit preparing the IMP).

Breaking of the blind (PRS-080#022-DP and Placebo), by opening an emergency envelope, will be expressly forbidden except in the event of a medical emergency where the identity of the drug has to be known in order to properly treat the subject. The Sponsor, the clinical study monitor and the QPPV has to be informed immediately of occasions where a code has been broken.

The Sponsor and Nuvisan acknowledged that FGK is entitled for unblinding of the study medication for a specific subject for safety reasons according to the Decoding Procedure for blinded trials described in FGK's SOP for Safety Reporting-Drug that will be provided to Nuvisan by FGK.

NUVISAN will ensure that unblinding of the randomization code for the double-blinded studies of the Clinical Trial only occurs in accordance with this Protocol and via the Sponsor´s QPPV, located at FGK, to ensure compliance with the Decoding Procedure for blinded trials provided by FGK. Therefore, every effort must be made to contact the sponsor's QPPV prior to breaking the code. If this is not possible and the situation is an emergency the investigator may break the code and contact the sponsor's QPPV as soon as possible thereafter.

Nuvisan shall promptly report and justify to Sponsor and FGK any unblinding of the IMP by Investigator(s), no matter if premature (e.g., accidental or due to a SAE) or for safety reason (e.g., in a for the subjects life-threatening situation). NUVISAN shall maintain documentation of such unblinding.

The date and time of unblinding, the name of the study personnel responsible, and the reason for the unblinding must be documented in the CRF and on the individual code-break envelope. In each case where the code is broken, that subject has to be excluded from the study.

After completion of the study and after the clinical study report has been signed, all the code-break envelopes, whether opened or not, will be returned, by the clinic pharmacy staff, to the sponsor and filed in the trial master file.

## 8.8 PRIOR AND CONCOMITANT THERAPY

Use of prescription medication within 14 days prior to the planned first drug administration and throughout the study is prohibited, with the exception of medications given to treat an adverse event (e.g., paracetamol 2 g or ibuprofen 800 mg per day for 3 consecutive days are permissible, but not within 48 hours days prior to the planned first drug administration.

Use of non-prescription or over-the-counter medications is prohibited within 7 days prior to the planned first drug administration and throughout the study. This includes all vitamins, herbal supplements, or remedies.

Any concomitant treatment will be documented on the case report forms (CRFs) and listed in the final report.

### 8.9 TREATMENT COMPLIANCE

During the treatment periods, drug administrations will be performed, in accordance with the specifications of the Principal Investigator, by a Nuvisan GmbH staff member. The proper administration of the study medication will be documented on the individual Case Report Form.

Drug Accountability:
The "Clinical Trial Supplies" Department of Nuvisan GmbH will be responsible for the proper storage of all IMPs at site at any time, i.e. until final retrieval / destruction. The study drug must be received by a designated unblinded person at the study site. Upon receipt, the study drug should be stored according to the instructions specified on the Box label of the drug in an appropriate secured, GMP conform location. Each movement will be documented in an inventory list for drug accountability.
Sponsor will ensure timely delivery of IMP to the clinical site and Nuvisan is responsible for drug accountability. Therefore, Nuvisan shall maintain records that document shipment according to the specifications, receipt, dispensing, handling, return and destruction of the IMP in accordance with all applicable laws and regulations. The reconciliation of IMP will be monitored by FGK on a regular basis, which shall be allowed and supported by Nuvisan, and Nuvisan will provide Sponsor with a final reconciliation of IMP after termination of the Study. If FGK considers this to be necessary, Nuvisan will accept and use form sheets provided by FGK for drug accountability, and will distribute such forms to the pharmacy of the clinical site and ensure consistent usage of such forms during the Study.
Nuvisan will maintain (i) a system for retrieving IMP (e.g., as needed for a Recall of IMP) and documentation of such system as well as (ii) a system for disposition/destruction of unused IMP and documentation of such system.
Unused IMP will be returned to Sponsor or will be destroyed at the clinical site by Nuvisan according to Sponsor’s written instructions. Both of above, referred return and destruction, need Sponsor’s approval.

## 9 VARIABLES TO BE ASSESSED

### 9.1 EFFICACY VARIABLES

Not applicable to the present study.

### 9.2 PHARMACODYNAMIC VARIABLES

#### 9.2.1 SUMMARY OF SUBSTANCES TO BE ANALYZED

In each of the two study stages described in this protocol, pharmacodynamic assessment will include assessment of the extent of target (hepcidin) engagement by PRS-080#022-DP, measurement of biomarkers with a fast response kinetic i.e. iron and transferrin saturation, and biomarkers with a slow response kinetic, i.e. Hb, reticulocytes, reticulocyte Hb, and ferritin. In addition, blood samples will be collected for exploratory biomarker assessment.
Further details of these substances and their determination are given in Section 9.2.4.

### 9.2.2 TIME POINTS/INTERVALS FOR SAMPLE COLLECTION

Blood Samples:
For the evaluation of the circulating concentrations of the hepcidin and the pharmacodynamic biomarkers, blood samples will be drawn by venous puncture or indwelling venous catheter into the appropriate tubes as shown in Table 5. Table 5 also summarizes the amount of blood that will be withdrawn in order to obtain a minimum required amount of the appropriate matrix per time point.

**Table 5: Pharmacodynamic sample collection**

| Analyte | Matrix | Container | Blood vol. collected per sample (mL) |
|---|---|---|---|
| **Hepcidin** | Plasma | Lithium-Heparin S-Monovette® 2.7 mL, Sarstedt order no.: 04.1929 | 2.7 |
| **Fast response kinetic PD[a]** | Serum | Serum-Gel S-Monovette® 4.7 mL, Sarstedt order no.: 0.3.1524 | 4.7 |
| **Slow response kinetic PD[b]** | Whole blood | $K^3$-EDTA Monovette®, 2.7 mL, Sarstedt order no.: 04.1917 | 2.7 |
| **Exploratory biomarkers[c]** | Serum | Serum Gel Monovette® for Cytokines, Sarstedt order no.: 04.1905 | 2.6 |

a: Iron, ferritin and transferrin saturation
b: Hb, reticulocytes, reticulocyte Hb
c: e.g. soluble transferrin receptor, cytokines

*Hepcidin assessment*
For the evaluation of the plasma concentrations of hepcidin, blood samples will be drawn by venous puncture or indwelling venous catheter into Lithium-Heparin S-Monovettes®. An amount of 2.7 mL blood will be withdrawn (Table 5; please see Laboratory Manual for subsequent sample handling).
Blood samples for the determination of plasma concentrations of hepcidin will be drawn at screening, at regular intervals throughout the subjects' hospitalization period, and during the ambulatory visits conducted at 120 h, 240 h and 28 days after the last study drug administration. Sampling time points for both stages described in this protocol are summarized in Table 6.

**Table 6: Timing of blood sampling for Hepcidin assessment**

| Study | Time points for Blood sampling (relative to start of infusion) | |
|---|---|---|
| **Stage 1 (SAD)** | Screening, -24 h (*admission*), 0 h (*pre-dose*) and 1 h (*during infusion*) and 2 h, 3 h, 4 h, 6 h, 10 h, 18 h, 24 h, 36 h, 48 h, 72 h, 120 h, 240 h and 28±2 days (*after start of infusion*) | |
| **Stage 2 (MAD)** | Screening, -24 h (*admission*) | |
| | Day 1: | 0 h (*pre-dose*), 1 h (*during infusion*) and 2 h, 3 h, 4 h, 6 h, 10 h, 18 h, 24 h, and 36 h (*after start of infusion*) |
| | Day 3: | 0 h (pre-dose) |
| | Days 5, 7, 9, 11 | 0 h (*pre-dose*) and 2 h (after start of infusion) |
| | Day 13 (last infusion) | 0 h (*pre-dose*), 1 h (*during infusion*) and 2 h, 4 h, 6 h, 10 h, 12 h, 18 h, 24 h, 36 h, 48 h, 72 h, 120 h, 240 h and 28±2 days (*after start of infusion*) |

In each of the two studies described in this protocol, pharmacodynamic assessment will include measurement of pharmacodynamic markers with a fast response kinetic, and pharmacodynamic markers with a slower response kinetic.

*Pharmacodynamic assessment – fast components*

Markers with a fast response kinetic include iron, transferrin saturation and ferritin.

For the evaluation of the serum concentrations of the fast response pharmacodynamic markers, blood samples will be drawn by venous puncture or indwelling venous catheter into Serum Gel S-Monovettes®. An amount of 4.7 mL blood will be withdrawn (Table 5; please see Laboratory Manual for subsequent sample handling).

Blood samples for the determination of serum concentrations of these markers will be drawn at screening and at regular intervals throughout the subjects' hospitalization period, summarized for each study in Table 7; additional samples will be collected at the ambulatory visit conducted 120 h after initiation of study drug administration, during the follow-up examination conducted 240 hours after initiation of study drug administration, and the final visit at 28 days.

**Table 7: Timing of blood sampling for pharmacodynamic assessment – fast response kinetic**

| Study | Time points for pharmacodynamic assessments (fast response kinetic) (relative to start of infusion) | |
|---|---|---|
| **Stage 1 (SAD)** | Screening, -24 h (*admission*), 0 h (*pre-dose*), and 2 h, 4 h, 10 h, 18 h, 24 h, 36 h, 48 h, 72 h, 120 h, 240 h and 28±2 days (*after start of infusion*) | |
| **Stage 2 (MAD)** | Screening, -24 h (*admission*) | |
| | Day 1: | 0 h (*pre-dose*), 2 h, 4 h, 10 h, 18 h, 24 h, and 36 h (*after start of infusion*) |
| | Days 3, 5, 7, 9, 11*: | 0 h (*pre-dose*) |
| | Day 13 | 0 h (*pre-dose*) and 2 h, 4 h, 10 h, 18 h, 24 h, 36 h, 48 h, 72 h, 120 h and 240 h and 28±2 days (*after start of infusion*) |

*Study drug will be administered on Days 3, 5, 7, 9 and 11

*Pharmacodynamic assessment – slow components*

Markers with a slow response kinetic include Hb, reticulocytes, and reticulocyte Hb.

For the evaluation of the whole blood concentrations of the slow response pharmacodynamics markers (Hb, reticulocytes, reticulocyte Hb), blood samples will be drawn by venous puncture or indwelling venous catheter into EDTA $K_3$-EDTA Monovettes®. An amount of 2.7 mL blood will be withdrawn (Table 5; please see Laboratory Manual for subsequent sample handling). Blood samples for the determination of whole blood concentrations of these markers will be drawn at screening, and at regular intervals throughout the subjects' hospitalization period (see Table 8); additional samples will be collected at the ambulatory visit conducted 120 h after initiation of study drug administration, during the follow-up examination conducted 240 hours after initiation of study drug administration and at the final 28-day visit.

**Table 8: Timing of blood sampling for pharmacodynamics assessment during hospitalization – slow response kinetic**

| Study | Time points for pharmacodynamics assessments (slow response kinetic) (relative to start of infusion) | |
|---|---|---|
| **Stage 1 (SAD)** | Screening, 0 h (*pre-dose*), and 10 h, 24 h, 48 h, 72 h, 120 h, 240 h and 28±2 days (*after start of infusion*) | |
| **Stage 2 (MAD)** | Screening | |
| | Day 1: | 0 h (*pre-dose*), 10 h, and 24 h (*post-infusion*) |
| | Days 3, 5, 7, 9, 11:* | 0 h (*pre-dose*) |
| | Day 13 | 0 h (*pre-dose*), 10 h, 24 h, 48 h, 120 h, 240 h and 28±2 days (*after start of infusion*) |

*Study drug will be administered on Days 3, 5, 7, 9 and 11

*Exploratory biomarker analysis*

In each of the two study stages described in this protocol, blood samples will be collected for exploratory assessment of various protein based biomarkers, e.g. soluble transferrin, or cytokines in serum.

For the evaluation of the serum concentrations of cytokines and the biomarker soluble transferrin receptor, blood samples will be drawn by venous puncture or indwelling venous catheter into Serum Gel Monovettes®. An amount of 2.6 mL blood will be withdrawn (Table 5; please see Laboratory Manual for subsequent sample handling).

Blood samples for the determination of serum concentrations of exploratory biomarkers will be drawn at regular intervals throughout the subjects' hospitalization period (see Table 9).

**Table 9: Timing of blood sampling for exploratory biomarker assessment**

| **Study** | **Time points for exploratory biomarker assessments (relative to start of infusion)** | |
|---|---|---|
| **Stage 1 (SAD)** | 0 h (*pre-dose*), and 2 h, 6 h, 24 h, and 72 h (*after start of infusion*) | |
| **Stage 2 (MAD)** | Day 1: | 0 h (*pre-dose*), and 2 h, 6 h, and 24 h (*after start of infusion*) |
| | Days 3 – 11:* | 0 h (*pre-dose*) |
| | Day 13 | 0 h (*pre-dose*), and 2 h, 6 h, 24 h, and 72 h (*after start of infusion*) |

*Study drug will be administered on Days 3, 5, 7, 9 and 11

### 9.2.3 SAMPLE HANDLING PROCEDURES

Sample handling is described separately in a Laboratory Manual "**Laboratory Manual for Sample Collection, Processing and Shipment**"

Each sample will be labeled to indicate not less than: Sponsor name, study number, subject number, period number, and sampling time.

All sample handling procedures, including the time of each sample collection, the time of placement into frozen storage (at the end of the sample workup), and the date of transfer or shipment of the samples to the responsible analyst will be documented in detail.

All samples will be stored for a period of six months after submission of the final report to the sponsor. If no separate contract for further storage has been agreed upon by the sponsor and Nuvisan, the samples will then be destroyed or shipped to Sponsor. Both, return and destruction of samples requires Pieris's approval.

### 9.2.4 APPROPRIATENESS AND DESCRIPTION OF ANALYTICAL METHODS

The concentrations of hepcidin and pharmacodynamic biomarkers in plasma, serum and whole blood (as appropriate) will be determined by different methods. Separate analytical study plans and clinical laboratory protocols will be provided before the start of the analytical parts of the study, including details on method performance for the parameters assessed. Characteristics of the analytical methods will be described in the final report.

### 9.2.5 BLOOD COLLECTION FOR PHARMACODYNAMIC ASSESSMENT

Methods for blood collection for pharmacodynamics assessment will be described in a separate laboratory manual.
The total blood draw per subject for pharmacodynamic assessment, including hepcidin, fast and slow kinetic pharmacodynamics markers, and exploratory biomarkers, will vary for subjects according to the stage of the study they are enrolled in, and will be as follows:

| | Number of samples | Volume of sample (mL) | Total volume (mL) |
|---|---|---|---|
| **Stage 1** | | | |
| Hepcidin | 17 | 2.7 | 45.9 |
| Fast PD | 14 | 4.7 | 65.8 |
| Slow PD | 9 | 2.7 | 24.3 |
| Exploratory PD | 5 | 2.6 | 13 |
| **Total** | | | **149.0** |
| **Stage 2** | | | |
| Hepcidin | 36 | 2.7 | 97.2 |
| Fast PD | 26 | 4.7 | 122.2 |
| Slow PD | 16 | 2.7 | 43.2 |
| Exploratory PD | 14 | 2.6 | 36.4 |
| **Total** | | | **299.0** |

## 9.3 PHARMACOKINETICS

### 9.3.1 ANALYTICAL MEASUREMENTS

#### 9.3.1.1 Summary of Substances to be Analyzed

Plasma concentrations of PRS-080#022-DP will be analyzed by means of a validated method. Further details are given in Section 9.3.1.4.

#### 9.3.1.2 Time Points/Intervals for Sample Collection

Blood Samples:
For the evaluation of the plasma concentrations of the drug substance, blood samples will be drawn by venous puncture or indwelling venous catheter into Lithium-Heparin S-Monovettes®. An amount of 4.9 mL blood will be withdrawn (please see Laboratory Manual for subsequent sample handling).
Time points for sampling during hospitalization for the two stages described in this protocol are summarized in Table 10. Additional samples will be collected during the ambulatory visit conducted 120 hours after the initiation of study drug administration and during the follow-up examination conducted 240 hours after study drug administration.

**Table 10: Timing of blood sampling for Anticalin PRS-080#022-DP pharmacokinetics**

| **Study** | **Time points for Anticalin PRS-080#022-DP PK sampling (relative to start of infusion)** | |
|---|---|---|
| **Stage 1 (SAD)** | 0 h (*pre-dose*) and 1 h (*during infusion*) and 2 h, 3 h, 4 h, 6 h, 10 h, 18 h, 24 h, 36 h, 48 h, 72 h, 120 h and 240 h (*after start of infusion*) | |
| **Stage 2 (MAD)** | Day 1: | 0 h (*pre-dose*), 1 h (*during infusion*) and 2 h, 3 h, 4 h, 6 h, 10 h, 18 h, 24 h, and 36 h (*after start of infusion*) |
| | Day 3: | 0 h (pre-dose) |
| | Days 5, 7, 9, 11: | 0 h (*pre-dose*) and 2 h (*end of infusion*) |
| | Day 13 | 0 h (*pre-dose*), 1 h (*during infusion*) and 2 h, 4 h, 6 h, 10 h, 12 h, 18 h, 24 h, 36 h, 48 h, 72 h, 120 h and 240 h (*after start of infusion*) |

*Study drug is administered on Days 3, 5, 7, 9 and 11

The time of the start of infusion will be defined as study time Day 1, 0 h.

The deviations from scheduled sampling times which are considered to require specific comments in the comment section of the appropriate CRF page are specified in Table 11.

**Table 11: Time deviations requiring CRF comment**

| Time period after dosing | Comment in CRF is needed when deviation is more than: |
|---|---|
| 0h < scheduled time ≤ 4h | ±2 min |
| 4h < scheduled time ≤ 24h | ±10 min |
| scheduled time > 24h | ±60 min |

All pharmacokinetic calculations will be based on the actual times of the collection intervals as recorded.

Urine Collection (Stage 1 [SAD] only):
Urine sample collection for eventual PK purposes will be taken and stored for analysis during the SAD-study only.
The collection intervals will be as follows:

Sample 1: 0 h (pre-dose)
Sample 2: 0 h to 24 h
Sample 3: 24 h
Sample 4: 48 h
Sample 5: 72 h

Some 10 minutes before the specified end of each collection interval, the subjects will be sent to the toilet in order to void their bladders. This time will be noted on the CRF as the end of the collection interval and, if applicable, considered as the starting time of the next collection interval. If a subject should report that he was not able to void, the subject will be asked to give the approximate time of his last voiding, and this time will be recorded.

#### 9.3.1.3 Sample Handling Procedures

<u>Blood Samples:</u>
Each sample will be labeled to indicate the study number, subject number, period number (Stage 2 only), and sampling time.
Samples will be centrifuged for 10 minutes at 2000 g at room temperature. The plasma will be transferred to polypropylene storage tubes by pipetting, and deep-frozen at a temperature of -75°C (tolerance + 5°C) or lower (see Laboratory Manual for full details of sample handling).

<u>Urine Samples:</u>
*Stage 1 (SAD) only*
The total volume of the urine collected within an interval will be measured by weighing (1 g is assumed to equal 1 mL). Either the total urine, if less than 10 mL, or a 10 mL aliquot will be transferred to a smaller container and stored at -20°C (tolerance ± 5°C) or lower until the time of analysis.

All sample handling procedures, including the time of each sample collection, the time of placement into frozen storage (at the end of the sample workup), and the date of transfer or shipment of the samples to the responsible analyst will be documented in detail.
All samples will be stored for a period of six months after submission of the final report to the sponsor. After this period Nuvisan will ask the Sponsor if any further storage is required. If no separate contract for further storage has been agreed upon by the sponsor and Nuvisan, the samples will then be destroyed.

#### 9.3.1.4 Appropriateness and Description of Analytical Method(s)

The concentrations of PRS-080#022-DP in plasma will be determined by means of a validated method. A separate analytical study plan will be provided before the start of the analytical part of the study. Characteristics of the analytical method will be described in the final report.

#### 9.3.1.5 Blood Collection for Pharmacokinetic Assessment

The total blood draw per subject for pharmacokinetic assessment will vary for subjects according to the Stage of the study they are enrolled in, and will be follows:
Stage 1 (SAD): 14 samples (4.9 mL per sample), Total volume = 68.6 mL
Stage 2 (MAD): 33 samples (4.9 mL per sample), Total volume = 161.7 mL

### 9.3.2 PHARMACOKINETIC VARIABLES

#### 9.3.2.1 Description of the Pharmacokinetic Parameters

The following pharmacokinetic parameters will be calculated from measured plasma concentrations for each Stage, each subject, each substance analyzed, and each treatment:

Plasma:

| | | |
|---|---|---|
| $C_{max}$ | = | measured maximal concentration |
| $t_{max}$ | = | time of observed maximum concentration |
| $\lambda_z$ | = | terminal rate constant |
| $AUC_{0-t}$ | = | area under the concentration time curve (time 0 to last sample with a quantifiable concentration) |
| $AUC_{0-\infty}$ [a] | = | area under the concentration time curve from time 0 extrapolated to infinity |
| $AUC_{0-\tau}$ [b] | = | area under the concentration time curve over a dosing interval at steady state |
| MRT | = | mean residence time |
| $t_{½}$ | = | terminal half-life, from $\lambda_z$ |

a: $AUC_{0-\infty}$ will only be calculated in subjects treated in the SAD study and for the first dose of subjects treated in the MAD study.
b: $AUC_{0-\tau}$ will only be calculated for last dose of subjects treated in the MAD study.

#### 9.3.2.2 Methods for Derivation of Pharmacokinetic Parameters

Pharmacokinetic parameters will be calculated by so-called non-compartmental or model-free methods, e.g. linear trapezoidal rule for AUC, log-linear regression for $\lambda_z$ etc.(13).

The value of $AUC_{0-\infty}$ will be considered unreliable if the terminal area beyond the last quantified sample is greater than 20% of the total $AUC_{0-\infty}$, but will be reported nonetheless.

#### 9.3.2.3 Appropriateness of the Pharmacokinetic Parameters

The AUC is used to describe the extent of bioavailability. The rate of bioavailability is characterized by $C_{max}$ and $t_{max}$. $\lambda_z$ and $t_{½}$ describe the kinetics in the terminal phase which, for many substances, is governed by elimination processes.

## 9.4 SAFETY VARIABLES

### 9.4.1 DESCRIPTION OF THE SAFETY VARIABLES

The safety variables to be assessed in the present study are adverse events, local tolerability, vital signs (blood pressure and pulse rate), ECG recordings, body temperature (ear), respiratory rate, laboratory tests, and immunogenicity.

#### 9.4.1.1 Laboratory Assessments

Laboratory assessments will include determination of the following parameters.

Hematology:
Hematocrit (HCT), hemoglobin (Hb), red blood count (RBC), mean corpuscular volume (MCV), mean corpuscular hemoglobin (MCH), mean corpuscular hemoglobin concentration (MCHC), white blood count (WBC, leukocytes), platelet blood count (PBC, thrombocytes); reticulocytes, differential blood count (automated: neutrophil leukocytes [NEUT, granulocytes], eosinophil leukocytes [EOS, eosinophil granulocytes], basophil leukocytes [BASO, basophil granulocytes], lymphocytes [LYMPH], monocytes [MONO]).

Coagulation:
Prothrombin time (QUICK INR), activated partial thromboplastin-time test (APTT)

Specific Proteins:
Specific proteins: ferritin (FERR), transferrin (TRSF).

Electrolytes:
Sodium (Na), potassium (K), Chloride (Cl) and iron

Enzymes:
Aspartate-amino-transferase (AST/GOT), alanine-amino-transferase (ALT/GPT), alkaline phosphatase (AP), gamma-glutamyl-transferase (G-GT), Cholinesterase (CHE), lactate dehydrogenase (LDH), creatine-phosphokinase (CK), amylase

Substrates:
Glucose (GLUC), cholesterol (CHOL), blood urea nitrogen/urea (BUN/UREA), creatinine (CREA), total bilirubin (T-BILI), calcium (CA), Triglyceride (TG), uric acid (URIC), total protein (TP) albumin (ALB), globulin (GLOB), hs CRP

Urinalysis:
Nitrites* (NITRIT), leukocytes* (LEUCO), protein (PROT, albumin), glucose (GLUC), ketones (KETON), urobilinogen (UROBIL), bilirubin (BILI), blood* (ERY), negative common logarithm of hydrogen ion concentration (pH), specific weight (SP-WEIGHT)

*In case of clinically significant findings an investigation of the urine sediment will be performed.

<u>Exclusion Tests:</u>
*Serology*: human immunodeficiency virus antibodies 1/2 (HIV-AB, measured in serum), hepatitis B surface antigen (HBs-AG, measured in serum), hepatitis C virus antibodies (HCV-AB)
*Drugs*: cannabinoids (CANNAB, measured in urine), amphetamines (AMPH, measured in urine), barbiturates (BARB, measured in urine), benzodiazepines (BENZO, measured in urine), cocaine (COCAINE, measured in urine), opiates (OPIATE, measured in urine)
Ethanol (ALC, measured with Alkomat, breath test).

The total blood draw per subject for safety assessment will vary for patients according to the Stage of the study they are enrolled in, and will be follows:
Stage 1 (SAD): 67.1 mL
Stage 2 (MAD): 107.8 mL

### 9.4.1.2 Immunogenicity

Assessment of immunogenicity will be based on measurement of anti-drug antibodies (ADA).

## 9.4.2 TIME POINTS/INTERVALS FOR MEASURING THE SAFETY VARIABLES

<u>Vital signs, ECG and Respiratory rate:</u>
Vital signs (blood pressure and pulse rate), ECG, and respiratory rate will be measured (after 5 min in a supine position) during the screening examination, at regular intervals during the subject's hospitalization period (see Table 12), in the ambulatory visit conducted 120 h after study drug administration, and during the follow-up examination conducted 240 hours after initiation of study drug administration.

**Table 12: Timing of vital signs, respiratory rate and ECG assessments during hospitalization**

| Study | Time points for vital signs, respiratory rate and ECG assessments (relative to start of infusion)* | |
|---|---|---|
| **Stage 1 (SAD)** | ECG 0 h (*pre-dose*), 0.5 h and 1 h (*during infusion*) and 2 h, 4 h, 8 h, 12 h, 24 h, 48 h, and 72 h (after start of *infusion*) | |
| | Vital signs: (*pre-dose*), 0.25 h, 0.5 h, 0.75 h, 1 h and 1.25 h (*during infusion*) and 1.5 h, 1.75 h, 2 h, 2.5 h, 3 h. 3.5 h, 4 h, 8 h, 12 h, 24 h, 48 h, and 72 h (*after start of infusion*) | |
| **Stage 2 (MAD)** | Day 1: | 0 h (*pre-dose*), 0.5 h and 1 h (*during infusion*) and 2 h, 4 h, 8 h, 12 h, and 24 h (after start of) |
| | Days 3-11** | 0 h (*pre-dose*), 1 h (*during infusion*) and 2 h, 4 h and 24 h (after start of) |
| | Day 13 | 0 h (*pre-dose*), 1 h (*during infusion*) and 2 h, 4 h, 24 h, 48 h, and 72 h (after start of) |

* Time deviations (tolerance): pre-dose: +/ - 120min; 0.25h to 2h: +/- 5min; 2h to 72h: +/ - 15 min.
**Study drug is administered on Days 3, 5, 7, 9 and 11

<u>Body temperature</u>

Body temperature will be determined using an ear thermometer, during the screening examination, at regular intervals throughout the subjects' hospitalization period (see Table 13), in the ambulatory visit conducted 120 h after study drug administration, and during the follow-up examination conducted 240 hours after initiation of study drug administration.

**Table 13: Timing of temperature assessments during hospitalization**

| Study | Time points for temperature assessments (relative to start of infusion)* | |
|---|---|---|
| **Stage 1 (SAD)** | 0 h (*pre-dose*), 0.25 h, 0.5 h, 0.75 h, 1 h, 1.25 h, 1.5 h and 1.75 h (*during infusion*) and 2 h, 2.5 h, 3 h, 3.5 h, 4 h, 8 h, 12 h, 24 h, 48 h, and 72 h (after start of *infusion*) | |
| **Stage 2 (MAD)** | Day 1: | 0 h (*pre-dose*), 0.25 h, 0.5 h, 0.75 h, 1 h, 1.25 h, 1.5 h and 1.75 h (*during infusion*) and 2 h, 4 h, 8 h, 12 h, and 24 h (after start of) |
| | Days 3-11** | 0 h (*pre-dose*), 1 h (*during infusion*) and 2 h, 4 h and 24 h (after start of) |
| | Day 13 | 0 h (*pre-dose*), 1 h (*during infusion*) and 2 h, 4 h, 24 h, 48 h, and 72 h (after start of) |

* Time deviations (tolerance): pre-dose: +/ - 120min; 0.25h to 2h: +/- 5min; 2h to 72h: +/ - 15 min.
**Study drug is administered on Days 3, 5, 7, 9 and 11

Laboratory tests:
Blood and urine sampling for laboratory tests, including biochemistry, hematology, coagulation and urinalysis, will be performed during the screening examination, in the morning of Day -1, at regular intervals during the subject's hospitalization period (see Table 14), in the ambulatory visit conducted 120 h after the last study drug administration, and during the follow-up examination (240 h post-dose).
During the MAD study (Stage 2), samples will be collected for evaluation of hematocrit and hemoglobin, to allow assessment of stopping rules (see Section 7.3.1).

**Table 14: Timing of laboratory assessments during hospitalization**

| Study | Time points for laboratory assessments (relative to start of infusion) | |
|---|---|---|
| **Stage 1 (SAD)** | Day -1, then 24 h and 72 h post dose | |
| **Stage 2 (MAD)** | Day -1: | *pre-dose* |
| | Days 3, 5, 9, 11*: | 24 h prior to dosing, i.e. collection on Days 2, 4, and 8, respectively |
| | Days 7 and 11*: | 24 h prior to dosing, i.e. collection on Days 6 and 10, respectively: **haematology assessment only** |
| | Day 13 | 24 h prior to dosing (i.e. collection on Day 12) and 24 h and 72 h after dosing (i.e. collection on Days 14 and 16, respectively) |

*Study drug is administered on Days 3, 5, 7, 9 and 11

Since vital sign measurements, temperature, respiratory rate, ECG recordings and laboratory tests will be performed for safety reasons only, deviations from the planned time schedule are not relevant.

Adverse event questioning and concomitant medication:

Subjects will be interviewed by trained staff of Nuvisan GmbH to elicit information on possible adverse events at specific time points at screening, on Day -1, and on each day of hospitalization (see Table 15); such interviews will also be conducted at the ambulatory visit conducted 120 h after initiation of study drug administration, during the follow-up examination conducted 240 hours after initiation of study drug administration, and at the additional visit conducted on Day 28.

In case of adverse events, the investigator might decide to perform/repeat a clinical examination and/or any laboratory tests.

Any concomitant medications required by the subjects will be recorded at the same time points (see Table 15).

**Table 15: Timing of adverse event questioning and concomitant medication during hospitalization**

| **Study** | **Time points for adverse event questioning and concomitant medication (relative to start of infusion)*** | |
|---|---|---|
| **Stage 1 (SAD)** | 0 h (*pre-dose*), 0.25 h, 0.5 h, 0.75 h, 1 h and 1.25 h (*during infusion*) and 2 h, 2.5 h, 3.0 h, 3.5 h, 4 h, 8 h, 12 h, 24 h, 48 h, and 72 h (after start of *infusion*) | |
| **Stage 2 (MAD)** | Day 1: | 0 h (*pre-dose*), 0.5 h and 1 h (*during infusion*) and 2 h, 4 h, 8 h, 12 h, and 24 h (after start of *infusion*) |
| | Days 3-11** | 0 h (*pre-dose*), and 1 h, 2 h, 4 h and 24 h (after start of *infusion*) |
| | Day 13 | 0 h (*pre-dose*) and 1 h, 2 h, 4 h, 24 h, 48 h, and 72 h (after start of -*infusion*) |

* Time deviations (tolerance): pre-dose: +/ - 120min; 0.25h to 2h: +/- 5min; 2h to 72h: +/ - 15 min.
**Study drug is administered on Days 3, 5, 7, 9 and 11

Local tolerability

Local tolerability will be determined at regular intervals throughout the subjects' hospitalization period (see Table 16); additional assessment will be performed at the ambulatory visit conducted 120 h after initiation of study drug administration (Stage 1), and during the follow-up examination conducted 240 hours after initiation of study drug administration. Local tolerability at the injection site will be evaluated by the investigator using the ISR Score (see Table 17).

**Table 16: Timing of local tolerability assessments during hospitalization**

| **Study** | **Time points for local tolerability assessments (relative to start of infusion)*** | |
|---|---|---|
| **Stage 1 (SAD)** | 0 h (*pre-dose*), 0.5 h and 1 h (*during infusion*) and 2 h, 4 h, 8 h, 12 h, 24 h, 48 h, 72 h, 120h and 240h (after start of *infusion*) | |
| **Stage 2 (MAD)** | Day 1: | 0 h (*pre-dose*), 1 h (*during infusion*) and 4 h, 12 h, and 24 h (after start of *infusion*) |
| | Days 3-11** | 0 h (*pre-dose*) and 4 h (after start of *-infusion*) |
| | Day 13 | 0 h (*pre-dose*) and 4 h, 12 h, and 24 h and 240h (after start of *-infusion*) |

* Time deviations (tolerance): pre-dose: +/ - 120min; 0.25h to 2h: +/- 5min; 2h to 72h: +/ - 15 min.
**Study drug is administered on Days 3, 5, 7, 9 and 11

*Injection site reaction (ISR) score*
The injection site reaction assessment will be done by the investigator. It consists of rating the severity of each reaction for the injection site. The severity rating for injection site reactions is defined as provided in Table 17 below.

**Table 17: Injection site reaction score**

| | | |
|---|---|---|
| None | 0 | No reaction |
| Mild | 1 | Easily tolerated erythema and/or light bruising and/or mild pain |
| Moderate | 2 | Distributing erythema with swelling and/or disturbing bruising and/or disturbing pain (discomfort with movements) |
| Severe | 3 | Almost intolerable symptoms (significant discomfort at rest), or clinically definite skin necrosis, characterized by any of the following: oozing, weeping, skin breakdown, ulceration, scar formation |

Any reaction will be documented as an AE.
In addition, bleeding or fluid loss at the injection site will be documented. The diameter of any erythema or swelling will be measured (mm) in the largest dimensions.

Immunogenicity
Assessment of immunogenicity will be based on measurement of anti-drug antibodies (ADA). Blood samples for the determination of plasma/serum concentrations of ADA will be on Day 1, 0 h (pre-dose) and at the final visit, to be conducted 28 days after the initiation of study drug administration or premature discontinuation visit.
Blood samples for analysis of anti-drug antibodies will be prepared using Serum-S-Monovettes® (Sarstedt, 5.5 mL).
Serum samples will be shipped to the Laboratory for evaluation in one batch after the study. Further information on sample handling, storage, and shipment will be provided to the Investigator in a lab manual.

### 9.4.3 APPROPRIATENESS AND DESCRIPTION OF MEASUREMENTS OF THE SAFETY VARIABLES

All safety measurements are performed according to standardized methods, which are widely used and generally recognized as reliable and accurate.

For eliciting and rating of adverse events please refer to Sections 10.5 and 10.6, respectively.

## 9.5 CHANGES IN LABORATORY PARAMETERS

After sampling, blood and urine samples will be worked up and analyzed in Nuvisan's clinical laboratory, all results will be judged by a physician individually and commented as follows:

- Values within the reference ranges will not be commented. A '*' representing the value will be plotted within the brackets representing the reference range.
- For values slightly outside the reference ranges without clinical relevance a '*' representing the value will be plotted outside the brackets representing the reference range.
- For values outside the reference ranges with major deviation and/or possible pathological relevance a '*' representing the value will be plotted outside the brackets representing the reference range. In addition, the respective parameter will be shaded.

For all findings with major deviation and/or possible pathological relevance, follow-up examinations will be carried out until the deviation returns to normal or the absence of pathological relevance can be confirmed. If a deviation considered clinically relevant has not returned to a normal or not clinically relevant value when it is checked during the screening laboratory tests, the subject will not be included in the study.

The investigator has to decide whether a laboratory abnormality represents an adverse event.

# 10 SAFETY ASPECTS

## 10.1 ADVERSE EVENTS (AE)

An adverse event (AE) is any untoward medical occurrence in a patient or clinical investigation subject administered a medicinal product, which does not necessarily have a causal relationship with this treatment. An adverse event can therefore be any unfavorable and unintended sign (including an abnormal laboratory finding), symptom, or disease temporally associated with the use of a medicinal product, whether or not considered related to the medicinal product (Article 2(m) of Directive 2001/20/EC).

Untoward medical experiences occurring during drug-free pre-treatment periods do not meet the above-mentioned definition of adverse event. Nevertheless, they have to be documented in the same way as adverse events.

During and following a subject's participation in this trial, the investigators has to ensure that adequate medical care is provided to a subject for any adverse events, including clinically significant laboratory values, related to the trial.
Any AE which remains unresolved after completion of the trial requires detailed evaluation and follow-up until the AE has been resolved or a reasonable explanation for its persistence is found.

The subjects will be provided with cards stating that they are participating in a clinical study, giving the investigational product, the duration of the study, the address and telephone numbers of Nuvisan. These cards are meant for the case that the subjects consult a physician during their study participation or for emergencies.

## 10.2 ADVERSE DRUG REACTIONS (ADR) AND UNEXPECTED ADVERSE DRUG REACTIONS

An adverse drug reaction (ADR) is

> An untoward and unintended response to an investigational medicinal product related to any dose administered (Article 2(n) of Directive 2001/20/EC). The definition implies a reasonable possibility of a causal relationship between the event and the IMP. This means that there are facts (evidence) or arguments to suggest a causal relationship. (EU Detailed Guidance CT-3 (2011))

An unexpected adverse reaction is an adverse reaction, the nature or severity of which is not consistent with the applicable product information (e.g. Investigator's Brochure for an unapproved investigational product).

## 10.3 SERIOUS ADVERSE EVENTS (SAE) AND SERIOUS ADVERSE DRUG REACTIONS (SAR)

A serious adverse event (SAE) is any untoward medical occurrence or effect that, at any dose:

- results in death,
- is life-threatening,
- requires hospitalization or prolongation of existing hospitalization,
- results in persistent or significant disability or incapacity,

or

- is a congenital anomaly or birth defect (Article 2(o) of Directive 2001/20/EC)

NOTE: The term "life-threatening" in the definition of "serious" refers to an event in which the subject was at risk of death at the time of the event; it does not refer to an event which, hypothetically, might have caused death if it were more severe.
Medical and scientific judgment should be exercised in deciding whether expedited reporting is appropriate in other situations, such as important medical events that may not be immediately life-threatening or result in death or hospitalization, but may jeopardize the subject or may require intervention to prevent one of the other outcomes listed in the definition above. These should usually also be considered serious.
Examples of such events are intensive treatment in an emergency room or at home for allergic bronchospasm; blood dyscrasias or convulsions that do not result in hospitalization; or development of drug dependency or drug abuse.

**Pregnancy:**
No females should participate in this study. If the female partner of a volunteer becomes pregnant within 3 months after IMP administration, the following applies:
Although not a serious AE in itself, exposure to study medication during pregnancy - even if no AE is produced in the mother - should be reported within 24 hours, and the pregnancy should be followed to outcome. Pregnancies should be reported on a pregnancy reporting form. However, if the pregnancy is associated with a serious adverse event (for example, if the mother is hospitalized for eclampsia) an SAE form must be completed and SAE reporting procedures are to be followed.

## 10.4 SUPECTED UNEXPECTED SERIOUS ADVERSE REACTION (SUSAR)

A SUSAR is a serious adverse reaction (SAR) that is unexpected observed during a clinical trial and for which there is a causal relationship with the investigational medicinal product, whether it is the tested drug or its comparator.

## 10.5 ELICITING, DOCUMENTATION AND REPORTING OF ADVERSE EVENTS

Information on adverse events will be derived by questioning the subjects in general terms (e.g. "How do you feel?" or "How have you been feeling since the last questioning?"), by subjects' spontaneous reports, or by observation.

Adverse events will be documented on special source data sheets in German language. The entries on the source data sheets will be checked by trained Nuvisan staff members. The adverse events will be transcribed and translated to English on special CRF-pages. The CRFs

will be transmitted to the "Clinical Data Management" Department who will enter the adverse events into a data base for the further evaluation. The following information will be given for each adverse event:

Description of the adverse event, start date, start time, stop date, stop time, severity, pattern, action taken, outcome, seriousness, expectation and possible relationship to the study drug.

## 10.6 RATING OF ADVERSE EVENTS

Rating of adverse events will be performed by the investigators.

The severity of adverse events is characterized as follows:

**mild:**
Any symptom of which the subject is aware, but which is easily tolerated;

**moderate:**
Any symptom which is discomforting enough to cause interference with a subject's usual activity;

**severe:**
Any symptom which causes a subject's inability to perform usual activity

Drug-Event Relationship

The causal relationship between the study drug and the AE should be characterized according to the following:
**Unrelated** – there is not a reasonable possibility that the study drug caused the AE.
**Unlikely** – suggests that only a remote connection exists between the study drug and the event. Other conditions, including concurrent illness, progression or expression of the disease state or reaction to concomitant medication, appear to explain the AE.
**Possible** – suggests that the association of the AE with the study drug is unknown, however the event is not reasonably supported by other conditions.
**Probable** – suggests that a reasonable temporal sequence of the AE with drug administration exists and, in the Investigator’s clinical judgment, it is likely that a causal relationship exists between the drug administration and the AE, and other conditions (concurrent illness, progression or expression of the disease state, or concomitant medication reactions) do not appear to explain the AE.
Events where a causal relationship is classified as probable or possible will be regarded as related.

Outcome
The outcome of the adverse event should be classified according to the following definitions:
**Recovered / resolved**: the event has resolved (no further symptoms are present and no treatment is being received by the subject).
**Recovered / resolved with sequelae**: the event has resolved but there may be lingering effects present (e.g., a scar following a cut or abrasion).

**Fatal:** the subject died as a result of the event. This code should only be used for the event that caused the death, not any event that was present at the time of the subject's death. Fatal events require immediately reporting to the Sponsor and the Sponsors QPPV provided by FGK .
**Unknown:** may only be used in the event that the subject is lost to follow-up and no reliable data can be obtained.

All efforts should be made to classify the AE according to the above categories.
Note: when the AE is ongoing, the outcome will remain blank on the Adverse Events form in the CRF.

## 10.7 DOCUMENTATION AND REPORTING OF IMMEDIATELY REPORTABLE ADVERSE EVENTS

Sponsor and Nuvisan acknowledge that FGK will provide the QP-Pv. The QP-Pv will be available for all safety issues 7 days per week. The Parties acknowledge that, amongst other things, the QP-Pv is responsible for the clinical data safety management in accordance with applicable regulations (e.g. the ICH guideline E2A) and thus, will keep detailed records of all AEs that are reported to him by the NUVISAN's Investigator(s).
NUVISAN will report all urgent safety measures implemented by the Investigator(s), any communication from an IEC or regulatory authority indicating a safety or compliance issue/question, any pharmaceutical quality defect on the IMP and any serious adverse events as well as all serious adverse reactions (expected or unexpected, e.g. all SAEs and SUSARs) immediately and directly to the QP-Pv at FGK **within 24h** by using the **Fax-S alert System** of FGK and to PIERIS by phone, e-mail or facsimile, except for those safety issues that the Protocol or the Investigator's Brochure (IB) identifies as not requiring immediate reporting. The Parties acknowledge that FGK will train the Investigators on how to use FGK's Fax-S alert System and the Investigators shall familiarize themselves with this Fax-S alert System. The immediate report should be followed promptly by detailed, written report to both, FGK and PIERIS. The immediate and follow-up reports shall identify subjects by unique code numbers assigned to the latter.

Any unexpected serious adverse event that could adversely affect the safety of the subjects or the conduct of the study and any serious adverse event, which occurs during the course of this study, will be reported immediately by the investigator(s) to the sponsor and its QPPV at FGK (i.e. within 24 hours). The information will comprise at least the following data:

- Name, address, and telephone number of the reporting investigator
- Investigational product(s)
- Study code
- Subject identification number, sex, and month and year of birth
- Description of the adverse event, measures taken and outcome (if resolved)
- Likelihood of drug causation of the adverse event assessed by the investigator

<u>Reports will be addressed to:</u>

| | |
|---|---|
| Name QPPV: | Edgar Fenzl |
| Address: | Heimeranstr. 35, 80339 Munich, Germany |
| Telephone: | +49 (0) 8989 3119-22 |
| Fax: | +49 (0) 89 893 119-20 |

| | |
|---|---|
| Fax-S alert System | +49 (0) 89 120 895 113 |
| | |
| Name sponsor representative: | Ulrich Moebius |
| Address: | Lise-Meitner-Straße 30<br>85354 Freising-Weihenstephan |
| Telephone: | 08161 1411 400 |
| Fax: | 08161 1411 444 |
| E-mail: | moebius@pieris-ag.com |

The sponsor ensures that all relevant information about suspected serious unexpected adverse reactions that are fatal or life threatening is recorded and reported as soon as possible to the Competent authorities in all the Member States concerned, and to the Ethics Committee, and in any case no later than seven days after knowledge by the sponsor of such a case, and that relevant follow up information is subsequently communicated within additional eight days.
The sponsor further ensures that all other suspected serious unexpected adverse reactions will be reported to the Competent Authorities concerned and to the Ethics Committees concerned as soon as possible but with a maximum of fifteen days of first knowledge.
The sponsor will inform all investigators about any event which necessitates reconsideration of the benefit-risk-ratio of the investigational drug.
The sponsor ensures that as soon as possible but no later than fifteen days after knowledge the Competent Authorities in all Member States concerned and the Ethics Committees will be informed about any event which necessitates reconsideration of the benefit-risk-ratio of the investigational drug. These events are in particular:

- single cases of expected serious adverse reactions with an unexpected outcome.
- an increased incidence of expected serious adverse reactions which is considered clinically relevant,
- suspected serious unexpected adverse reactions occurring after a concerned person has completed the study,
- events related to the conduct of the study or the development of the drugs possibly affecting the safety of the concerned persons.

All additional measures deemed necessary through new findings and taken by the sponsor or the investigator to protect the safety of the persons concerned and their triggering circumstances will be reported as soon as possible to the Competent Authorities concerned and the Ethics Committees.
Once a year throughout the clinical trial the sponsor will ensure that the Member States in whose territory the clinical trial is being conducted and the Ethics Committees are provided with a listing of all suspected serious adverse reactions which have occurred over this period and a report of the subject’s safety.

The safety reporting, including the decoding procedures, and the recall of IMP will be handled according to FGK’s SOPs.

In the event of a fatality, the "Study participant’s insurer" will be informed by Nuvisan GmbH within 24 hours after the fatality has come to Nuvisan GmbH's knowledge. In case of other serious adverse events, the "Study participants' insurer" has to be informed promptly.

## 10.8 WARNINGS AND PRECAUTIONS

General precautions include those related to protein-induced anaphylactic, infusion-related reactions of single individuals, which require emergency interventions by a physician including application of high dose corticosteroids, anti-histaminic drugs, volume resuscitation and catecholamine therapy as well as $O_2$-insufflation or general resuscitation in rare cases. As such, all testing facilities should have immediately available procedures to administer treatment for an anaphylactic response should it occur.

# 11 QUALITY ASSURANCE AND QUALITY CONTROL

To ensure compliance with GCP and regulatory requirements, a member of the sponsor's (or a designated CRO's) quality assurance unit may arrange to conduct an audit to assess the performance of the study at the study site and of the study documents originating there. The investigator/institution will be informed of the audit outcome.
In addition, inspections by regulatory health authority representatives and IEC(s)/IRB(s) are possible. The investigator should notify the sponsor immediately of any such inspection.
The investigator/institution agree to allow the auditor or inspector direct access to all relevant documents and allocate his/her time and the time of his/her staff to the auditor/inspector to discuss findings and any issues. Audits and inspections may occur at any time during or after completion of the study.

## 11.1 MONITORING

The monitoring in these studies is contracted to FGK. On-site monitoring will be performed before, during, and after the trial. The monitor will ensure that the trial is conducted, recorded, and reported in accordance with the protocol, SOPs, Good Clinical Practice, and the applicable regulatory requirements. The monitor will check the accuracy and completeness of the CRF entries, source documents, and other trial-related records against each other. The investigator will provide direct access to source data/documents for trial-related monitoring.
The monitor will follow written SOPs as well as those procedures that are specified by the sponsor or its contractor for monitoring a specific trial.
Further details on monitoring are defined in the QAg between Nuvisan and Sponsor.

## 11.2 AUDIT AND INSPECTIONS

Quality assurance and quality control systems are implemented and maintained using written standard operating procedures (SOPs) to ensure that the trial is conducted and data are generated, documented (recorded), and reported in compliance with the protocol, GCP, and the applicable regulatory requirement(s).
Quality control will be applied to each stage of data handling to ensure that all data are reliable and have been processed correctly.
A quality assurance audit may be conducted by the sponsor or its agent at any time during, or shortly after, the study. The investigator will permit an independent audit by an auditor mandated by Sponsor, after reasonable notice. The purpose of an audit is to confirm that the study is conducted as per protocol, GCP and applicable regulatory requirements, that the rights and well-being of the patients enrolled have been protected, and that the data relevant for the evaluation of the investigational product have been captured, processed and reported in compliance with the planned arrangements. The investigator will permit direct access to all study documents, drug accountability records, medical records and source data.
Further details on Audit and Inspections are defined in the QAg between Nuvisan and Sponsor.

# 12 DOCUMENTATION, RECORD ACCESS, AND RECORD KEEPING

## 12.1 DOCUMENTATION AT THE STUDY CENTER

All data relating to the study will be documented in the Case Report Form. This CRF is developed to record the data requested by the protocol. The investigator will ensure the accuracy, completeness, legibility, and timeliness of the data recorded in the CRFs. Any change or correction to a CRF will be dated, initialed, and explained (if necessary), and will not obscure the original entry.

Subjects' medical history, results of the physical examination and other clinically relevant findings, demographic data (except of weight and ethnic origin) and adverse events will be documented on special source data sheets and then transferred to the individual Case Report Forms. Laboratory parameters will be printed out from a database. These print-outs will be inserted into the CRF as source data. The original ECG recording sheets will be inserted into the CRFs, and the date of ECG recording will be transcribed to special sections on the CRFs.

All other data collected during the study will be documented directly in the CRFs and regarded as source data.

A subject's participation in a study will be documented on his subject’s recruitment card. All studies performed at Nuvisan GmbH which the subject has participated in to date are listed on this card.

At the beginning of the trial, a Nuvisan trial master file will be established at the Center for Human Pharmacology of Nuvisan GmbH. Nuvisan GmbH will maintain the trial documents as specified in the ICH Guideline of Good Clinical Practice (1) and as required by the applicable regulatory requirements. Nuvisan GmbH will take measures to prevent accidental or premature destruction of these documents.

Nuvisan GmbH will permit trial-related monitoring, audits, and regulatory inspection, providing direct access to source data/documents.

## 12.2 SUBJECTS' DATA AND DATA PROTECTION

To protect the subject's identity, a subject identification code will be assigned by the investigator to each trial subject and used in lieu of the subject's name when the investigator reports adverse events and/or other trial-related data. Personal information will be treated as confidential, but may need to be reviewed by authorized representatives of the sponsor (monitor and auditor, respectively) and regulatory authorities. The subject's consent to direct access to his original medical records for data verification purposes has to be obtained prior to a subject's participation in the trial.

The investigator must maintain a list of names and identifying information (e.g. date of birth, subject identification code, date of study enrolment) of all subjects enrolled in the trial. The subject identification code list will be kept by the investigator in the Nuvisan trial master file.

## 12.3 DATA MANAGEMENT/CODING

The data management will be performed by the Clinical Data Management Department of Nuvisan. The CRFs will be logged in, data entry will be performed and data will be checked

using computerized and manual means to identify problem fields. Queries will be issued, e.g. on missing data, inconsistencies, illegibility, illegal values and improperly corrected items (e.g. without initials, date of change or a clear reason for change). Answers to queries, which have to be signed by the investigator, will be implemented in the database.
Adverse events will be coded with the current version of Medical Dictionary for Regulatory Activities (MedDRA). Laboratory data will be checked against the laboratory normal ranges. Quality control on the data will be performed on an ongoing basis during the study.
Clean data sets will be provided to the statistician to perform the statistical analysis.
The Stage 1 (SAD) and Stage 2 (MAD) parts will be handled separately. For each of the two stages, a separate database will be set up. Accordingly, separate procedures e.g. regarding data validation, data review and database lock will also be performed for both stages. Following collection of data from the Day 28 assessments, database lock and unblinding for the SAD part will be performed prior to entering into the MAD part.

### 12.4 RECORD KEEPING / ARCHIVING

Essential documents of the clinical trial including the subject identification code list, source data and case report forms will be retained for 15 years after the completion or discontinuation of the trial at Nuvisan GmbH. These documents will be retained for a longer period, however, if required by the applicable regulatory requirements or if needed by the sponsor.
Details on record keeping and archiving are defined in the QAg between Nuvisan and Sponsor.

## 13 STATISTICAL EVALUATION

This protocol serves as an umbrella protocol for Stage 1 (the SAD part) and Stage 2 (the MAD part). For each of the two stages, a separate database will be set up. Accordingly, the database lock and the statistical evaluation will be performed for both stages separately. However, the conventions described in this section of the protocol will hold for both stages, as far as applicable.
Further details will be described in a Statistical Analysis Plan. It has not yet been finally fixed whether a combined Statistical Analysis Plan (SAP) will be drawn up or whether the Statistical Analysis Plan for the MAD part will be designed as a separate document or as an amendment to the SAP for the SAD part.

### 13.1 STATISTICAL HYPOTHESES

The two study stages described in this protocol form an exploratory investigation without any formal statistical hypotheses. All results will be interpreted descriptively only.

### 13.2 DETERMINATION OF SAMPLE SIZE

The sample size per cohort in this SAD and MAD study is representative of other first in human studies. Power calculations were not used to derive the sample size.
It is anticipated that the specified number of healthy subjects should complete the study in accordance with this protocol. An insufficient number of evaluable cases might impair the aims of the study.

## 13.3 STUDY SUBJECTS

### 13.3.1 STUDY POPULATIONS

The statistical analysis will be based on separate analysis populations, defined as follows:

| | |
|---|---|
| Safety Set: | All subjects who receive at least one dose of study. |
| Pharmacokinetic Set: | All subjects who are included in the Safety Set and who satisfactorily completed a pharmacokinetic blood sampling period without any major protocol violations which would render the data unreliable. |
| Pharmacodynamic Set: | All subjects who are included in the Safety Set and who satisfactorily completed a pharmacodynamic blood sampling period without any major protocol violations which would render the data unreliable. |

A period of pharmacokinetic/pharmacodynamic sampling will be considered to have been completed satisfactorily if no more than 3 consecutive or non-consecutive blood samples have been missed by negligence of the subject or the investigator, or have no available results, and provided that the pattern of missing points still allows calculation of pharmacokinetic/ pharmacodynamic parameters.

### 13.3.2 PROTOCOL DEVIATIONS

Important deviations from the protocol, such as deviations from inclusion and exclusion criteria, relevant deviations in sampling times or from the planned time schedule of safety assessments will be reported in the clinical study report.

If an unexpected important deviation from the study protocol occurs, the investigator will consult the sponsor to make a decision on how this deviation can be handled.

### 13.3.3 DEMOGRAPHIC AND OTHER BASELINE CHARACTERISTICS

Demographic variables and baseline physical characteristics (age, race, weight, height, BMI, smoking status) will be summarized in tabular form, also showing means and ranges.
Inclusion and exclusion criteria as well as medical history will be listed by subject.
Baseline values for hepcidin will be tabulated.

### 13.3.4 DECODING OF RANDOMIZATION

The study randomization code will be disclosed to the Department of Biostatistics not before closing of the database for clinical data and unblinding. Analytical data with obvious unblinding potential (i.e., especially pharmacokinetic concentration data) will not be provided by the analytical laboratories to the study team and to the Department of Biostatistics prior to unblinding.
Further information on breaking the blind is provided in Section 8.7.

## 13.4 EFFICACY EVALUATION

Not applicable to the present study.

## 13.5 PHARMACODYNAMIC EVALUATION

Pharmacodynamic parameters will be listed by treatment, subject and time point. Absolute values and changes from baseline will be summarized utilizing descriptive statistics by treatment and time point.

## 13.6 PHARMACOKINETIC EVALUATION

### 13.6.1 STATISTICAL/ANALYTICAL ISSUES

#### 13.6.1.1 Summary of Pharmacokinetic Evaluations

In order to achieve a better approximation to a normal distribution, pharmacokinetic parameters related to concentrations (AUC, $C_{max}$) will be logarithmically transformed before analysis.
Further parameters of interest that might be evaluated statistically will be logarithmically transformed in case they are more likely to be log-normally than normally distributed (e.g. CL, $\lambda_z$, $t_{½}$) (14).
Subjects for whom the available data is incomplete may be excluded from the calculations (see Section 13.6.1.4).
Results for other parameters not mentioned above will be displayed in tabular form without a formal statistical comparison between treatments.

#### 13.6.1.2 Evaluation of Steady-State

In the MAD stage of the study (Stage 2), blood sampling for the determination of trough levels of PRS-080 will be performed on Days 5, 7, 9, 11 and 13. These results will be evaluated descriptively in order to assess by visual inspection that steady-state has been reached.

#### 13.6.1.3 Adjustments for Covariates

Not applicable.

#### 13.6.1.4 Handling of Dropouts or Missing Data

All data will be used to their maximum possible extent, but without any imputations for missing data. If a subject has appreciable or critical data points missing, he should be excluded from analysis. However, if a subject is missing an inconsequential data point, e.g. final collection time sample lost due to tube breakage and preceding sample unquantifiably low, then his remaining data can still be used.

#### 13.6.1.5 Interim Analyses and Data Monitoring

For each of the two study stages (SAD and MAD parts), a separate database will be set up. Accordingly, the database lock and the statistical evaluation will be performed for both stages separately. Prior to entering into Stage 2 (the MAD stage), at least a selection of evaluations based upon the SAD part should be completed which will help the DEC and the sponsor to determine the dose levels and corresponding dosing schemes for the MAD stage.

#### 13.6.1.6 Multicenter Studies

Not applicable to the present study.

#### 13.6.1.7 Multiple Comparison/Multiplicity

Not applicable

#### 13.6.1.8 Examination of Subgroups

Not applicable to the present study.

### 13.6.2 TABULATION OF INDIVIDUAL DATA

Analytical measurements will be performed in the Analytical Center of Nuvisan. After release of the data by the responsible analyst, data are transferred for evaluation into SAS data sets using validated software modules.
The results of subject samples measured will be displayed in separate tables for each treatment, including descriptive statistics for concentrations at individual sampling times. Similar tables will be prepared for pharmacokinetic parameters.

### 13.6.3 DRUG DOSE, DRUG CONCENTRATION, AND RELATIONSHIPS TO PHARMACOKINETIC DATA

Depending on the final dose escalation scheme in Stage 1 (the actual number and scheme of dose escalation steps will depend on the outcomes per dose level), add-on evaluations on the dose-relationship of PK and PD parameters may be agreed and performed.

### 13.6.4 BY-SUBJECT DISPLAYS

For each subject, concentration-time curves will be plotted on a linear and log-linear scale simultaneously.

## 13.7 SAFETY EVALUATION

### 13.7.1 STATISTICAL/ANALYTICAL ISSUES

Safety data will be evaluated descriptively only.

#### 13.7.1.1 Adjustments for Covariates

There will be no adjustments for covariates.

#### 13.7.1.2 Handling of Dropouts or Missing Data

All safety data of subjects who received at least one of the investigational products will be included in the safety analysis. All data will be used to their largest possible extent without any attempt to impute or extrapolate missing data.

#### 13.7.1.3 Interim Analyses and Data Monitoring

Clinical safety and safety laboratory data 24 h and 72 h after the initiation of study drug infusion will be used by the DEC for making the decision to continue dosing within a given dose cohort (i.e. progress to the next sub-group), and to escalate the dose, respectively. These decisions will be taken based upon ongoing clinical summaries provided by the clinic rather than upon any statistical output.

Accumulating safety data will be inspected regularly for any changes that might constitute an adverse event or that might pose an unexpected hazard to the participants in the trial. In the case of an adverse event that is regarded as drug-related and serious, it must be decided whether the study may be continued or has to be terminated early.

For each of the two study stages (SAD and MAD parts), a separate database will be set up. Accordingly, the database lock and the statistical evaluation will be performed for both stages separately. Prior to entering into Stage 2 (the MAD stage), at least a selection of evaluations based upon the SAD part should be completed which will help the DEC and the sponsor to determine the dose levels and corresponding dosing schemes for the MAD stage.

#### 13.7.1.4 Multicenter Studies

Not applicable to the present study.

### 13.7.2 EXTENT OF EXPOSURE

All subjects will receive drugs at the pre-specified fixed dosages. Information relating to the extent of exposure is thus contained in the treatment labeling.

### 13.7.3 ADVERSE EVENTS (AEs)

#### 13.7.3.1 Display of Adverse Events

Adverse events (AEs) will be encoded using the MedDRA dictionary. Any concomitant medication will be encoded with the World Health Organization (WHO)-DD. When transcribing AE information from raw data, any AEs that continue but change in intensity will be considered as one and only one AE whereas a recurrent AE (e.g. a headache for a couple of hours each day) will be considered as several AEs.

#### 13.7.3.2 Analysis of Adverse Events

Frequency tables will be generated sorted by system organ class and preferred term in which the number and percentages of subjects with treatment-emergent AEs and frequency of the events are reported. Three sets of such summary tables by treatment will be presented: overall, by relationship to study medication and by severity.

#### 13.7.3.3 Listing of Adverse Events by Subject

A listing of adverse events according to the ICH Guidelines will be drawn up. This listing, at minimum, will contain a description of adverse events as to nature, severity, time and date of occurrence, interval since last dose of investigational drug, duration, treatment (if any), outcome and likelihood of drug causation. Separate sorted listings, e.g. sorted by body system, will be prepared.

### 13.7.4 DEATHS, OTHER SERIOUS ADVERSE EVENTS, AND OTHER SIGNIFICANT ADVERSE EVENTS

Deaths, other serious adverse events, and other significant adverse events will be reported in form of epicrisis. In case of deaths or other serious adverse events that evidently are not related to the subject's participation in the trial, e.g. traffic accident, the textual description of the adverse event may be shortened appropriately.

### 13.7.5 CLINICAL LABORATORY EVALUATION

Clinical laboratory examinations are performed during screening examinations in order to determine whether the subject is eligible for participation in the study. For all findings with major deviations and/or possible clinical relevance repeat examinations will be carried out until the deviation returns to the reference range or the absence of clinical relevance can be confirmed. Otherwise the subject will not be included in the study. Clinical laboratory examinations performed at the time of discharge are inspected for any relevant changes in health. If any such changes should occur, the investigator will decide whether they represent an adverse event which will be reported accordingly.
Clinical laboratory parameters will be listed in full with suitable flags for abnormal values (clinically irrelevant / relevant) and will be summarized utilizing descriptive statistics by treatment and time point. A separate listing will show all results considered possibly clinically relevant abnormal.

### 13.7.6 VITAL SIGNS, PHYSICAL FINDINGS, AND OTHER OBSERVATIONS RELATED TO SAFETY

Clinical data on blood pressure, pulse rate, respiratory rate and temperature collected in the course of the study will be listed and summarized.
Other clinical data derived in the course of the study documented on the Case Report Forms and entered into the database will be listed and summarized as appropriate. Any unusual finding will be commented upon in the report.

# 14 ADMINISTRATIVE PROCEDURES

## 14.1 AMENDMENTS TO THE PROTOCOL

Modifications to the valid protocol should be made as an amendment.
Substantial amendments will be signed by not less than the principal investigator and the sponsor and approved by the Competent Authority and/or the Ethics Committee depending on the affected changes prior to being implemented, unless the amendment is made to eliminate an immediate hazard to the clinical study subjects.

Amendments are regarded as substantial where they are likely to have a significant impact on:

- the safety or physical or mental integrity of the subjects
- the scientific value of the trial
- the conduct or management of the trial
- the quality or safety of any investigational medicinal product used in the trial

Non-substantial amendments (e.g. changes in telephone number, etc., changes in the list of name and functions on pages 2 and 3 except for the principal investigator, responsible investigator, sponsor, QPPV sponsor) will not require approval by the Competent Authority and the Ethics Committee unless requested by or submitted to the Ethics Committee or Competent Authority.

The Principal Investigator will not implement any deviation from, or changes to the protocol without agreement by the sponsor and prior review and documented approval/favorable opinion from the Competent Authority and Ethics Committee of an amendment regarded as substantial, except where necessary to eliminate immediate hazards to trial subjects, or when the changes involve only logistical or administrative aspects of the trial (e.g. change in monitor(s), change in telephone numbers).

## 14.2 CONFIDENTIALITY AND PUBLICATION

The investigators agree to keep strictly confidential all unpublished information and results concerning this trial. Unpublished information must not be published or disclosed without the sponsor's prior written approval.

## 14.3 REGISTRATION OF STUDY AND INVESTIGATORS

Before initiation of the study, Nuvisan GmbH will inform the Local Authority according to German Drug Law about the trial.

A notification about the start and end of the study should be forwarded by the Principal Investigator to the Local Authority according to German Drug Law.

## 14.4 INSURANCE, CONTRACTS, FINANCES

The sponsor shall ensure that only inactive ingredients well known in pharmaceutical science and released by the respective national authorities for use in drugs and/or foodstuffs for humans will be employed in the manufacture of the investigational products. The sponsor shall further ensure that the investigational products are manufactured according to all relevant laws, regulations and industrial standards, principles and guidelines of the European Union that are applicable to pharmaceutical and biological products, including, in particular, GMP.
Analytical methods employed by Nuvisan GmbH for sample measurements remain the property of Nuvisan GmbH. In case of the method being described in the documentation delivered, the sponsor also undertakes not to imitate it and to observe secrecy towards third parties. Any analytical methods transferred to Nuvisan from the Sponsor remain the property of the Sponsor, and may not be employed by Nuvisan outside the conduct of the present study or other studies conducted on behalf of the Sponsor.
In accordance with local law, Nuvisan GmbH has the responsibility for obtaining and maintaining **volunteer insurance policies** as defined in Article II of the Clinical Service Agreement. The investigator will inform the subjects about the procedures and conditions for compensation.
The terms and conditions of the insurance will be included in the investigator's trial master file.
In addition to the protocol, trial-related duties, functions and financial aspects will be specified in the contract between the sponsor and Nuvisan GmbH as well as any other parties involved in the clinical trial.

# 15 REPORTING

After study completion, an "integrated" full report will be prepared for each of the two studies, according to the notes for guidance on structure and content of clinical study reports (15).

# 16 REFERENCE LIST

1. ICH Topic E6: Guideline for good clinical practice. 1.5.96.Note for Guidance on Good Clinical Practice. CPMP/ICH/135/95, in , 1996

2. DIRECTIVE 2001/20/EC OF THE EUROPEAN PARLIAMENT AND OF THE COUNCIL of 4 April 2001 on the approximation of the laws, regulations and administrative provisions of the Member States relating to the implementation of good clinical practice in the conduct of clinical trials on medicinal products for human use, in, 2009

3. Arzneimittelgesetzes of 1976, as amended

4. GCP verordnung vom 9. August 2004 (BGBl I. S. 2081), die zuletzt durch Artikel 8 des Gesetzes vom 19. Oktober 2012 (BGBl. I S. 2192) geandert worden ist, in , 2012

5. Berufsordnung für die Ärzte in Bayern, in der aktuellen Fassung

6. Ganz T, Nemeth E: Hepcidin and iron homeostasis. Biochim Biophys Acta 1823:1434-1443, 2012

7. Ramos E, Kautz L, Rodriguez R, et al.: Evidence for distinct pathways of hepcidin regulation by acute and chronic iron loading in mice. Hepatology 53:1333-1341, 2011

8. Nemeth E, Valore EV, Territo M, et al.: Hepcidin, a putative mediator of anemia of inflammation, is a type II acute-phase protein. Blood 101:2461-2463, 2003

9. Sun CC, Vaja V, Babitt JL, et al.: Targeting the hepcidin-ferroportin axis to develop new treatment strategies for anemia of chronic disease and anemia of inflammation. Am J Hematol 87:392-400, 2012

10. PIERIS AG: Scientific Advice Briefing Book - PRD-080#022-DP, in , 2013

11. Mross K, Fischer R, Richly H, et al.: First in human phase I study of PRS-050 (Angiocal), a VEGF-A targeting anticalin, in patients with advanced solid tumors: Results of a dose escalation study. Mol Cancer Ther 10, 2011 (abstr)

12. Riecke K, Zollner S, Boyce M, et al.: Single and Repeated Dose First-in-Human Study with the Anti-Hepcidin Spiegelmer Nox-H94 . Blood 120, 2012 (abstr)

13. Cawello W: Parameters for Compartment-free Pharmacokinetics, in . Aachen, Shaker Verlag GmbH, 1999

14. Lacey LF, Keene ON, Pritchard JF, et al.: Common noncompartmental pharmacokinetic variables: are they normally or log-normally distributed? J Biopharm Stat 7:171-178, 1997

15. ICH Harmonised Tripartite Guideline E3: Structure and Content of Clinical Study Reports, in , 1995
