# Supplementary material for: First-in-human Phase I studies of PRS-080#22, a hepcidin antagonist, in healthy volunteers and patients with chronic kidney disease undergoing hemodialysis
Source: PLoS One. 2019 Mar 27;14(3):e0212023. doi: 10.1371/journal.pone.0212023 (PMC6436791; doi:10.1371/journal.pone.0212023)
Supplement: S2 Protocol — (PDF) [file pone.0212023.s002.pdf]

STUDY PROTOCOL

PHASE IB STUDY TO EVALUATE THE SAFETY, TOLERABILITY, PHARMACOKINETICS, AND PHARMACODYNAMICS OF THE HEPCIDIN ANTAGONIST PRS-080#022-DP IN ANEMIC CHRONIC KIDNEY DISEASE PATIENTS UNDERGOING HEMODIALYSIS

PCS_02_15

EudraCT No.: 2015-005005-37

Protocol no.:	PCS_02_15
Sponsor:	Pieris Pharmaceuticals GmbH<br>Lise-Meitner-Straße 30<br>85354 Freising-Weihenstephan<br>Germany
Coordinating investigator:	Prof. Dr med. Lutz Renders<br>Klinikum Rechts der Isar<br>Abteilung Nephrologie<br>Ismaninger Str. 22<br>München<br>Germany
Protocol version and date:	Final 3.0, 19-Jul-2016
Previous protocol version and date:	Final 2.0, 15-Apr-2016

This study protocol must be kept strictly confidential. Disclosure of the contents (in whole or part) to third parties is permissible only with written consent of Pieris Pharmaceuticals GmbH.

released for publication 08.Oct.2018

Revision History

Original Version 2.0, dated 15 April 2016

Version 3.0, dated 19 July 2016

The following changes were included in Version 3.0 compared to Version 2.0:

1. **The inclusion criterion hemoglobin concentration was changed from 9 - 11 g/dL to 9.0 – 12.0 g/dL.**

   The upper limit of the hemoglobin concentration was changed in order to facilitate recruitment of patients. Since a change in hemoglobin concentration is not a primary study objective and not a critical secondary endpoint measure, this change is without consequence to the overall study goal.

   The following sections were changed:

   Synopsis / Study Protocol, inclusion criteria:

   Hemoglobin (Hb) **9 – 11 g/dL** was changed to Hemoglobin (Hb) **9.0 – 12.0 g/dL**.

2. **The inclusion criterion transferrin saturation (TSAT) was changed from ≤ 30% to ≤ 40%.**

   The upper limit of transferrin saturation was changed in order to facilitate recruitment of patients. This change will not affect the ability to use transferrin saturation as a secondary parameter for pharmacodynamic activity as known from the previous Phase I study with PRS-080#022-DP in normal subjects. This change is without consequence to the overall study goal.

   The following sections were changed:

   Synopsis / Study Protocol, inclusion criteria:

   Transferrin saturation (TSAT) **≤ 30%** was changed to **≤ 40%**.

3. **The inclusion criterion hepcidin concentration was changed from 5 - 50 nmol/L to 5.0 – 75.0 nmol/L**

   The upper limit of the hepcidin concentration was changed in order to facilitate recruitment of patients. Since a change in hepcidin concentration is not a primary study objective and not a critical secondary endpoint measure, this change is without consequence to the overall study goal.

The following sections were changed:

Synopsis / Study Protocol, inclusion criteria:

Hepcidin **5 – 50 nmol/L** was changed to Hepcidin **5.0 – 75.0 nmol/L**.

4. **The starting point of the 16-day screening period was changed from the ICF signature to the first Visit. The duration of the screening period remained unchanged with 16 days.**

   This change was performed for organizational reasons. It allows to inform the patient about the study, and to have the ICF signed before the screening visit while retaining the full 16-day time period for screening and screening sample analysis.

   The following section was changed:

   Synopsis, study periods:

   "For each subject the study will start with a ≤ 16 days screening period starting from the **informed consent signature** and ending at enrolment (start of infusion of study medication)" was changed to "For each subject the study will start with a ≤ 16 days screening period starting from **the first study visit** and ending at enrolment (start of infusion of study medication)."

5. **Rescreening of patients who failed a screening initially was not excluded, but is now specifically described and allowed. Further, one reanalysis of hemoglobin concentration within the 16-day screening period is now allowed.**

   Rescreening of a previous screening failure is now allowed after the end of the screening period at a time point defined by the investigator and in agreement with the patient. Since the medical condition of the patients change over time, a patient who did not meet the inclusion and exclusion criteria at one time, may meet those after the medical condition has changed. Hemoglobin is observed over a period of time before screening to verify stable hemoglobin concentrations. One reanalysis is allowed in order to leverage natural fluctuations of this parameter.

   The following sections were changed:

   Study Protocol, subject withdrawal from study participation

   The following text was included: "A patient who failed screening can be screened again after a time period defined by the investigator and after re-signing the ICF."

Study Protocol, study schedule, study conduct:

The following text was included: "Hb concentration can be reassessed once within the 16-day screening period in case the investigator determines so. In this case, a patient is eligible for enrolment when the result of the second Hb determination complies with the inclusion criteria."

6. **Study schedule:**
   For study visits on Day 2, it is possible to perform collection of blood samples and assessment of vital sings and body temperature beetween **44 and 48 hours** after start of infusion (**before dialysis on Day 2**).

   Also, the collection of blood samples and assessment of vital sings and body temperature can be performed beetween **48 and 52 hours** after start of infusion (**after dialysis on Day 2**).

   Those changes were done to allow dialysis being performed in the morning of Day 0 before the infusion but in the further course of the study allow dialysis being performed early in the afternoon. In this case it would otherwise not be possible to keep the exact time points of 44 hours (before dialysis) and 48 hours (after dialysis) after infusion.

   This possibility is now reflected in section 12.4 and 12.7.

7. **Study schedule:**
   For study visits on Day 5, it is possible to perform collection of blood samples and assessment of vital sings and body temperature beetween **116 and 120 hours** after start of infusion (**before dialysis on Day 5**).

   This change was done to allow dialysis being performed in the morning of Day 0 before the infusion but in the further course of the study allow dialysis being performed early in the afternoon. In this case it would otherwise not be possible to keep the exact time point of 116 hours (before dialysis) after infusion.

   This possibility is now reflected in section 12.4 and 12.7.

8. **Time – windows:**

   The time-windows for visits Day 2 and Day 5 were extended from ±15 minutes to **±30 minutes before or after dialysis**. This change was performed for organizational reasons.

   This possibility is now reflected in section 12.7.

9. Clarification:

It was clarified that a patient who withdraws from the study after screening but before study drug administration will be treated as screening failure and will be replaced.

1 Synopsis

Study code

PRS_02_15

Title of the study

Phase Ib study to evaluate the safety, tolerability, pharmacokinetics, and pharmacodynamics of the hepcidin antagonist PRS-080#022-DP in anemic chronic kidney disease patients undergoing hemodialysis

Principal investigator(s) and study center(s)

It is expected to include at least 3 study centers in Germany.

Coordinating investigator:

Prof. Dr med. Lutz Renders
Klinikum Rechts der Isar
Abteilung Nephrologie
Ismaninger Str. 22
81675 München, Germany

Clinical phase: Ib

Study duration (planned):

It is planned to start the study with the first patient signing informed consent in March 2016. The recruitment period will be about 6 months with study completion (last patient out) in October 2016 and study end (data base lock) in December 2016.

Study periods

For each subject the study will start with a ≤ 16 days screening period starting from the first study visit and ending at enrolment (start of infusion of study medication). After the treatment Day 0 with a single infusion of study medication, a 28 days follow up period will follow ending with the termination visit.

Study objectives

Primary objectives

The primary objective of this study is to determine the safety and tolerability of a single administration of PRS-080#022-DP of 2 mg/kg, 4 mg/kg and 8 mg/kg body weight in anemic stage 5 Chronic Kidney Disease patients requiring hemodialysis.

Secondary objectives

The secondary objectives of this study are:

- to determine the pharmacokinetics of PRS-080#022-DP after single administration at 2 mg/kg, 4 mg/kg, and 8 mg/kg bodyweight.
- to evaluate the effect of PRS-080#022-DP on pharmacodynamic parameters after single administration at 2 mg/kg, 4 mg/kg, and 8 mg/kg body weight: serum iron, ferritin, transferrin saturation, hemoglobin, reticulocyte hemoglobin, and reticulocyte count.
- to evaluate the effect of PRS-080#022-DP on hepcidin plasma levels after single administration at 2 mg/kg, 4 mg/kg, and 8 mg/kg bodyweight.
- to collect data on immunogenicity after single PRS-080#022-DP administration at 2 mg/kg, 4 mg/kg, and 8 mg/kg bodyweight.

Methodology

This is a multi-center, randomized, double-blind, placebo-controlled, single ascending dose phase Ib study in anemic stage 5 chronic kidney disease (CKD) patients requiring hemodialysis.

Eligible subjects will undergo screening assessments and PRS-080#22-DP will be administered by slow infusion over 60 min using an infusion pump on Day 0. Subjects will be observed with regard to safety for at least 4 hours after end of infusion. Follow up visits will be performed at Day 1 (19 hours and 29 hours after start of infusion), Day 2 (44 hours and 48 hours after start of infusion), Day 3 (70 hours after start of infusion), Day 5 (116 hours after start of infusion), and at Day 7, Day 14, Day 21, and Day 28.

The study will consist of 3 dose cohorts of 2 mg/kg, 4 mg/kg, and 8 mg/kg body weight with 8 subjects in each cohort. Using a standard 6+2 design, 6 subjects in each cohort will be randomized to PRS-080#022-DP and 2 subjects in each cohort will be randomized to placebo.

The study is designed to provide maximum safety to the subjects using safety precautions within a cohort and between cohorts as described below.

Safety precautions within a cohort

In each dose cohort initially 2 subjects will be treated (1 subject will receive PRS-080#022-DP and the other subject will receive placebo). Once the 2 subjects have completed the assessments scheduled at 19 hours after the start of the infusion and all safety and pharmacodynamic data from this period are available, the investigator, Medical Monitor, independent physician and sponsor will review the data and decide whether or not enrollment in the respective cohort is continued. A further 6 subjects will only be enrolled in the cohort if

no safety concerns have been identified based on data from the first 2 subjects.

For each cohort (i.e., from the first PRS-080#022-DP administration in one cohort to the termination visit of the last subject of the same cohort) the following safety reassessment criterion applies:

- 1 or more subjects experience a treatment related SAE.

If the criterion is met:

- Randomization and administration of study medication is suspended until the respective investigator(s), independent physician, the Medical Monitor, and the sponsor will decide together on the continuation of randomization from a safety point of view.
- The follow-up for the subjects already treated continues.

Safety precautions between cohorts (DEC)
The decision to escalate dose will be made by a specially appointed Dose Escalation Committee (DEC). The DEC will provide recommendations about stopping, modifying or continuing the study. The DEC will meet in person or by telephone for cohort safety review to provide dose escalation recommendations.

The decision to escalate the dose will be made after the last subject in a cohort has completed Day 7 based on a review of safety, safety laboratory, and pharmacodynamic data.

The DEC will consist of:

- the medical monitor
- the sponsor
- the coordinating investigator
- and an independent qualified physician

Details on the DEC members, the roles, and responsibilities will be described in a DEC charter.

Treatments

PRS-080#022-DP

- Active ingredient: PRS-080#022-DP
- PBS pH 6.5 (20 mM NaH_2PO_4; 115 mM NaCl; pH6.5)
- 5 mL clear solution

- 8.1 mg/mL protein strength
- Administration by intravenous infusion for 60 min
- Single dose of 2 mg/kg, 4 mg/kg or 8 mg/kg body weight
- Storage: -20°C ± 5°C

PRS-080#022-DP-Placebo#001

- PBS pH 6.5 (20 mM NaH_2PO_4; 115 mM NaCl; pH6.5)
- 5 mL clear solution
- No active ingredient
- Administration by intravenous infusion for 60 min
- Storage: -20°C ± 5°C

Number of subjects (total and for each treatment) planned

The planned total sample size is 24 subjects with 6 subjects each receiving PRS-080#022-DP at 2 mg/kg, 4 mg/kg, or 8 mg/kg BW (total 18 subjects), and 6 subjects receiving placebo.

Subject population

Patients with anemic stage 5 CKD requiring hemodialysis

Inclusion criteria

Patients meeting the following criteria will be considered for inclusion into the study:

1. Patients with stage 5 CKD having been on hemodialysis for at least 90 days
2. Male and post-menopausal (≥ 12 months after the last menstruation) female patients of ≥ 18 years and ≤ 100 kg body weight
3. Patients being on stable (less than 30% change) erythropoiesis-stimulating agent (ESA) dose for 6 weeks prior to study medication administration
4. Hemoglobin (Hb) 9.0 – 12.0 g/dL with no changes greater than 1.5 g/dL over the last 6 weeks prior to study medication administration
5. Ferritin ≥ 300 ng/mL.
6. Transferrin saturation (TSAT) ≤ 40%
7. Hepcidin 5.0 – 75.0 nmol/L
8. Male patients with a female partner of childbearing potential agree to use a medically acceptable method of contraception (e.g. condoms, sexual abstinence, vasectomy),

not including the rhythm method for 30 days after administration of the study medication.

9. The patient is legally competent, has been informed of the nature, the scope and the relevance of the study, voluntarily agrees to participation and the study's provisions, and has duly signed the informed consent form (ICF). Subject agrees to comply with the protocol-mandated procedures and visits.

Exclusion criteria

A patient will not be eligible for inclusion if any of the following criteria applies:

1. Anemia due to causes other than CKD, including hemoglobinopathies, hemolytic anemias, myelodysplasia or malignancy
2. Blood transfusion within 2 months before administration of study medication.
3. Any iron treatment from 1 week before study medication administration until 1 week after study medication administration.
4. Previous enrollment in this study
5. Current or previous (within 60 days before study medication administration) treatment with another investigational drug and/or medical device or participation in another clinical study.
6. Pregnancy or breast-feeding women of child-bearing age.
7. Employees of the sponsor or subjects who are employees or relatives of the investigator
8. Known allergy to any component of the PRS-080#022-DP formulation
9. Positive for hepatitis B surface antigen (HBs Ag), anti-hepatitis C virus antibody (anti-HCV Ab), or human immunodeficiency virus (HIV)
10. Planned surgery during the study period
11. Unwilling or unable to comply with the protocol, in the judgment of the investigator
12. Unstable angina, myocardial infarction, percutaneous transluminal coronary angioplasty/stents, apoplex or coronary artery bypass grafting < 3 months prior screening.
13. Congestive heart failure: New York Heart Association Class III or IV.

14. Any medical condition that in the judgment of the investigator might interfere with study participation or jeopardize subject's safety during the study (e.g. active infection).

Previous and concomitant medication

Any iron treatment is not allowed from 7 days before study medication administration until 7 days after study medication administration. All other required treatments are allowed during the study period.

Criteria for evaluation

Efficacy

As this study is designed to evaluate the safety and tolerability of PRS-080#022-DP efficacy will not be assessed. The activity of PRS-080#022-DP will be measured by pharmacodynamic parameters.

Pharmacokinetics

The following pharmacokinetic parameters will be calculated for each subject from measured total and free PRS-080#22-DP plasma concentrations:

- C_{max} (measured maximal concentration)
- t_{max} (time of observed maximum concentration)
- λ_z (terminal rate constant)
- AUC_{0-t} (area under the concentration time curve from time 0 to last sample with a quantifiable concentration)
- $AUC_{0-\infty}$ (area under the concentration time curve from time 0 extrapolated to infinity)
- MRT (mean residence time)
- $t_{½}$ (terminal half-life, from $\lambda_{z'}$)
- V_{ss} (volume of distribution at steady state)

Pharmacodynamics

To assess the activity of PRS-080#022-DP, the following pharmacodynamic assessment will be done:

- Hepcidin plasma concentration
- Markers with fast response kinetics: iron, transferrin saturation and ferritin in blood serum

- Markers with slower response kinetics: hemoglobin, reticulocytes and reticulocyte hemoglobin in whole blood

Safety and tolerability

To assess safety and tolerability the following assessments will be performed:

- Adverse event:

  Pre-treatment emergent event, treatment-emergent adverse events, serious adverse events, adverse drug reactions, unexpected adverse drug reactions, suspected unexpected serious adverse reactions

- Laboratory:

  Hematology including hemoglobin, reticulocytes, and reticulocyte hemoglobin concentration, biochemistry, coagulation

- Vital signs, routine physical examination, electrocardiogram, and body temperature
- Immunogenicity: anti drug antibodies

Statistical methods

The planned analyses are of exploratory nature without any formal statistical hypotheses. All parameters will be descriptively analyzed using standard statistical methods. No formal statistical hypotheses are tested for this standard design dose escalating study. The sample size is considered being sufficient to evaluate the safety and tolerability in this study.

Schedule of assessments and flow chart

Flow chart 1

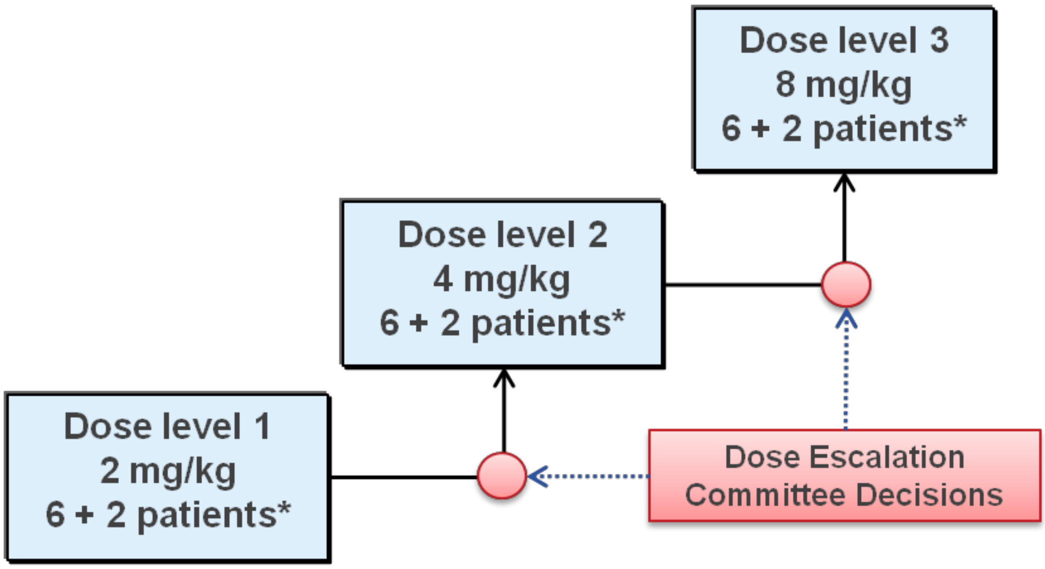

* 6 patients receiving PRS-080#22-DP, 2 patients receiving placebo per cohort

Flow chart 2

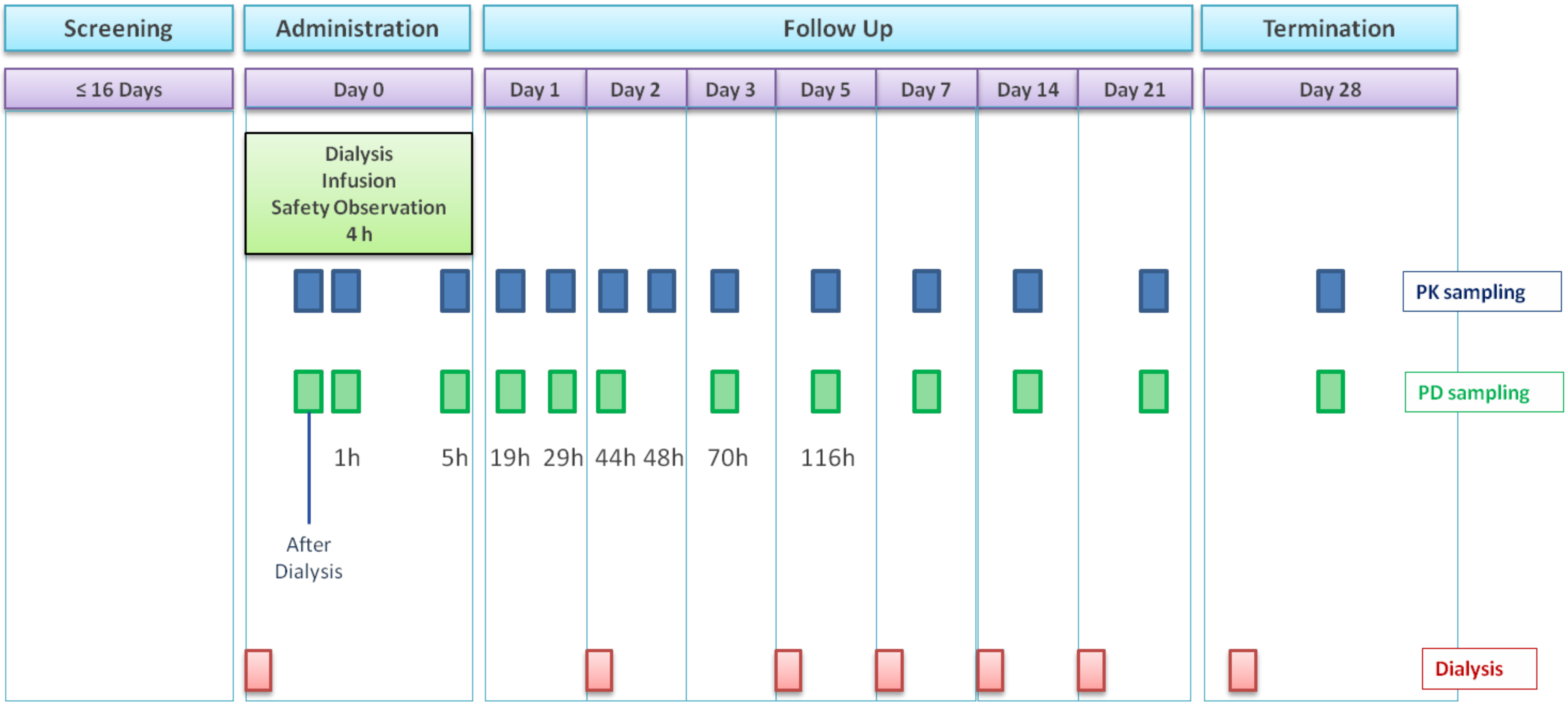
Screening
Administration
Follow Up
Termination
≤ 16 Days
Day 0
Day 1
Day 2
Day 3
Day 5
Day 7
Day 14
Day 21
Day 28
Dialysis
Infusion
Safety Observation
4 h
PK sampling
PD sampling
1h
5h
19h
29h
44h
48h
70h
116h
After
Dialysis
Dialysis

Table of procedures	Screening	Administration							Follow Up									
	≤ Day 16	Day 0						Day 1		Day 2		Day 3	Day 5	Day 7	Day 14	Day 21	Day 28 ± 2
		End of Dialysis	**Start of infusion**		End of Infusion									All visits at about the same time of the day as on Day 5[2]			
**Time after start of Infusion [hours]**			0[1]	0.5	1	3	5	19	29	44[2]	48[3]	70	116[2]				
Informed Consent procedure	x																
Demographic data	x																
Height, weight (BMI) for dose calculation	x																
Inclusion/exclusion criteria	x																
Changes in inclusion/exclusion criteria		x															
Medical history	x																
PTEE questioning		x															
TEAE questioning/observation (+ local tolerability)			x					x	x	x	x	x	x	x	x	x	x
Concomitant medication questioning /observation	x	x	x					x	x	x	x	x	x	x	x	x	x
Vital signs (blood pressure + pulse rate)	x	x		x		x	x	x		x		x	x	x	x	x	x
Body temperature (ear)	x	x		x		x	x	x		x		x	x	x	x	x	x
ECG (12-lead)	x																x
Physical examination	x							x		x				x	x	x	x
Safety Lab (BC, Hem)	x							x		x				x			x
Safety Lab Coagulation	x																x
Serology (HIV Ab, HBsAg, anti-HCV Ab)	x																
Blood pregnancy test	x																
Hepcidin - plasma	x	x			x		x	x	x	x	x	x	x	x	x	x	x
PD (Fe, TSAT, Ferritin) - serum	x	x			x		x	x	x	x		x	x	x	x	x	x
PD (Hb, Ret, RetHb) – WB	x							x		x				x			x
ADA - serum	x																x
Dialysis (not study specific)		x								x			x	x	x	x	x
Drug administration (Infusion 60 min)			x														
**PRS-080#022-DP PK (plasma)**		x			x		x	x	x	x	x	x	x	x	x	x	x

ADA: anti-drug antibody, BC: biochemistry, BMI: body mass index, ECG: electrocardiogram, Fe: iron, HBs Ag: hepatitis B surface antigen, Hb: hemoglobin, anti-HCV Ab: anti-hepatitis C virus antibody, Hem: hematology, HIV Ab: human immunodeficiency virus antibody, PD: pharmacodynamics, PK: pharmacokinetics, PTEE: pre-treatment emergent event, Ret: reticulocytes, RetHb: reticulocyte hemoglob TEAE: treatment emergent adverse event, TSAT: Transferrin saturation, WB: Whole Blood, [1]Within 2 hours after end of Dialysis, [2]Blood sampling before dialysis, [3]Blood sampling after dialysis

2 Addresses and responsibilities

**Sponsor's Responsible Medical Officer**	Dr med. Edgar Fenzl<br>FGK Clinical Research GmbH<br>Heimeranstr. 35<br>80339 München, Germany<br>Phone: +49 89 893119 22<br>Fax: +49 89 893119 20
**Sponsor's Managing Director**	Stephen S. Yoder, J.D.<br>Pieris Pharmaceuticals GmbH<br>Lise-Meitner-Str. 30<br>85354 Freising, Germany
**Coordinating Investigator (Leiter der Klinischen Prüfung, LKP according to German Drug Law)**	Prof. Dr med. Lutz Renders<br>Klinikum Rechts der Isar<br>Abteilung Nephrologie<br>Ismaninger Str. 22<br>81675 München, Germany<br>Phone: +49 (89) 4140 2231<br>Fax: +49 (89) 4140 7734
**Central Laboratory**	synlab pharma institute<br>Bayerstr. 53<br>80335 München
**Sponsor**	Pieris Pharmaceuticals GmbH<br>Lise-Meitner-Str. 30<br>85354 Freising, Germany<br>Phone: +49 8161 1411 440<br>Fax: +49 8161 1411 444

Project Management

FGK Clinical Research GmbH
Heimeranstrasse 35
80339 München
Germany
Phone: +49 89 893 119 0
Fax: +49 89 893 119 20

3 Table of contents

Page

REVISION HISTORY 2
1 SYNOPSIS 6
2 ADDRESSES AND RESPONSIBILITIES 16
3 TABLE OF CONTENTS 18
4 ABBREVIATIONS AND DEFINITION OF TERMS 22
5 INTRODUCTION 25
5.1 BACKGROUND 25
5.1.1 Anemia of chronic disease 25
5.1.2 Hepcidin 25
5.1.3 PRS-080#022-DP 26
5.1.4 Non-clinical data with PRS-080#022 (drug substance) 26
5.1.5 Clinical Data 27
5.2 RATIONALE 29
5.3 JUSTIFICATION FOR DOSES TO BE EVALUATED 29
6 INVESTIGATORS, STUDY ADMINISTRATIVE STRUCTURE, AND STUDY COMMITTEES 30
6.1 Study administrative structure 30
6.2 Drug escalation committee (DEC) 30
7 STUDY OBJECTIVES 31
8 STUDY DESIGN AND DESIGN RATIONALE 31
8.1 Overall study design 31
8.2 Safety precautions within a cohort 32
8.3 Safety precautions between cohorts (DEC) 32
8.4 Study design rationale 32
8.5 Risk/benefit assessment 33
9 SUBJECT SELECTION 34
9.1 Sample size 34
9.2 Inclusion criteria 34
9.3 Exclusion criteria 35
10 RANDOMIZATION, BLINDING AND UNBLINDING PROCEDURES 35

10.1 Blinding **35**
10.2 Subject identification **36**
10.3 Randomization **36**
11 TREATMENTS **37**
11.1 Study medication **37**
11.1.1 PRS-080#022-DP 37
11.1.2 Placebo 38
11.1.3 Description, packaging and labeling 38
11.1.4 Storage and stability 39
11.1.5 Treatment dose and administration 39
11.1.6 Infusion stopping criteria 39
11.1.7 Drug accountability and subject compliance 40
11.2 Previous and concomitant medication **40**
12 STUDY SCHEDULE **40**
12.1 Study conduct **40**
12.2 Screening/Baseline **41**
12.3 Treatment – Day 0 .... **41**
12.4 Follow-up **43**
12.5 Termination Visit – Day 28 .... **46**
12.6 Early termination **47**
12.7 Time-windows **47**
13 EFFICACY ASSESSMENTS **48**
14 PHARMACOKINETIC ASSESSMENTS **48**
14.1 Pharmacokinetic assessment **48**
14.2 Specimen collection and shipment **48**
14.3 Bioanalytical methods **49**
14.4 Pharmacokinetic parameters **49**
15 PHARMACODYNAMIC ASSESSMENTS **49**
16 SAFETY ASSESSMENTS **50**
16.1 Adverse events **50**
16.1.1 Definitions 50
16.1.2 Classification of adverse events 52

16.1.3 Documentation of adverse events 53
16.1.4 Documentations of serious adverse events 53
16.1.5 Follow-up of adverse events 54
16.2 Laboratory investigations 54
16.2.1 Hematology 54
16.2.2 Biochemistry 55
16.2.3 Coagulation 55
16.3 Vital signs, routine physical examinations and ECG 55
16.3.1 Documentation 55
16.3.2 Vital signs 55
16.3.3 Physical examination 55
16.3.4 12-lead electrocardiogram 56
16.4 Body temperature 56
16.5 Immunogenicity 56
17 BIOSTATISTICAL METHODS 56
17.1 Sample size calculation 56
17.2 Analysis sets and types of analyses 56
17.3 Analysis of study conduct and subject disposition 57
17.4 Patient demographics/other baseline characteristics 57
17.5 Extent of exposure 57
17.6 Analysis of the primary endpoints / Safety evaluation 57
17.6.1 Statistical and analytical issues 57
17.6.2 Adverse events 57
17.6.3 Clinical laboratory and other safety measures 58
17.6.4 Vital signs, body temperature, physical findings, and other observations related to safety 58
17.7 Analysis of secondary endpoints 58
17.7.1 Pharmacokinetic evaluation 58
17.7.1.1 Statistical and analytical issues 58
17.7.2 Pharmacodynamic evaluation 58
17.7.3 Immunogenicity 59
17.8 Interim analysis 59
18 SUBJECT WITHDRAWAL FROM STUDY PARTICIPATION 59

18.1 Subject withdrawal 59
18.2 Subject replacements 60
19 ETHICAL AND LEGAL REQUIREMENTS 60
19.1 Good clinical practice, general requirements, and study registration.. 60
19.2 Independent ethics committee or institutional review board........ 60
19.3 Subject information and consent procedure 60
19.4 Insurance coverage 61
19.5 Submission to authorities 61
19.6 Subject confidentiality 61
20 CRITERIA FOR PREMATURE TERMINATION OF THE TRIAL AND CRITERIA FOR PREMATURE CLOSING A STUDY CENTER........ 62
20.1 Premature termination of the clinical study........ 62
20.2 Premature termination of the clinical study at an individual center 62
21 STUDY PROTOCOL, DOCUMENTATION AND ARCHIVING OF DATA...... 63
21.1 Amendments to the protocol 63
21.2 Protocol deviations........ 64
21.3 Archiving of data........ 64
22 DATA COLLECTION, MONITORING AND QUALITY ASSURANCE........ 65
22.1 Data collection 65
22.2 Data management procedures 65
22.3 Monitoring procedures........ 66
22.4 Audits and inspections........ 66
22.5 Study report........ 66
22.6 Publications 67
23 STUDY PERIODS 67
24 REFERENCES........ 69
25 APPROVAL AND SIGNATURES........ 70

4 Abbreviations and definition of terms

ACD	Anemia of chronic disease
ADA	Anti-drug antibody
ADL	Activities of daily living
AE	Adverse event
ALB	Albumin
ALT	Alanine-amino-transferase
AP	Alkaline phosphatase
API	Active pharmaceutical ingredient
aPTT	Activated partial thromboplastin-time test
AST	Aspartate-amino-transferase
AUC	Area under the concentration time curve
AUC_{0-t}	Area under the concentration time curve (time 0 to last sample with a quantifiable concentration)
$AUC_{0-\infty}$	Area under the concentration time curve from time 0 extrapolated to infinity
BW	Body weight
CA	Calcium
CHOL	Cholesterol
CK	Creatine-phosphokinase
CKD	Chronic kidney disease
C_{max}	Maximal concentration
CRA	Clinical research associate
CREA	Creatinine
CRO	Clinical research organization
CRP	C reactive protein
DEC	Dose Escalation Committee
ECG	Electrocardiogram
eCRF	Electronic case report form
ESA	Erythropoietin stimulating agents
GGT	Gamma-glutamyl-transferase
GLP	Good laboratory practice
GLUC	Glucose
GMP	Good manufacturing practice
Hb	Hemoglobin
HBs Ag	Hepatitis B surface antigen
HCT	Hematocrit

HCV Ab	Hepatitis C virus antibody
HIV	Human immunodeficiency virus
Hs CRP	High sensitivity c reactive protein
ICF	Informed consent form
ICH	International Conference on Harmonization
IEC	Independent ethics committee
IFN-ɣ	Interferon gamma
IL	Interleukin
IWRS	Interactive Web Response System
K	Potassium
MCH	Mean corpuscular hemoglobin
MCHC	Mean corpuscular hemoglobin concentration
MCV	Mean corpuscular volume
MedDRA	Medical Dictionary for Regulatory Activities
MRT	Mean residence time
MTD	Maximum tolerated dose
Na	Sodium
NaCl	Sodium chloride
NCI-CTCAE	National (US) Cancer Institute-Common Toxicity Criteria for Adverse Events
NGAL	Neutrophil gelatinase-associated lipocalin
NOAEL	No observed adverse effect level
P	Phosphate
PBS	Phosphate buffered saline
PD	Pharmacodynamics
PEG	Polyethylene glycol
PK	Pharmacokinetics
PTEE	Pre-treatment-emergent events
RBC	Red blood cell count
SAE	Serious adverse event
SAP	Statistical analysis plan
SUSAR	Suspected unexpected serious adverse reaction
t½	Terminal half-life
T-BILI	Total bilirubin
TEAE	Treatment-emergent adverse event
t_{max}	Time of observed maximum concentration
TNF-α	Tumor necrosis factor alpha
TSAT	Transferrin saturation
Vss	Volume of distribution at steady state

WBC	White blood cell count
λ_z	Terminal rate constant

5 Introduction

5.1 BACKGROUND

5.1.1 Anemia of chronic disease

Anemia is frequently caused by iron deficiency and a subsequent impairment of hematopoiesis. In addition to iron loss (e.g., bleeding), it can result from insufficient uptake of iron, insufficient release of iron from the body's iron stores, or the combination of both. Anemia of chronic disease (ACD) is a hypo-proliferative chronic anemia that develops in response to systemic illness or inflammation. It is commonly associated with inflammatory diseases and long term infections (including human immunodeficiency virus [HIV] and hepatitis B or C), autoimmune disease (including Crohn's disease and rheumatoid arthritis), cancer (including lymphoma and Hodgkin's disease) and with chronic kidney disease. Patients with ACD may sustain a relatively normal level of function at significantly reduced hemoglobin (Hb) levels, but will suffer from symptoms of hypoxia, including fatigue, headache, dizziness, tinnitus, syncope, and chest pain.

As intestinal iron adsorption is inhibited in ACD, treating it with oral iron supplements is often not effective. Using erythropoiesis-stimulating agents (ESAs) such as epoietin alfa is also limited because of known side effects of ESAs and not infrequent resistance to these agents. In addition, the adequate iron stores needed for ESAs to function effectively are missing. Therefore, intravenous iron is being used increasingly.

The discovery and characterization of the hepatic hormone hepcidin A as a master regulator of iron metabolism has brought the use of i.v. iron into question and introduced a new target pathway for the treatment of ACD with the potential to address the limitations of current treatments.

5.1.2 Hepcidin

Hepcidin has been identified as a key systemic regulatory hormone of iron homeostasis. Mature hepcidin is a 25-amino acid peptide hormone primarily synthesized in hepatocytes. It regulates the absorption of dietary iron across the duodenum, iron recycling from senescent erythrocytes, and the recovery of iron from storage in hepatocytes and macrophages, pathways by which iron is made available for the synthesis of heme and Hb in bone marrow erythrocyte precursors (1). Hepcidin functions by binding to and initiating the internalization and degradation of ferroportin, a transmembrane channel for iron between the cell and the plasma and the only known cellular iron exporter, thus impairing iron transit from intracellular stores to plasma. Hepcidin levels are physiologically increased by a homeostatic feedback loop in response to elevated plasma iron concentrations and cellular iron stores (2).

However, many disorders of iron imbalance can be attributed to aberrant hepcidin production (3), in which hepcidin synthesis is increased despite normal or low iron levels. The resulting hepcidin-mediated intracellular iron retention and reduced availability of iron for erythropoiesis in these

conditions leads to forms of potentially severe "iron-restricted" anemia. As such pathologic elevation of hepcidin is commonly observed in infection and inflammation (4), as well as in patients with cancer and chronic kidney disease, elevated hepcidin is now commonly regarded as the likely root cause of the hypoferremia and iron-restricted erythropoiesis seen in ACD.

5.1.3 PRS-080#022-DP

PRS-080#022-DP belongs to a novel class of proprietary non-antibody scaffold therapeutic target-binding proteins called Anticalins® that are directly derived from human lipocalins. Lipocalins are low molecular weight proteins with functions in ligand binding and transport that are abundantly expressed in human tissues and body fluids. Anticalins have been generated against a variety of targets from naturally occurring lipocalins (e.g., tear lipocalin and neutrophil gelatinase-associated lipocalin (NGAL) using mutation and selection processes. PRS-080#022-DP is the second Anticalin that has entered clinical development. PRS-080#022-DP is directed against the human hepcidin protein and was selected via phage display technology. It is composed of the protein moiety which is derived from the human lipocalin NGAL and a polyethylene glycol (PEG30) moiety to extend the half-life of the protein in plasma. The drug substance, which is referred to here also as PRS-080#022, is principally identical to the drug product since it is only sterile filtered and filled to become the drug product PRS-080#022-DP.

PRS-080#022-DP is produced by bacterial expression in *E. coli*. The chosen expression system was optimized for efficient soluble expression in the cytoplasm. After cell harvest, PRS-080#022-DP is purified via two chromatographic steps. The highly pure intermediate obtained after these 2 steps is then subject to PEGylation. After a further chromatographic removal of free PEG the final active pharmaceutical ingredient (API), PRS-080#022-DP, is obtained in PBS pH 6.5. A concentration of 8.1mg/mL protein content is adjusted. No excipients are added. The final product PRS-080#022-DP is stored at -20 °C ± 5°C.

PRS-080#022-DP is reconstituted as sterile solution to be administered by slow intravenous infusion over a time period of up to 1 hour.

5.1.4 Non-clinical data with PRS-080#022 (drug substance)

PRS-080#022-DP binds with picomolar affinity and high specificity to its target human hepcidin A. A functional assay conducted in T-Rex-293 cells transfected with the human ferroportin and a green fluorescence-coding sequence showed that PRS-080#022 was capable of inhibiting the internalization and degradation of the FNP-GFP fusion protein with an IC50 of ~14 nM.

Proof of concept for PRS-080#022 has been obtained in *in vivo* studies conducted in Cynomolgus monkeys, wherein 4 mg/kg PRS-080#022 produced a robust, transient and reversible increase in total iron levels from ~27 µM at baseline to 52 µM after 24 hours, with iron responses detectable at doses as low as 0.4 mg/kg (minimal effective dose). The maximal concentration (C_{max}) of the iron response was capped starting at 1.2mg/kg and did not increase at higher doses of up to 60mg/kg

also during repeat dose administrations. Pharmacokinetic (PK) properties were assessed in three preclinical species and used for allometric scaling to predict a human half-life of 50-60 hours.

A maximum tolerated dose (MTD) study was performed in Cynomolgus monkeys to establish the tolerability of PRS-080#022 in the relevant toxicology species after 30-minute infusion administrations. A single-dose phase with dose levels ranging from 8-60 mg/kg was followed by a repeat dose phase at 60 mg/kg with five administrations every second day. Overall, PRS-080#022 was well tolerated, with a no observed adverse effect level (NOAEL) of 60 mg/kg/dose for the IV route.

In the subsequent 4-week repeat-dose toxicity study in Cynomolgus monkeys, animals were treated every other day at 8, 24, or 48 mg/kg, IV PRS-080#022 showed good local and systemic tolerability in Cynomolgus monkeys and no mortality, no overt signs of toxicity, no late toxicity, and no effects on vital functions were noted during the treatment period or the 4-week treatment-free recovery period. In particular, there was no evidence of an adverse effect on the cardiovascular system or the respiratory system after treatment with PRS-080#022 in the GLP-compliant 4-week toxicology study in monkey. Clinical chemistry and hematology was unremarkable. Drug-specific anti-drug antibody (ADA) responses were detected in the majority of animals, but these antibodies did not neutralize the pharmacodynamic activity of PRS-080#022. No evidence of immunotoxicity was observed; especially there were no changes of cytokine or complement levels during the 4-week toxicity study in monkeys. Reproductive organs did not reveal any alterations. Microscopic examination showed hemosiderin in the periportal hepatocytes (visible with special stain only), consistent with the pharmacodynamic activity of PRS-080#022-, and additional findings that were consistent with PEG administration. The NOAEL in this study was determined as 48 mg/kg given every other day.

The systemic exposure of monkeys to free and total PRS-080#022-DP was generally independent of the gender. $AUC_{0-\tau}$ and C_{max} of free and total drug increased with the dose in an approximately dose-proportional manner across the 8 to 48 mg/kg dose range on Days 1, 27 and 29.

5.1.5 Clinical Data

A Phase I clinical study was completed in healthy male subjects. Single ascending doses of PRS-080#022-DP were administered at 0.08 mg/kg, 0.4 mg/kg, 1.2 mg/kg, 4 mg/kg, 8 mg/kg and 16 mg/kg in 250 ml 0.9% NaCl as infusion over 2 hours. The study was randomized, double-blind and placebo controlled. Six subjects per cohort received PRS-080#022-DP and two subjects per cohort received placebo (n=36 PRS-080#022-DP, n=12 placebo).

Single doses of PRS-080#022-DP were well tolerated by healthy subjects exposed to doses up to 16 mg/kg. The MTD has not been reached. The Drug Escalation Committee did not identify any events requiring termination of the study according to the pre-determined stopping criteria.

For dose groups 0.08 mg/kg, 0.4 mg/kg, and 1.2 mg/kg, there were no adverse events (AEs) at least possibly related to PRS-080#022-DP. In dose group 4.0 mg/kg, 2 AEs reported by 2 subjects treated with PRS-080#022-DP (i.e. mild injection site erythema and mild headache) were assessed as possibly related. In dose group 8.0 mg/kg, 2 AEs reported in 1 subject treated with PRS-080#022-DP (i.e. mild flushing and mild upper abdominal pressure sensation, both lasting 8 minutes) were assessed as probably related. In the dose group of 16 mg/kg 1 AE, reported in 1 subject treated with PRS-080#022-DP (i.e. mild headache) was assessed as possibly related by the investigator.

There were no clinically relevant laboratory abnormalities reported as adverse events (AEs) that were judged related to PRS-080#022-DP. No clinically relevant changes of the hepatic transaminases and renal parameters were reported in subjects treated with PRS-080#022-DP. Increase of C-reactive protein (CRP) was observed in some subjects, mostly in temporal relationship to episodes of nasopharyngitis (common cold) and was judged unlikely or not related to PRS-080#022-DP.

Vital signs (blood pressure, pulse rate, respiratory rate), body temperature and electrocardiogram (ECG) recordings showed no clinically relevant changes after dosing. There were no relevant increases in body temperature (>1°C to baseline) during infusion.

In the dose range investigated, no major hypersensitivity or infusion related reactions were reported. In 1 subject exposed to the dose level of 8 mg/kg, only mild and short-lasting (8 minutes) flushing and upper abdominal pressure sensation were observed early during infusion.
The local tolerability of PRS-080#022-DP given as a 2-hour infusion was very good. In dose group 4.0 mg/kg mild injection site erythema was observed in 1 subject.
The clinical and laboratory safety data obtained in this study showed good systemic and local safety and tolerability of PRS-080#022-DP intravenously administered in healthy subjects. No safety issues and no signal for liver or kidney toxicity were detected during this study. The data support and justify further clinical investigation. No specific risks could be identified that may raise concerns to investigate safety and efficacy in the target population for PRS-080#022-DP treatment, i.e. patients with chronic kidney disease requiring hemodialysis and suffering from anemia, hyporesponsive to ESA therapy.

PRS-080#022-DP had a weak immunogenic potential; ADAs were detected on Day 28 in a total of 5 subjects, 1 of 6 subjects in the 4 mg/kg group and 2 of 6 subjects each in the 8 mg/kg and 16 mg/kg groups.

Pharmacokinetics of total PRS-080#022-DP (unbound and bound to hepcidin) was dose proportional following single i.v. administration. Exposure to free PRS-080#022-DP (unbound) increased slightly more than dose-proportional. Especially at lower doses, free PRS-080#022-DP plasma concentrations were lower than for total PRS-080#022-DP, compatible with binding of PRS-080#022-DP to its target hepcidin. Median t_{max} of free and total PRS-080#022-DP occurred between 2 and 3 hours after start of infusion. Geometric mean $t_{1/2}$ ranged from 38.9 to 61.6 hours

for free PRS-080#022-DP (0.4 mg/kg to 16 mg/kg groups) and was slightly longer for total PRS-080#022-DP (63.8 and 80.8 hours).

Hepcidin concentrations decreased below baseline for a period of 6 to 36 hours, depending on dose. Subsequently, a dose-dependent increase in mean hepcidin concentrations was observed at 72 hours after dosing (48 hours in the 1.2 mg/kg group) with mean increases compared to baseline of 7.982 nM after 1.2 mg/kg up to 42.037 nM after 16 mg/kg. No increase was observed in the lowest dose groups and after placebo.

PRS-080#022-DP demonstrated the expected pharmacodynamics activity. Serum iron concentration and transferrin saturation demonstrated a similar time course following PRS-080#022-DP administration. Both parameters increased after dosing in the 0.4 mg/kg to 16 mg/kg dose groups with peak values between 10 hours post-dose for the lower dose groups and at 48 to 120 hours post-dose for the higher dose groups (maximum mean increase compared to baseline of +15.90 µmol/L for iron and +27.0% for transferrin saturation in the 16 mg/kg group). The time period with elevated iron concentrations and transferrin saturation increased dose dependently in the 1.2 mg/kg to 16 mg/kg dose groups (24h to 120 h). Serum iron and transferrin saturation decreased after dosing in the 0.08 mg/kg and placebo groups. No consistent dose-related post-dose changes were observed for ferritin, reticulocyte (Ret) count as well as reticulocyte and erythrocyte hemoglobin. There was no significant change in interleukin (IL)-β, IL-6, interferon (IFN)-ɣ and tumor necrosis factor (TNF)-α serum concentrations compared to baseline.

5.2 RATIONALE

Elevated levels of hepcidin restrict iron availability to the reticulo-endothelial system and contribute to functional iron deficiency and anemia in patients with stage 5 chronic kidney disease undergoing hemodialysis. Antagonizing hepcidin with PRS-080#022-DP has the potential to improve iron availability and erythropoiesis. PRS-080#022-DP was well tolerated in a single ascending dose study in healthy subjects and demonstrated activity by means of increasing serum iron concentrations and transferrin saturation. Due to renal deficiency and elevated hepcidin levels in CKD5-hemodialsis patients compared to healthy subjects, the pharmacokinetics and activity of PRS-080#022-DP has to be evaluated in the intended patient population after single dose treatment. It is the intention of this single dose study to establish a dose range that is safe in CKD5-hemodialysis patients and that induces a serum iron response for a period of 1 to 3 days. Such doses are then planned to be tested in the same patient population at a once per week treatment schedule for their potential to affect hemoglobin levels over a period of 4 weeks.

5.3 JUSTIFICATION FOR DOSES TO BE EVALUATED

PRS-080#022-DP demonstrated dose proportional increases of C_{max} and AUC after single dose i.v. administration to healthy subjects at doses ranging from 0.08 mg/kg to 16 mg/kg and an elimination half-life of approximately 3 days. Consistent elevations of serum iron concentration and transferrin

saturation were observed at doses of 1.2 mg/kg to 16 mg/kg. Importantly, peak iron concentrations in individual subjects were comparable over the doses, however, the time period of elevated iron levels increased nearly proportional with the dose. Thus, elevated iron levels were observed for 18 hours, 48 hours, 72 hours and 120 hours at 1.2 mg/kg, 4 mg/kg, 8 mg/kg and 16 mg/kg, respectively. The doses of 2 mg/kg, 4 mg/kg and 8 mg/kg as intended in this current study protocol are within the dose range producing iron responses in healthy subjects over the intended time period of about 1 to 3 days. A more prolonged iron response is currently not intended. Continuous elevation of serum iron as provoked in nonclinical studies resulted in exaggerated pharmacological effects as seen by iron deposition in the liver. Thus, based on current data, the intended dose range is compatible with an intended pulse-like iron response in the course of the planned weekly treatment schedule. Importantly, the intended doses were well tolerated in healthy subjects and did not raise concerns for their investigation in the intended patient population.

6 Investigators, study administrative structure, and study committees

6.1 Study administrative structure

It is expected to include at least 3 study centers in Germany. The study will be conducted under the supervision of Prof. Dr Lutz Renders, Klinikum Rechts der Isar, München as Coordinating Investigator (Leiter der Klinischen Prüfung) according to German Drug Law.

The Sponsor will be responsible for the overall supervision and administration of the study. Regulatory submission to the competent authority and the ethics review board, medical monitoring, project management, site monitoring, handling of AE reporting, data management, statistical analysis, and medical writing services will be done by FGK Clinical Research GmbH (in the following referred to as sponsor’s designee).

Outside vendors will be used for the following functions:

- Site for reconstitution, preparation of infusion bags and distribution
- Laboratory for Hepcidin analytics
- Laboratory for pharmacokinetics of PRS-080#022-DP and ADA
- Laboratory for Safety Lab and pharmacodynamic analyses
- eCRF and Interactive Web Response System (IWRS)

6.2 Drug escalation committee (DEC)

The decision to escalate dose will be made by a specially appointed Dose Escalation Committee (DEC). The DEC will provide recommendations about stopping, modifying or continuing the study. The DEC will meet in person or by telephone for cohort safety review to provide dose escalation

recommendations.

The decision to escalate the dose will be made after the last subject in a cohort has completed Day 7 based on a review of safety, safety laboratory, and pharmacodynamic data.

The DEC will consist of:

- the medical monitor
- the sponsor
- the coordinating investigator
- and an independent qualified physician

Details on the DEC members, the roles, and responsibilities will be described in a DEC charter.

7 Study objectives

Primary objectives

The primary objective of this study is to determine the safety and tolerability of a single administration of PRS-080#022-DP of 2 mg/kg, 4 mg/kg and 8 mg/kg body weight (BW) in anemic stage 5 Chronic Kidney Disease patients requiring hemodialysis.

Secondary objectives

The secondary objectives of this study are:

- to determine the pharmacokinetics of PRS-080#022-DP after single administration at 2 mg/kg, 4 mg/kg, and 8 mg/kg BW.
- to evaluate the effect of PRS-080#022-DP on pharmacodynamic parameters after single administration at 2 mg/kg, 4 mg/kg, and 8 mg/kg BW: serum iron (Fe), ferritin, transferrin saturation, Hb, reticulocyte hemoglobin (RetHb), and reticulocyte count.
- to evaluate the effect of PRS-080#022-DP on hepcidin plasma levels after single administration at 2 mg/kg, 4 mg/kg, and 8 mg/kg BW.
- to collect data on immunogenicity after single PRS-080#022-DP administration at 2 mg/kg, 4 mg/kg, and 8 mg/kg BW.

8 Study design and design rationale

8.1 Overall study design

This is a multi-center, randomized, double-blind, placebo-controlled, single ascending dose phase Ib study in anemic stage 5 CKD patients requiring hemodialysis.

The study will consist of 3 dose cohorts of 2 mg/kg, 4 mg/kg, and 8 mg/kg BW with 8 subjects in each cohort. Using a standard 6+2 design, 6 subjects in each cohort will be randomized to PRS-080#022-DP and 2 subjects in each cohort will be randomized to placebo.

The study is designed to provide maximum safety to the subjects using safety precautions within a cohort and between cohorts as described below.

8.2 Safety precautions within a cohort

In each dose cohort initially 2 subjects will be treated (1 subject will receive PRS-080#022-DP and the other subject will receive placebo). Once the 2 subjects have completed the assessments scheduled at 19 hours after the start of the infusion and all safety and pharmacodynamic data from this period are available, the investigator, Medical Monitor, independent physician and sponsor will review the data and decide whether or not enrollment in the respective cohort is continued. A further 6 subjects will only be enrolled in the cohort if no safety concerns have been identified based on data from the first 2 subjects.

For each cohort (i.e., from the first PRS-080#022-DP administration in one cohort to the termination visit of the last subject of the same cohort) the following safety reassessment criterion applies:

- 1 or more subjects experience a treatment related SAE.

If the criterion is met:

- Randomization and administration of study medication is suspended until the respective investigator(s), independent physician, the Medical Monitor, and the sponsor will decide together on the continuation of randomization from a safety point of view.
- The follow-up for the subjects already treated continues.

8.3 Safety precautions between cohorts (DEC)

An additional precaution will be applied between the cohorts. A DEC will decide on the next dose escalation after first and after second cohort as described in section 6.2. Between the cohorts will be an interval of at least 7 days.

8.4 Study design rationale

A double-blind design is used to eliminate reporting bias. Placebo is considered an appropriate control in the absence of an active comparator with a mechanism of action comparable to that of PRS-080#022-DP.

The pharmacokinetics as well as the pharmacological activity of PRS-080#022-DP may differ in stage

5 CKD patients requiring hemodialysis compared to healthy subjects. Elimination of PRS-080#022-DP may be affected due to renal impairment in this patient population. In addition, hepcidin levels higher than in healthy subjects may affect hepcidin neutralization and, thus, the kinetics of iron release and elevation of transferrin saturation. It is important that these parameters are studied after single dose administration to allow the definition of a dose range and a schedule for subsequent repeated administration of PRS-080#022-DP with the focus at potential changes of hemoglobin concentrations as a result of improved iron mobilization. Furthermore, single administration allows gaining safety information in patients with underlying disease and preparing for further testing by repeated administration.

Treatment at different doses occurs in staggered dose cohorts and dose escalation will be based on safety data by a Dose Escalation Committee. This ensures treatment with a higher dose only when the previous dose was sufficiently safe. The study is randomized, placebo controlled in order to allow for assessing effects of comorbidities, concomitant medication and study procedures on safety and potentially other parameters. PRS-080#022-DP is a pegylated protein and is administered by infusion to obtain systemic distribution.

8.5 Risk/benefit assessment

Based on the experimental animal studies that were carried out, investigation of the safety and tolerability of PRS-080#022-DP showed no special dangers for humans. In addition, results from the first-in-man study revealed no particular hazard. Single PRS-080#022-DP administration was well tolerated in healthy male volunteers. No SAEs occurred. Headache was the most frequent TEAE reported. 9 of 36 subjects experienced 11 events of headache in the PRS-080#022-DP group of which 3 were considered at least possibly related to PRS-080#022-DP but were of mild intensity only.

Further TEAEs considered drug-related by the investigator were one mild injection site erythema and abdominal discomfort and flushing in one subject, both of mild intensity as well. No specific risks could be identified that may raise concerns to investigate safety and efficacy in the target population for PRS-080#022-DP treatment, i.e. patients with chronic kidney disease requiring hemodialysis and suffering from anemia.
Subjects will be enrolled in small cohorts to ensure that safety issues are detected early and no further subjects are unnecessarily exposed to PRS-080#022-DP if any safety concerns are identified. In addition, subjects will be closely monitored during the first hours after PRS-080#022-DP administration.

The study participants in this study are not expected to benefit from the treatment.

9 Subject selection

9.1 Sample size

24 subjects will be enrolled to 3 cohorts of 8 subjects each. The subjects of one cohort will be randomized to either PRS-080#022-DP or placebo in a ratio of 3:1. Six subjects per cohort will receive PRS-080#022-DP and 2 subjects will receive placebo. The PRS-080#022-DP doses will ascend after each cohort starting with 2 mg/kg (first cohort), increasing to 4 mg/kg (second cohort), and ending with 8 mg/kg BW (third cohort).

9.2 Inclusion criteria

Patients meeting the following criteria will be considered for inclusion into the study:

1. Patients with stage 5 chronic kidney disease (CKD) having been on hemodialysis for at least 90 days
2. Male and post-menopausal (≥ 12 months after the last menstruation) female patients of ≥ 18 years and ≤ 100 kg body weight
3. Patients being on stable (less than 30% change) erythropoiesis-stimulating agent (ESA) dose for 6 weeks prior to study medication administration
4. Hemoglobin (Hb) 9.0 – 12.0 g/dL with no changes greater than 1.5 g/dL over the last 6 weeks prior to study medication administration
5. Ferritin ≥ 300 ng/mL.
6. Transferrin saturation (TSAT) ≤ 40%
7. Hepcidin 5.0 – 75.0 nmol/L
8. Male patients with a female partner of childbearing potential agree to use a medically acceptable method of contraception (e.g. condoms, sexual abstinence, vasectomy), not including the rhythm method for 30 days after administration of the study medication.
9. The patient is legally competent, has been informed of the nature, the scope and the relevance of the study, voluntarily agrees to participation and the study's provisions, and has duly signed the informed consent form (ICF). Subject agrees to comply with the protocol-mandated procedures and visits.

9.3 Exclusion criteria

A patient will not be eligible for inclusion if any of the following criteria applies:

1. Anemia due to causes other than CKD, including hemoglobinopathies, hemolytic anemias, myelodysplasia or malignancy
2. Blood transfusion within 2 months before administration of study medication.
3. Any iron treatment from 1 week before study medication administration until 1 week after study medication administration.
4. Previous enrollment in this study
5. Current or previous (within 60 days before study medication administration) treatment with another investigational drug and/or medical device or participation in another clinical study.
6. Pregnancy or breast-feeding, women of child-bearing age.
7. Employees of the sponsor or subjects who are employees or relatives of the investigator
8. Known allergy to any component of the PRS-080#022-DP formulation
9. Positive for hepatitis B surface antigen (HBs Ag), anti-hepatitis C virus antibody (anti-HCV Ab), or HIV
10. Planned surgery during the study period
11. Unwilling or unable to comply with the protocol, in the judgment of the investigator
12. Unstable angina, myocardial infarction, percutaneous transluminal coronary angioplasty/stents, apoplex or coronary artery bypass grafting <3 months prior screening.
13. Congestive heart failure: New York Heart Association Class III or IV.
14. Any medical condition that in the judgment of the investigator might interfere with study participation or jeopardize subject’s safety during the study (e.g. active infection).

10 Randomization, blinding and unblinding procedures

10.1 Blinding

During the entire study, study participants, investigators, the sponsor and all other persons involved in the conduct of the study will be blinded to treatment, except for the site for reconstitution and distribution preparing the patient-individual infusion bags, and the monitor responsible for drug accountability at the site for reconstitution and distribution.

To maintain the blind, the infusion bags with IMP and placebo after reconstitution will have the

same appearance and will be identified by the subject number and the information "PRS-080#022-DP or placebo". In addition, solutions of both PRS-080#022-DP and placebo are colorless and clear. Thus, neither the subject nor the investigators will be aware of whether the drug administered is PRS-080#022-DP or placebo. To minimize the potential for bias, treatment randomization information will be kept confidential by the responsible unblinded personnel and will not be released to the investigator, other study site personnel, the sponsor or its designee and clinical research associate (CRA) monitoring patient data until after database hard lock.

The premature breaking of the code should be restricted to emergency cases in which knowledge of the administered drug is necessary for adequate treatment. Whenever possible, the sponsor should be contacted before breaking the blinded emergency code. Should any code be broken, the respective subject will be withdrawn from further participation in the study and a written explanation must be given by the investigator. The sponsor must be notified immediately.

10.2 Subject identification

After informed consent has been signed, the subjects will be allocated screening numbers for the study screening procedures until randomization. These will consist of the site number, an S for screening, and the number of the subject according to the order of appearance at a site, starting with the number 01, 02, 03 etc. (e.g. 1-S-01 for the first subject screened at site number 1).

If eligible, subjects will be randomized into the study by allocating unique subject numbers. These numbers will consist of 3 digits, the first digit representing the site number and the last 2 digits represent the number of the subject according to the order of appearance at a site, starting with the number 01, 02, 03 etc (e.g., 1-01 for the first subject randomized at site number 1). The subject number will be assigned by means of IWRS.

In case of replacement of subjects, the next available number will be allocated.

10.3 Randomization

Eligible subjects who have signed the informed consent will be randomized into the study. The randomization must be documented in the patient file and the case report form.

Subjects will be randomly assigned to treatment groups by means of a computer-generated randomization list with a 3:1 allocation for the 8 subjects of each cohort (6 subjects PRS-080#022-DP – 2 subjects placebo). There will be 1:1 randomization for the first two subjects of each cohort and a 5:1 randomization for the remaining subjects of each cohort.

Eligible subjects will be randomized by means of IWRS.

The IWRS provider will supply the site for reconstitution, preparation of infusion bags and distribution as well as the investigators with user guides for the automated system.

The investigator will have the option to selectively break the code for an individual subject via the eCRF.

Assignment of subjects to treatment dose (i.e. assignment to the cohort) will be dictated by the dose cohort open at the time of the randomization.

11 Treatments

11.1 Study medication

All medications supplied by the sponsor will be manufactured, tested, and released according to current GMP guidelines.

11.1.1 PRS-080#022-DP

Name:	PRS-080#022-DP
Formulation:	5 mL clear solution
Active ingredient:	PRS-080#022
Inactive ingredients:	PBS pH 6.5 (20 mM NaH_2PO_4; 115 mM NaCl; pH6.5)
Protein strength:	8.1 mg/mL
Administration:	Intravenous infusion for 60 min
Doses:	Single dose of 2 mg/kg, 4 mg/kg or 8 mg/kg body weight
Container:	10 mL glass vials with 20 mm stoppers for injection vials and flip-off caps
Storage:	-20°C ± 5°C
Infusion solution:	Required amount of PRS-080#022-DP will be reconstituted in 0.9 % NaCl to a final volume of 100 mL

11.1.2 Placebo

Name:	PRS-080#022-DP-Placebo#001
Formulation:	5 mL clear solution
Active ingredient:	None
Inactive ingredient:	PBS pH 6.5 (20 mM NaH_2PO_4; 115 mM NaCl; pH6.5)
Protein strength:	None
Administration:	Intravenous infusion for 60 min
Doses:	NA
Container:	10 mL glass vials with 20 mm stoppers for injection vials and flip-off caps
Storage:	-20°C ± 5°C
Infusion solution:	Required amount placebo will be reconstituted in 0.9 % NaCl to a final volume of 100 mL

11.1.3 Description, packaging and labeling

Study medication will be packed and labeled according to the applicable regulatory requirements.

It will be provided in cardboard outer packages containing 10 mL glass vials with 5 mL clear solution each.

Vial labels will include at least the following:
Sponsor name and address, CRO name and address, name of study medication: PRS-080#022-DP or Placebo, strength of study medication, batch number, for intravenous infusion after reconstitution, study number, space where subject number can be added manually by the investigator or pharmacist, and medication number.

Outer package labels will include at least in addition:
EudraCT number, expiry date, phone numbers of sponsor and CRO, no. of vials with 5 mL solution, application and dosing according to the clinical protocol, for clinical trials only, store at -20 ± 5°C

PRS-080#022-DP will be reconstituted in infusion bags by the site for reconstitution, preparation of infusion bags and distribution. Infusion bags provided to the investigators will be labeled by the pharmacist with a tear-off part/label. When study medication is administered, the investigator will remove the tear-off part of the label and attach it to the Drug Administration Form for verification by the monitor. The site will keep the used infusion bag until monitoring was performed.

11.1.4 Storage and stability

The manufacturer, the site for reconstitution and distribution and the investigators are responsible for the safe and proper handling and storage of the study medication at their site. The study medication vials have to be stored at – 20 ± 5°C at the manufacturer. It must be stored in a locked facility with access limited to authorized personnel. The site for reconstitution and distribution will prepare and ship the infusion bags following reconstitution to the sites according to the Instruction Manual For The Investigation Medicinal Product. The appropriate amount of study medication will be reconstituted in 0.9 % NaCl to a final volume of 100 mL infusion solution. The infusion bags will be shipped temperature controlled in an insulted box. After receipt, the infusion bags are stored at 2 – 8°C.

11.1.5 Treatment dose and administration

The study medication will be administered by slow infusion over 60 min using an infusion pump with an infusion flow rate of about 1,67 mL/min. In case necessary, infusion may be paused or slowed down up to a complete infusion period of up to two hours.

One of three different ascending doses, 2 mg/kg, 4 mg/kg, or 8 mg/kg BW, will be administered once per subject. The day of study medication administration is defined as **Day 0**. One to 2 hours before the planned infusion the respective infusion bag should be removed from the refrigerator to allow the infusion solution to adapt to room temperature (the adaption to room temperature should not exceed 4 hours). Infusion has to be started before the expiry date and time indicated on the infusion bag label. A manual for handling and administration of the study medication (Site Study Medication Manual) will explain the procedures in detail. The investigator must ensure that the study medication is administered only to subjects randomized in this study.

No study medication will be handed out to the subjects. Study medication will only be administered at the study site.

11.1.6 Infusion stopping criteria

Infusion will be stopped:

- If an allergic reaction/hypersensitivity occurs that needs immediate medical intervention.
- If any severe AE or other event or condition occurs that in the opinion of the investigator requires interruption of the infusion.

The investigator will evaluate if the infusion can be continued. If the infusion needs to be stopped permanently the subject will be withdrawn from the study and the termination page of the eCRF has to be completed accordingly. If possible, the safety assessments according to Day 28 should be done for safety reasons (see section 12).

11.1.7 Drug accountability and subject compliance

Study medication must not be used outside the context of this study protocol. The investigator or authorized staff must document the receipt, dispensation, and return of all study medication received during this study.

Records on receipt, use, return, loss, or other disposition of study medication must be maintained. The investigator or, if applicable, pharmacist must confirm receipt. Records on study medication delivery to the site, the inventory at the site, the use by each subject, and the return to the sponsor or sponsor's designee must be maintained by the investigator and/or a pharmacist or another appropriately trained individual at the investigational site. These records will include dates, quantities, batch numbers, and the unique code numbers assigned to the study medication and subjects. The investigators and/or pharmacist must maintain records documenting that the subjects were provided with the doses specified in the protocol. The study medication will be administered at the study site only. No medication will be given out to the patient.

11.2 Previous and concomitant medication

All previous medication administered within one month prior to study drug administration must be documented in the corresponding section of the eCRF. Other relevant previous medication as judged by the investigator should also be documented in the eCRF.

All treatments being taken by the subjects at entry into the study and all treatments given in addition to the study medication during the study are regarded as concomitant treatments and must be documented in the eCRF.

Any iron treatment is not allowed from 7 days before study medication administration until 7 days after study medication administration. All other required treatments are allowed during the study period.

In case of an emergency all treatments, including immediate treatment of allergic or anaphylactic reactions (steroids, H1, H2 antihistaminergic agents, intravenous fluids, oxygen, epinephrine and equipment for cardiopulmonary resuscitation) are allowed.

12 Study schedule

12.1 Study conduct

An overview on study conduct is provided in the schedule of procedures (see synopsis).

It is recommended that enrollment of patients (study medication administration) will be done on a Wednesday or Thursday when a regular dialysis is scheduled starting the dialysis in the morning. It

is expected that this would fit the patient schedule best. On Day 7 and thereafter patient visits can be shifted to the afternoon if desired.

12.2 Screening/Baseline

The subject will be fully informed of all study procedures and implications both verbally and in writing via use of a patient information sheet. See Section 19.3 for more information regarding the subject consent process.

Screening Visit: ≤ 16 days before start of infusion of study medication

Subjects, for whom written informed consent has been obtained, will undergo the following assessments:

- Inclusion and exclusion criteria check
- Demographic data, height and weight for dose calculation
- Medical history
- Previous and concomitant medication
- Vital signs and body temperature
- Physical examination including ECG
- Blood draws:
    - Safety lab: hematology incl. Hb, Ret, RetHb, biochemistry, coagulation
    - Serology
    - Pregnancy test
    - Hepcidin
    - PD assessment: Fe, TSAT, ferritin
    - ADA

Hb concentration can be reassessed once within the 16-day screening period in case the investigator determines so. In this case, a patient is eligible for enrolment when the result of the second Hb determination complies with the inclusion criteria. As soon as eligibility is confirmed, the investigator will randomize the patient by IWRS and will thereby order study medication.

12.3 Treatment – Day 0

The following study evaluations will be done after dialysis and **before** study drug infusion:

- Re-check inclusion and exclusion criteria

- PTEE questioning/observation
- Concomitant medication
- Vital signs and body temperature
- Blood draws:
  - Hepcidin
  - PD assessment: Fe, TSAT, ferritin
  - PK

Infusion

Start of infusion is defined as **Day 0 and Time 0**. Subjects will receive PRS-080#22-DP by slow infusion over 60 minutes.

Subjects will be monitored for TEAEs and use of concomitant medications from start of the infusion until 5 hours after start of the infusion. Vital signs and body temperature will be measured at 30 min, 3 and 5 hours after start of the infusion.

At 30 min after start of the infusion the following assessments will be done:

- Vital signs and body temperature

At the **end** of infusion (generally **1 h** after start of infusion), the following assessments will be done:

- Blood draws:
  - Hepcidin
  - PD assessment: Fe, TSAT, ferritin
  - PK

3 h after start of infusion the following assessment will be done:

- Vital signs and body temperature

5 h after start of infusion the following assessments will be done:

- Blood draws:
  - Hepcidin
  - PD assessment: Fe, TSAT, ferritin
  - PK
- Vital signs and body temperature

Subjects will be instructed to call the study site after discharge if they experience any severe reactions at the injection site, signs of anaphylactic reactions, or other severe AEs.

12.4 Follow-up

Day 1

The following study evaluations will be done **19 h** after start of infusion:

- TEAE questioning/observation
- Concomitant medication
- Vital signs and body temperature
- Physical examination
- Blood draws:
    - Safety lab: hematology incl. Hb, Ret, RetHb, biochemistry
    - Hepcidin
    - PD assessment: Fe, TSAT, ferritin
    - PK

The following study evaluations will be done **29 h** after start of infusion:

- TEAE questioning/observation
- Concomitant medication
- Blood draws:
    - Hepcidin
    - PD assessment: Fe, TSAT, ferritin
    - PK

Day 2

The following study evaluations will be done **44 h – 48 h** after start of infusion (**before dialysis** on Day 2):

- Vital signs and body temperature
- Blood draws:
    - Safety lab: hematology incl. Hb, Ret, RetHb, biochemistry
    - Hepcidin

- PD assessment: Fe, TSAT, ferritin
- PK

The following study evaluations can be done before, during or after dialysis:

- TEAE questioning/observation
- Concomitant medication

Physical examinationThe following study evaluations will be done **48 h – 52 h** after start of infusion (**after dialysis** on day 2):

- TEAE questioning/observation
- Concomitant medication
- Blood draws:
  - Hepcidin
  - PK

Day 3

The following study evaluations will be done **70 h** after start of infusion:

- TEAE questioning/observation
- Concomitant medication
- Vital signs and body temperature
- Blood draws:
  - Hepcidin
  - PD assessment: Fe, TSAT, ferritin
  - PK

Day 5

The following study evaluations will be done **116 h – 120 h** after start of infusion **before dialysis** on Day 5:

- Vital signs and body temperature
- Blood draws:
  - Hepcidin
  - PD assessment: Fe, TSAT, ferritin

  - PK

The following study evaluations can be done before, during or after dialysis:

- TEAE questioning/observation
- Concomitant medication

Day 7

The following study evaluations will be done before dialysis:

- Vital signs and body temperature
- Blood draws:
  - Safety lab: hematology incl. Hb, Ret, RetHb, biochemistry
  - Hepcidin
  - PD assessment: Fe, TSAT, ferritin
  - PK

The following study evaluations can be done before, during or after dialysis:

- TEAE questioning/observation
- Concomitant medication
- Physical examination

Day 14

The following study evaluations will be done before dialysis:

- Vital signs and body temperature
- Blood draws:
  - Hepcidin
  - PD assessment: Fe, TSAT, ferritin
  - PK

The following study evaluations can be done before, during or after dialysis:

- TEAE questioning/observation
- Concomitant medication
- Physical examination

Day 21

The following study evaluations will be done before dialysis:

- Vital signs and body temperature
- Blood draws:
    - Hepcidin
    - PD assessment: Fe, TSAT, ferritin
    - PK

The following study evaluations can be done before, during or after dialysis:

- TEAE questioning/observation
- Concomitant medication
- Physical examination

12.5 Termination Visit – Day 28

The following study evaluations will be done before dialysis:

- Vital signs and body temperature
- Blood draws:
    - Safety lab: hematology incl. Hb, Ret, RetHb, biochemistry, coagulation
    - Hepcidin
    - PD assessment: Fe, TSAT, ferritin
    - ADA
    - PK

The following study evaluations can be done before, during or after dialysis:

- TEAE questioning/observation
- Concomitant medication
- Physical examination including ECG

12.6 Early termination

In case a subject prematurely discontinues the clinical investigation (for reasons see Section 18), the subject will be asked to complete an early termination visit at which all assessments normally performed at the termination visit Day 28 will be completed.

In case of early termination, reasons, circumstances and findings should be fully described on the "Early Termination" page in the eCRF respecting the subject’s rights.

In case a patient decides to terminate the study after screening but before drug administration, this patient will be treated as screening failure and will be substituted.

12.7 Time-windows

The time-windows allowed for the scheduled visits will be as follows:

Screening to Day 0	≤ 16 days
Day 0 : 1 h	± 10 min
Day 0 : 5 h	± 10 min
Day 1 : 19 h	± 2 h
Day 1 : 29 h	± 2 h
Day 2 : 44 h – 48 h	± 2 h
	Within 30 min before dialysis
Day 2 : 48 h – 52 h	± 2 h
	Within 30 min after dialysis
Day 3 : 70 h	± 2 h
Day 5 : 116 h – 120 h	± 2 h
	Within 30 min before dialysis
Day 7	Within 1 h before dialysis
Day 14	Within 1 h before dialysis
Day 21	Within 1 h before dialysis
Day 28 ± 2 days	Within 1 h before dialysis

On Day 2 – 44 h, Day 5 – 116 h, Day 7, Day 14, Day 21 and Day 28 TEAE questioning/observation concomitant medication, physical examination and ECG, if applicable, can be performed during or after dialysis. For Days 7 to 28 dialysis can be performed according to the individual patient dialysis schedule. Date and time of dialysis will be documented in the eCRF.

13 Efficacy assessments

As this study is designed to evaluate the safety and tolerability of PRS-080#022-DP in CKD patients under hemodialysis, efficacy will not be assessed.

The activity of PRS-080#022-DP will be measured by pharmacodynamic parameters as indicated in section 15.

14 Pharmacokinetic assessments

14.1 Pharmacokinetic assessment

Plasma concentrations of PRS-080#022-DP will be determined at different time points after study drug administration and pharmacokinetic parameters will be determined. PRS-080#022-DP will be determined as total PRS-080#022-DP and as free PRS-080#022-DP, whereas total PRS-080#022-DP is not bound or bound to its target hepcidin and free PRS-080#022-DP is not bound to hepcidin.

14.2 Specimen collection and shipment

Blood samples for pharmacokinetic analysis will be obtained at the time points indicated in the table of procedures in the synopsis. The blood should not be sampled from the infusion site to ensure data integrity.

Blood will be obtained and plasma will be prepared from each sample according to the separate sample analysis plan.

At all visits, date and time of blood collection and study medication administration must be recorded in the eCRF.

Venous blood samples will be collected in appropriately labeled heparinised monovettes. Details on blood collection and processing will be outlined in a separate manual. Each sample will be labeled with the following information: study number, subject number, visit number, sample number, and aliquot number. All labels, for all storage conditions, must adhere to the tubes and be legible. The investigator will be responsible for the proper storage of biological samples at the site at approximately -70°C or below until sample shipment.

Biological samples should be packed and shipped according to the respective manual to

synlab Labor München Zentrum

Bayerstraße 53

80335 München

14.3 Bioanalytical methods

Validated ligand binding assays will be employed for the quantification of total and free PRS-080#022-DP in plasma. Whereas total PRS-080#022-DP is determined in a sandwich assay employing two different antibodies for capturing and detection, free PRS-080#022-DP is captured by hepcidin and detected with an antibody. Separate analytical study plans will be provided before the analytical part of the study and the results will be described in an analytical report.

14.4 Pharmacokinetic parameters

The following pharmacokinetic parameters will be calculated for each subject from measured total and free PRS-080#22-DP plasma concentrations:

C_{max}	=	measured maximal concentration
t_{max}	=	time of observed maximum concentration
λ_z	=	terminal rate constant
AUC_{0-t}	=	area under the concentration time curve (time 0 to last sample with a quantifiable concentration)
$AUC_{0-\infty}$	=	area under the concentration time curve from time 0 extrapolated to infinity
MRT	=	mean residence time
$t_{½}$	=	terminal half-life, from λ_z
V_{SS}	=	Volume of distribution at steady state

Pharmacokinetic parameters will be calculated by non-compartmental or model-free methods, e.g. linear trapezoidal rule for AUC, log-linear regression for λz etc (5).

The value of $AUC_{0-\infty}$ will be considered unreliable if the terminal area beyond the last quantified sample is greater than 20% of the total $AUC_{0-\infty}$, but will be reported nonetheless.

15 Pharmacodynamic assessments

To assess the activity of PRS-080#022-DP, the following pharmacodynamic assessment will be done:

- Hepcidin plasma concentration
- Markers with fast response kinetics: iron, transferrin saturation and ferritin in blood serum

- Markers with slower response kinetics: hemoglobin, reticulocytes and reticulocyte hemoglobin in whole blood

Blood draws, sample handling, processing and shipment are described separately in a laboratory manual provided by the central lab. Each sample will be labeled to indicate at least: the study number, subject number, cohort number, and visit day and time point. Details on time points for blood draws are given in the schedule of assessments in the synopsis.

The concentrations of hepcidin and the pharmacodynamic biomarkers will be determined by different methods. Separate analytical study plans and clinical laboratory protocols will be provided before the start of the analytical parts of the study, including details on method performance for the parameters assessed. Characteristics of the analytical methods will be described in the final study report.

16 Safety assessments

16.1 Adverse events

16.1.1 Definitions

An **AE** is any untoward medical occurrence in a subject administered study medication and which does not necessarily have to have a causal relationship with the treatment. An AE can therefore be any unfavorable and unintended sign (including an abnormal clinically significant laboratory finding, for example), symptom, or disease temporally associated with the use of the study medication, whether or not considered related to study medication.

Any worsening in severity or frequency of a concomitant disease or any new disease diagnosed in the study must be documented as an AE.

A surgery or procedure scheduled to occur during the study will not be considered an AE if the surgery or procedure will be performed for a pre-existing condition and the surgery or procedure was pre-planned prior to study entry. However, if the pre-existing condition deteriorates unexpectedly during the study (e.g. surgery performed earlier than planned), then the deterioration of the condition for which the surgery or procedure is being done will be considered an AE.

AEs that occur between signing the ICF and the time when the subject is administered study medication (Day 0, Hour 0:00) are defined as "**pre-treatment-emergent**" events **(PTEE).**

All AEs which occur after administration of study medication are defined as **treatment-emergent adverse events (TEAE)**.

A **serious adverse event (SAE)** is any AE occurring at any dose that:

- results in death
- is life-threatening
- requires inpatient hospitalization or prolongation of existing hospitalization
- results in persistent or significant disability/incapacity
- is a congenital anomaly/birth defect
- is an important medical event/other

Important medical events that may not have resulted in death, were not life-threatening, or did not require hospitalization are considered as an SAE when, based upon appropriate medical judgment, they may have jeopardized the subject and required medical or surgical intervention to prevent one of the outcomes listed in this definition.

Adverse drug reactions (ADR) are all noxious and unintended responses to study medication related to any dose. The phrase' responses to study medication' means that a causal relationship between study medication and an AE is at least a reasonable possibility, i.e. the relationship cannot be ruled out.

All AEs judged by either the reporting investigator or the sponsor as having a reasonable causal relationship to the study medication qualify as ADRs.

An **unexpected ADR** is an adverse reaction, the nature and severity of which is not consistent with the applicable reference document (Investigator's Brochure, SmPC, product information). Reports which add significant information on specificity or severity of a known, already documented AE constitute unexpected AEs, too. For example, an event more specific or more severe than described in the reference document would be considered 'unexpected'. Specific examples would be: acute renal failure as a labeled AE with a subsequent new report of interstitial nephritis or hepatitis with a first report of a fulminant hepatitis.

A **suspected unexpected serious adverse reaction (SUSAR)** is a suspected adverse reaction related to the study medication that is both unexpected and serious. An unexpected adverse reaction is one that is not reported in the reference document (Investigator's Brochure, SmPC, product information). If an SAE is not previously documented in the reference document in its current version and it is judged to be related to the study medication as assessed by either the Medical Monitor or the clinical investigator, the event qualifies as SUSAR and as such will be subject to expedited regulatory reporting.

16.1.2 Classification of adverse events

Causality

The investigator will fully assess each event in their best judgment and assign one of the following causalities:

- Definitely related: There is a reasonable temporal relationship between the study intervention (e.g. study medication and/or procedure) and the AE, and no other obvious explanation for the AE exists.
- Possibly related: There is a reasonable temporal relationship between the study intervention (e.g. study medication and/or procedure) and the AE, and the AE could also be explained by disease, other medications, or another obvious explanation for the AE exists.
- Not related: The AE is independent of any study intervention (e.g. study medication and/or procedure), or evidence exists that the event is definitely related to another etiology.

Definitely and possibly related AEs are considered as related with regard to SUSAR reporting.

Severity

The severity of AEs will be graded according to the National (US) Cancer Institute-Common Toxicity Criteria for Adverse Events (NCI-CTCAE), Version 4.03 as follows:

Grade 1: mild; asymptomatic or mild symptoms; clinical or diagnostic observations only; intervention not indicated

Grade 2: moderate; minimal, local or noninvasive intervention indicated; limiting age-appropriate instrumental activities of daily living (ADL)*

Grade 3: severe or medically significant but not immediately life-threatening; hospitalization or prolongation of hospitalization indicated; disabling; limiting self care ADL**

Grade 4: Life-threatening consequences; urgent intervention indicated

Grade 5: Death related to AE

* Instrumental ADL refer to preparing meals, shopping for groceries or clothes, using the telephone, managing money, etc.

** Self care ADL refer to bathing, dressing and undressing, feeding self, using the toilet, taking medications, and not bedridden.

Outcome Categories

- Recovered: The subject has fully recovered from the event or the condition has returned to the level observed at baseline
- Recovering: The event is improving but the subject is still not fully recovered
- Not recovered: The event is ongoing at the time of reporting and the subject has still not recovered
- Recovered with sequelae: As a result of the AE, the subject suffered persistent and significant disability/incapacity (e.g. became blind, deaf or paralyzed)
- Fatal: The subject died due to the event. If the subject died due to other circumstances than the event, the outcome should be stated otherwise (e.g. not recovered or recovering)
- Unknown: If outcome is not known or not reported

16.1.3 Documentation of adverse events

All AEs that occur after the subject has signed the Informed Consent Form until the subject's study completion must be recorded (PTEE and TEAE). The occurrence of AEs should be sought by non-directive questioning of the subject at each visit during the study i.e. "Have you experienced any problems since your last contact?" AEs also may be detected when they are volunteered by the subject during or between visits or through physical examination, laboratory test, or other assessments.

All AEs which occur during the study will be recorded in the subject's AE section of the eCRF and will include the following information: AE (diagnosis or syndrome(s) if known, signs or symptoms if not known, a description, date of onset and resolution, severity, intensity, relationship to study medication, relationship to study procedure, action taken and outcome. For SAEs, the SAE Form must also be completed (see Section 16.1.4).

16.1.4 Documentations of serious adverse events

All SAEs will be collected and recorded on SAE Report Forms. The investigator must assess the relationship to the study medication, complete the SAE Report Form in English, and send the completed report IMMEDIATELY, within 24 hours, to the Medical Monitor. An SAE report is considered valid if the following minimum criteria are provided on the transmitted form:

- Identifiable subject
- Identifiable reporting source
- Medical event that fulfils a seriousness criterion

The investigator will be requested to supply as much detailed information as possible regarding the SAE that is available at the time of the initial contact. The investigator should also complete missing or requested information and submit follow-up reports to the Medical Monitor until the SAE has resolved or, in the case of permanent impairment, until the SAE has stabilized.

The Medical Monitor will inform the sponsor about all SUSARs within 24 hours after receipt of a valid SAE report. SAE reports which are not subject to expedited reporting will be sent to the sponsor by the Medical Monitor within 3 calendar days after receipt of the respective valid report.

SUSARs will be handled and reported to the competent authority (CA), relevant ethics committees (ECs), and all investigators involved in accordance with the “Detailed guidance on the collection, verification and presentation of adverse event/reaction reports arising from clinical trials on medicinal products for human use” (‘CT-3’) based on Directive 2001/20/EC and in accordance with the German GCP regulation (“GCP-Verordnung”).

16.1.5 Follow-up of adverse events

All SAEs judged to be related to the study medication must be followed by the investigator until the subject has recovered, recovered with sequelae, died, or until the investigator determines that the subject’s condition is stable, whichever occurs first. All other AEs must be followed by the investigator until the subject has recovered or until the end of the safety follow-up period (30 days after last visit) whichever comes first, and until all AE-related queries for the subject have been resolved. The investigator will take all appropriate and necessary therapeutic measures required for resolution of the AE, if applicable. All efforts to collect follow-up information must be documented in the source data.

Follow-up information should be supplied on the AE pages in the eCRF and/or the SAE Form. The investigator must forward follow-up information on SAEs to the sponsor within 24 hours of the investigator's awareness time.

16.2 Laboratory investigations

Laboratory assessments will include determination of the following parameters:

16.2.1 Hematology

Hematocrit (HCT), hemoglobin (Hb), red blood cell count (RBC), mean corpuscular volume (MCV), mean corpuscular hemoglobin (MCH), mean corpuscular hemoglobin concentration (MCHC), white blood cell count (WBC, leukocytes), platelet blood count (PBC, thrombocytes); reticulocytes, reticulocyte hemoglobin concentration.

16.2.2 Biochemistry

Sodium (Na), potassium (K), calcium (CA), phosphate (P), iron, Aspartate-amino-transferase (AST/GOT), alanine-amino-transferase (ALT/GPT), alkaline phosphatase (AP), gamma-glutamyl-transferase (G-GT), creatine-phosphokinase (CK), Glucose (GLUC), cholesterol (CHOL), blood urea (UREA), creatinine (CREA), total bilirubin (T-BILI), albumin (ALB), high sensitivity c reactive protein (hs CRP).

16.2.3 Coagulation

Prothrombin time (QUICK INR), activated partial thromboplastin-time test (aPTT).

16.3 Vital signs, routine physical examinations and ECG

16.3.1 Documentation

Vital signs, physical examination, and ECG values will all be assessed by the investigator as:

0 = normal
1 = abnormal, but without clinical relevance
2 = abnormal with clinical relevance

If any clinically relevant finding of these assessments will be observed at screening, these must be documented in the medical history section of the eCRF; any new clinically relevant finding compared to screening must be documented as AE in the eCRF.

16.3.2 Vital signs

Systolic and diastolic pressure as well as heart rate should be measured either in supine or sitting position after 5 minutes of rest. The right or left arm may be used. However, the position and the arm used for measurement should be kept constant throughout the study for an individual subject. Furthermore, the body temperature will be measured.

16.3.3 Physical examination

A full physical examination will be performed including:

- General appearance
- Head
- Ears
- Eyes
- Nose/mouth/throat
- Skin
- Thyroid
- Lungs/thorax
- Heart/cardiovascular system
- Abdomen
- Musculoskeletal
- Extremities

- Lymph nodes
- Neurological
- Other

16.3.4 12-lead electrocardiogram

A standard 12-lead ECG will be performed. The investigator will assess the ECG based on heart-rate, P-wave, PQ, QRS, QT, occurrence of re- or depolarization disorders, arrhythmic disorders, or other abnormalities.

16.4 Body temperature

Body temperature will be determined using an ear thermometer.

16.5 Immunogenicity

Assessment of immunogenicity will be based on measurement of ADA.

17 Biostatistical methods

The planned analyses are of exploratory nature without any formal statistical hypotheses. All parameters will be descriptively analyzed using standard statistical methods.

Tables and graphs, as well as subject listings will be presented by dose groups.

Further details on statistical analysis will be described in the SAP.

17.1 Sample size calculation

No formal statistical hypotheses are being tested for this standard design dose escalating study. Refer to section 9.1 for the planned number of subjects. The sample size is considered being sufficient to evaluate the safety and tolerability in this study.

17.2 Analysis sets and types of analyses

The statistical analysis will be based on separate analysis populations, defined as follows:

- Safety Set:
  All subjects who received study medication.
- Pharmacokinetic Set:
  All subjects who are included in the Safety Set and who satisfactorily completed a pharmacokinetic blood sampling period without any major protocol violations which would render the data unreliable.

- Pharmacodynamic Set:
  All subjects who are included in the Safety Set and who satisfactorily completed a pharmacodynamic blood sampling period without any major protocol violations which would render the data unreliable.

A period of pharmacokinetic/pharmacodynamic sampling will be considered to have been completed satisfactorily if no more than 6 blood samples have been missed by negligence of the subject or the investigator, or have no available results, and provided that the pattern of missing points still allows calculation of pharmacokinetic/ pharmacodynamic parameters.

17.3 Analysis of study conduct and subject disposition

The disposition of subjects and analysis sets will be summarized with descriptive statistics.

Important protocol deviations, such as deviations from inclusion and exclusion criteria, relevant deviations in sampling times or from the planned time schedule of safety assessments will be reported.

17.4 Patient demographics/other baseline characteristics

Demographic variables and baseline characteristics will be summarized with descriptive statistics.

17.5 Extent of exposure

All subjects will receive one infusion of PRS-080#022-DP at the pre-specified fixed doses of 2 mg/kg, 4 mg/kg, and 8 mg/kg, based on BW, or placebo.

17.6 Analysis of the primary endpoints / Safety evaluation

17.6.1 Statistical and analytical issues

Safety data will be evaluated descriptively only. There will be no adjustment for covariates or centers. The safety set will be used for analysis. All data will be used to their largest possible extent without any attempt to impute or extrapolate missing data.

17.6.2 Adverse events

AEs will be coded according to the Medical Dictionary for Regulatory Activities (MedDRA). The most recent MedDRA version at the start of the study will be used. TEAEs will be summarized and tabulated according to the primary system organ class and preferred term. Separate analyses will be conducted using severity, seriousness, and relationship to the investigational product.

17.6.3 Clinical laboratory and other safety measures

Clinical laboratory parameters (including high-sensitive C-reactive protein) will be listed in full with suitable flags for abnormal values (clinically irrelevant / relevant) and will be summarized with descriptive statistics by time point. A separate listing will show all abnormal results considered clinically relevant.

17.6.4 Vital signs, body temperature, physical findings, and other observations related to safety

Vital signs (systolic blood pressure, diastolic blood pressure and heart rate) will be summarized descriptively by time point.

Other clinical data derived in the course of the study documented in the eCRF will be listed and summarized as appropriate. Any unusual finding will be commented upon in the report.

17.7 Analysis of secondary endpoints

17.7.1 Pharmacokinetic evaluation

PK concentrations as well as PK parameters will be analyzed with descriptive statistics.

For each subject, concentration-time curves will be plotted on a linear and log-linear scale simultaneously.

17.7.1.1 Statistical and analytical issues

In order to achieve a better approximation to a normal distribution, pharmacokinetic parameters related to concentrations (AUC, C_{max}) will be logarithmically transformed before analysis.

Further parameters of interest that might be evaluated statistically will be logarithmically transformed in case they are more likely to be log-normally than normally distributed (e.g. t½).

All data will be used to their maximum possible extent, but without any imputations for missing data. If a subject has appreciable or critical data points missing, he/she should be excluded from analysis. However, if a subject is missing an inconsequential data point, e.g. final collection time sample lost due to tube breakage and preceding sample unquantifiably low, then the subject’s remaining data can still be used.

17.7.2 Pharmacodynamic evaluation

Pharmacodynamic parameters (hepcidin, serum iron, ferritin, transferring saturation, hemoglobin, reticulocyte hemoglobin, and reticulocyte count) will be summarized descriptively by time point, including changes from baseline.

17.7.3 Immunogenicity

ADAs will be analyzed descriptively by time point.

17.8 Interim analysis

No formal interim analyses are planned.

18 Subject withdrawal from study participation

18.1 Subject withdrawal

Subjects may withdraw from the study at their own request, and may be withdrawn upon request of the investigator, or by the sponsor at any time and for any reason. Reasons for removing a subject from the study may include:

- the subject does not adhere to study rules and procedures
- the subject wishes to withdraw from the study (every subject has the right to withdraw from the study at any time for any reason without prejudice to his future medical care by the investigator)
- the subject develops an AE necessitating withdrawal, or requires an unacceptable concomitant medication
- subject violates against inclusion and exclusion criteria
- the investigator feels it is in the subject's best interest to terminate participation
- the study is terminated by the sponsor
- infusion has to be stopped due to unacceptable AEs (see section 11.1.6).

Subjects that are prematurely withdrawn should undergo the safety assessments according to Day 28 if possible (Early Termination Visit) as specified in the table of procedures (see synopsis). The corresponding results/findings and the reason for withdrawal are documented on the early termination page of the eCRF.

If a subject fails to return for study visits (lost to follow-up), reasonable efforts should be made to contact the subject and to complete the withdrawal assessments as stated above.

The reason "consent withdrawn" should be documented on the early termination page in the eCRF.

For follow-up of AEs after early termination, please refer to section 16.1.5.

After withdrawal, subjects will be treated according to the individual needs at the discretion of the investigator.

18.2 Subject replacements

All screening failures are replaced. A screening failure is any subject that signed the informed consent form but is not enrolled in the study. In this study enrollment is defined as start of study drug administration (infusion). A patient who failed screening can be screened again after a time period defined by the investigator and after re-signing the ICF.

Subjects that terminate the study after receiving any study medication are not replaced.

For discontinuation criteria of the entire study please refer to section 20.1.

19 Ethical and legal requirements

19.1 Good clinical practice, general requirements, and study registration

The study will be conducted in accordance with the International Conference on Harmonization (ICH) Guideline for Good Clinical Practice (GCP, CPMP/ICH/135/95), the appropriate national regulations and the Declaration of Helsinki.

The study will also be carried out in keeping with applicable local law(s) and regulation(s). This may include audits by the sponsor and the sponsor designee, and/or inspections by regulatory authority representatives at any time.

The investigator must agree to the inspection and/or audit of study-related records by the Regulatory Authority and/or sponsor and sponsor designee, and must allow direct access to source documents to the Regulatory Authority and/or sponsor and sponsor designees.

This clinical study will be registered in a publicly accessible database before start of recruitment.

19.2 Independent ethics committee or institutional review board

Before the initiation of the clinical study, the final protocol, any amendments if applicable, the subject information sheet and consent form, as well as any additional documents which are required by German regulations and the IEC will be submitted to the competent IEC for review. A favorable opinion for the clinical study must be obtained from the IEC before any subject is enrolled at a study site.

If appropriate, any additional requirements imposed by the IEC will be followed. Amendments to the study documents will be notified to, or approved by, the IEC before implementation, if applicable.

19.3 Subject information and consent procedure

Before any clinical study-related activities are performed, the investigator (or authorized designee) must review the informed consent form and explain the study to potential study subjects. The

investigator must ensure that the subject is fully informed about the aims, procedures, potential risks, any discomforts, and expected benefits of the clinical trial. Before consenting, the subject must be left with ample time to consider and ask questions. It must be emphasized that participation is voluntary and that the subject has the right to withdraw from the clinical trial at any time and for any reason without prejudice. The subject must then sign and date the consent form prior to the conduct of any study procedures. The investigator must sign and date the informed consent as well.

A copy of the subject information and informed consent form will be given to the subjects for their records. The original signed informed consent form must remain in each subject's binder and must be available for verification by study monitors, auditors or inspectors at any time. The rights and welfare of the study subjects will be protected by emphasizing to them that the quality of their medical care will not be adversely affected if they decline to participate in this clinical study.

If amendments to the final study protocol affect the subject's participation in the clinical trial (e.g. a change in any procedure), the subject information and informed consent form must be updated to incorporate this modification, and subjects must agree to sign the amended form indicating that they re-consent to participate in the clinical trial.

19.4 Insurance coverage

Insurance coverage for damages emerging from the clinical trial will be provided according to applicable legal requirements. During the informed consent procedure, the investigator must inform the subject accordingly. Insurance details will be provided to the subject within the subject information sheet.

19.5 Submission to authorities

Documents required for the study application will be submitted to the responsible competent authority (CA). The study will not start until this authority has authorized the study. Amendments to the study protocol or to any other documents that must be reviewed by the CA will also be submitted to the CA in accordance with the regulatory requirements. If applicable, approval of the amendment must be awaited before implementing any changes.

19.6 Subject confidentiality

The study protocol, documentation, data, and all other information generated will be held in strict confidence. No information concerning the clinical trial or the data will be released to any unauthorized third party without prior written approval of the sponsor.

Personal subject data will be kept confidential in compliance with the European Data Protection Directive [6] and other applicable national requirements.

The investigator must ensure that the pseudonymity of study subjects will be maintained and that their identities are protected from unauthorized parties. On eCRFs, compensation documentation, or

any other documents submitted to the sponsor, subjects must be identified only by their identification codes; it is not allowed to use their names, addresses, telephone numbers, or similar information. The investigator will keep the original of the Subject Identification Log (including complete name and date of birth of each subject) in his/her file. The investigator must maintain these documents in strict confidence.

To allow compliance with GCP, all subjects will be asked for consent regarding the access to their personal clinical study-related data for monitoring, audits, and inspections as well as regarding transmission and storage of their pseudonymous data; a respective statement will be part of the informed consent form. Professionals getting access to source data for monitoring, audits and inspections are bound to preserve strict confidentiality.

20 Criteria for premature termination of the trial and criteria for premature closing a study center

20.1 Premature termination of the clinical study

The sponsor reserves the right to terminate the study for any reason (e.g., safety, new data on the risk/benefit, ethical or administrative reasons, insufficient patient recruitment or high number of early terminations). The sponsor will notify all investigators outlining the reasons for the termination of the study. The sponsor will provide the investigators with instructions if assessments beyond the regular per-protocol procedures should be necessary. If the study is prematurely terminated, the sponsor or sponsor’s designee will promptly inform the CA of the termination and its reason(s); the investigator, sponsor, or sponsor’s designee will promptly inform the IEC, as specified in applicable regulations.

In addition, if the study medication is associated with unacceptable AEs (incidence or severity of AEs indicate a potential health hazard to study subjects) the study recruitment may be suspended or stopped or the study terminated completely upon recommendation of the DEC.

If recruitment is suspended or stopped, all ongoing subjects will proceed study participation as planned according to this protocol. If the whole study is terminated all still ongoing subjects will complete the withdrawal assessments and the corresponding documentation according to section 12.

The IEC and the competent authority will be informed accordingly.

20.2 Premature termination of the clinical study at an individual center

Both the sponsor and the investigator reserve the right to terminate the clinical study at a study center at any time. In terminating the clinical study at a study site, the sponsor and the investigator(s) will assure that adequate consideration is given to the information and protection of the clinical study subjects.

The sponsor may declare to stop or suspend recruitment or terminate the clinical study at a site in particular for the following reasons:

- The center appears unable to include patients in the clinical study.
- The investigator fails to comply with the requirements of the clinical study protocol, with GCP standards, other pertinent quality standards or applicable regulatory requirements, thus endangering the safety for the clinical study subject or the clinical study integrity.

If the sponsor decides to terminate or suspend a clinical study at a study center, the sponsor must promptly inform the investigator and, if necessary, the institution. If the clinical study is terminated prematurely at a study center, the sponsor or designee will inform the IEC and CA of the termination and the reason(s) for the termination according to local rules.

If the investigator terminates or suspends a clinical study on his/her initiative with or without prior agreement of the sponsor, the investigator must promptly inform the sponsor and should provide a detailed written explanation of the termination or suspension. The sponsor or designee will promptly inform the CA and IEC and provide them with a written explanation of the termination of the study center.

21 Study protocol, documentation and archiving of data

21.1 Amendments to the protocol

Any modification to the protocol which may impact the conduct of the study or may affect subject safety, including changes of study objectives, study design, patient population, sample sizes, study procedures, or significant administrative aspects will require a formal amendment to the protocol. Such amendments have to be discussed and signed by the sponsor, the coordinating investigator, and the principal investigator(s) prior to implementation, and approved by the IEC prior to implementation and notified to or approved by the CA in accordance with local regulations.

Substantial amendments will be issued either as a separate document that details the changes or as an amended protocol that incorporates all changes, or both.

Amendments that might have an impact on study procedures to be performed and/or the well-being of the subjects require an updated informed consent form that is to be signed by all subjects enrolled in the study who are affected by the amendment.

Non-substantial changes, e.g. minor corrections of administrative nature and/or clarifications without substantial impact on study design, conduct, and risks are considered editorial changes. These will be documented in a formal amendment or a Note to File, which both will be signed by the sponsor and principal investigators. The IEC and competent regulatory authorities need not to

be notified of such minor corrections.

The principal investigator(s) will not implement any deviation from, or changes to the protocol without agreement by the sponsor and prior review and documented approval/favorable opinion from the competent authority and IEC of an amendment regarded as substantial, except where necessary to eliminate immediate hazards to trial subjects, or when the changes involve only minor logistical or administrative aspects of the trial (e.g. change in monitor(s), change in telephone numbers).

21.2 Protocol deviations

A protocol deviation is a failure to follow the requirements of the protocol, intentionally or unintentionally. Requests for deviations and reports of deviations will be provided to the IEC if the deviation affects subject’s rights, safety and well-being, or the scientific integrity of the clinical trial.

Under emergency circumstances deviations from the protocol may proceed without prior approval by the sponsor and favorable opinion of the IEC, if the rights, safety and well-being of human subjects need to be protected. Such deviations will be documented and reported to the sponsor and the IEC as soon as possible in accordance with national regulations.

All protocol deviations will be listed and if the subjects concerned will be evaluable for analysis will be discussed in a data review meeting prior to the statistical analysis.

21.3 Archiving of data

All essential documents at the study center or the sponsor should be retained

- until at least 2 years after the last approval of a marketing application in an ICH region,
- until at least 10 years after the study end or premature termination,
- until there are no pending or contemplated marketing applications in an ICH region,
- or until at least 2 years have elapsed since the formal discontinuation of clinical development of the test medication.

Patient identification codes have to be retained according to ICH GCP or for at least 15 years after the completion or discontinuation of the study, whatever period is longer.

The final report will be kept for another 5 years after the drug has been taken from the market according to legal stipulations. The documents should however, be archived for a longer period if required by the applicable regulatory authorities or if agreed with the sponsor. It is the responsibility of the sponsor to inform the investigators when these documents are no longer need to be retained.

The investigator(s) will be provided with a study file where all study related documents will be

archived.

The medical files of study subjects must be retained in accordance with local legislation and in accordance with the maximum period of time permitted by the hospital, institution or private practice.

22 Data collection, monitoring and quality assurance

22.1 Data collection

A web-based standardized electronic case report form (eCRF) will be used to document the subjects' data during the course of the study. The eCRF is designed to accommodate the specific features of the study design. All data obtained after the patient has given informed consent must be recorded in the eCRF. The investigator will assure that all data are entered promptly, completely, and accurately according to the eCRF instructions, and conform to source documents. Data for all subjects screened for study inclusion have to be documented on a respective form.

Only investigators and authorized designees are allowed to make entries in the eCRF. This will be regulated by appropriate reading and writing access. Completed eCRFs per visit, must be electronically signed by the investigator or authorized designee. Any change or addition will be recorded by an audit trail system.

The investigator or designee has to carefully answer queries issued by Data Management in the eCRF.

It is the investigators' obligation to assure documentation of all relevant data in the patient's file, such as medical history, concomitant diseases, date of study enrolment, visit dates, results of examinations, administrations of medication, and AEs.

22.2 Data management procedures

All data management activities will be conducted by the sponsor's designee following their SOPs. The database will be built by the sponsor's designee.

Details on data handling will be described in the Data Management Plan. The sponsor's designee will handle the data cleaning process, including logical check, and query processes. Computerized validation check programs on completeness, correctness, plausibility (such as range checks, cross-checks) will verify the data according to the Data Validation Plan. All identified discrepancies will be addressed to the investigator.

Regarding self-evident corrections (such as spelling and header corrections) if applicable, investigator's agreement will be obtained, that these will be made by the Data Manager and documented in the audit trail.

The database will be hard locked after all the changes following the data review meeting have been

done and the database is considered complete and accurate. All changes will be tracked (audit trail). Sponsor approval prior to database hard lock is mandatory.

22.3 Monitoring procedures

The extent of monitoring and source data verification will be specified in a monitoring plan.

Before study initiation at a center, the sponsor or designee will discuss the protocol and the eCRF with the investigators and their staff. The site may not enroll any subject before this initiation visit. During the study further monitoring visits will be performed according to ICH GCP, the sponsor's designee standard operating procedures (SOPs), and local regulations. The eCRF entries will be reviewed against source data for adherence to the study protocol and ICH GCP, as well as for completeness, accuracy and consistency of data. Additionally, the monitor will check the progress of enrolment, and will ensure that study medication is being stored, dispensed and accounted for according to specifications. Key study personnel must be available to assist the monitor during these visits.

The investigators must permit the monitors access to those portions of the patient's primary medical records, which directly concern this study. Throughout the study, all collected data will only be identified by the subject numbers. Accordingly, only subject numbers will be used in all data analyses.

It is the investigators' obligation to assure documentation of all relevant data in the patient's file, such as medical history and concomitant diseases, date of study enrolment, visit dates, results of examinations, administrations of medication, and AEs.

In case of electronic source data, access must be allowed or dated print-outs must be available prior to the monitoring visits.

22.4 Audits and inspections

During the study in-house and on-site audits may be performed by independent auditors. Audits of clinical research activities will be performed in accordance with corresponding SOPs to evaluate compliance with the principles of GCP.

Regulatory authorities may wish to conduct an inspection. If an inspection is requested, the investigator must inform the sponsor or designee immediately.

The investigator must allow the persons performing the audits or inspections access to source data and documents and will answer any questions.

22.5 Study report

After the end of the clinical study (defined as data base lock) and data evaluation a fully integrated clinical study report will be prepared. It will be submitted to the all investigators for

acknowledgement and signature.

According to the respective regulations, a summary of the results of the clinical study will be submitted to the IEC and CA within 1 year after the end of the clinical study.

22.6 Publications

By signing the study protocol, the investigators agree that the sponsor has the right to use the clinical study results for registration purposes, internal presentation, scientific meetings and -if the product is approved - for promotion. The investigator acknowledges that the results of the study may constitute intellectual property and/or trade secrets of the sponsor.

If the sponsor plans to publish results from the present clinical study, the sponsor will contact investigators and other contributing parties to mutually agree on qualification for authorship, based on the actual contributions of the parties involved in clinical study design, data analysis, clinical conduct, and writing of manuscripts.

If an investigator is invited to act as co-author for the publication, he/she can only be mentioned in the manuscript if giving permission. The sponsor will send the manuscript to the investigator for review and comments, and the investigator has the right to have his/her interpretation of the data properly represented in the publication. If the agreed timeline for review of a draft publication to the sponsor is not adhered to by the investigator, the sponsor holds the right to publish.

The sponsor acknowledges the right and interest of the investigator to publish results of the clinical study. Therefore, the investigator will submit to the sponsor all proposed publications and presentations by the investigator or his personnel and associates resulting from or related to this study for review at least 30 working days before submission for publication or presentation. The sponsor reserves the right to refuse or restrict publication only to the extent such publication would affect sponsor’s trade secrets or right to claim intellectual property protection.

23 Study periods

Study start:

The study will start with the first patient signing informed consent.

Estimated Start: April 2016

Study treatment period:

The treatment period begins as soon as the first subject received study medication until the last subject received study medication.

Study follow up period:

The follow up period starts as soon as the last subject received study medication.

Study completion (last subject last visit):

The study is completed as soon as the last subject:

- completes the study by undergoing the final study visit Day 28
- is withdrawn from the study (and undergoes the withdrawal assessment).

Estimated completion: November 2016

Study end:

The study ends as soon as the study database is locked.

Estimated End: January 2017

Estimated Recruitment Period: 6 months

24 References

1. Ganz T, Nemeth E. Hepcidin and iron homeostasis. Biochim Biophys Acta 2012; 1823:1434-43
2. Ramos E, Kautz L, Rodriguez R et al. Evidence for distinct pathways of hepcidin regulation by acute and chronic iron loading in mice. Hepatology 2011; 53:1333-41
3. Andrews NC. Closing the Iron Gate;, N Engl J Med 2012; 366(4), 376-77
4. Nemeth E, Valore EV, Territo M et al.: Hepcidin, a putative mediator of anemia of inflammation, is a type II acute-phase protein. Blood 2003; 101:2461-63
5. Cawello W. Parameters for Compartment-free Pharmacokinetics, Shaker Verlag GmbH, Aachen, 1999
6. European Parliament. Directive 95/46/EC of the European Parliament and of the Council of 24 October 1995 on the protection of individuals with regard to the processing of personal data and on the free movement of such data. [Online]. Available from: http://eur-lex.europa.eu/ 1995

25 Approval and signatures

Protocol agreed to by sponsor:

Louis Matis

Sponsor's signatory name (print)

25-July-2016

Sponsor's signatory signature | Date

Protocol agreed to by coordinating investigator:

Coordinating Investigator Name (print)

Coordinating investigator signature | Date

Principal Investigator Agreement Page for the protocol

I agree:

- To assume responsibility for the proper conduct of the clinical study at this site, and to conduct the study in compliance with national law, the current version of the Declaration of Helsinki, the European GCP-guidelines, the present study protocol including its amendments, and with any other study conduct procedures provided by the sponsor or authorized representatives.
- Not to implement any deviations from or changes to the protocol (including protocol amendments) without agreement from the sponsor and prior review and favorable opinion from the Ethics Committee and approval from the Competent Authority, if applicable, except where necessary to eliminate an immediate hazard to the subject(s), or for administrative aspects of the clinical study (where permitted by all applicable regulatory requirements).
- That I am familiar with the appropriate use of the investigational medicinal product as described in this protocol and any other information provided by the sponsor including, but not limited to, the current Investigator's Brochure or equivalent document provided by the sponsor.
- To ensure that all persons assisting me with the clinical study are adequately informed about the investigational medicinal product and of their study-related duties and functions.
- That I have been informed that certain regulatory authorities require the sponsor to obtain and supply details about the investigator's ownership interest in the sponsor or the study product, and more generally about his/her financial ties with the sponsor. The sponsor will use and disclose the information solely for the purpose of complying with regulatory requirements.

____

Principal investigator name (print)

____________________________________ ________________

Principal investigator signature Date
