# Supplementary figures and images for: First-in-human Phase I studies of PRS-080#22, a hepcidin antagonist, in healthy volunteers and patients with chronic kidney disease undergoing hemodialysis
Source: PLoS One. 2019 Mar 27;14(3):e0212023. doi: 10.1371/journal.pone.0212023 (PMC6436791; doi:10.1371/journal.pone.0212023)

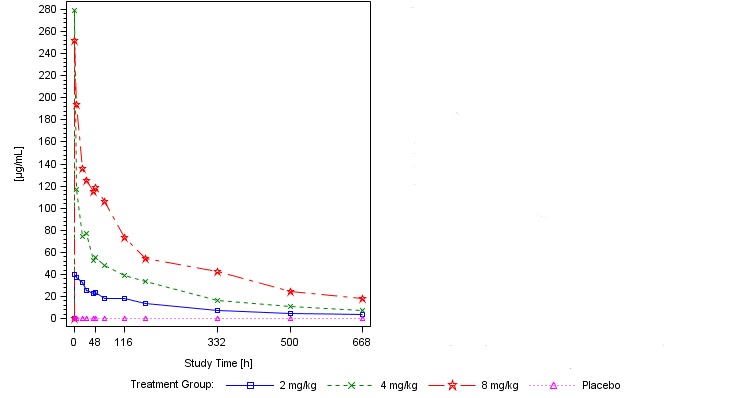

Supplement: S1 Fig — (JPG) [file pone.0212023.s003.JPG]

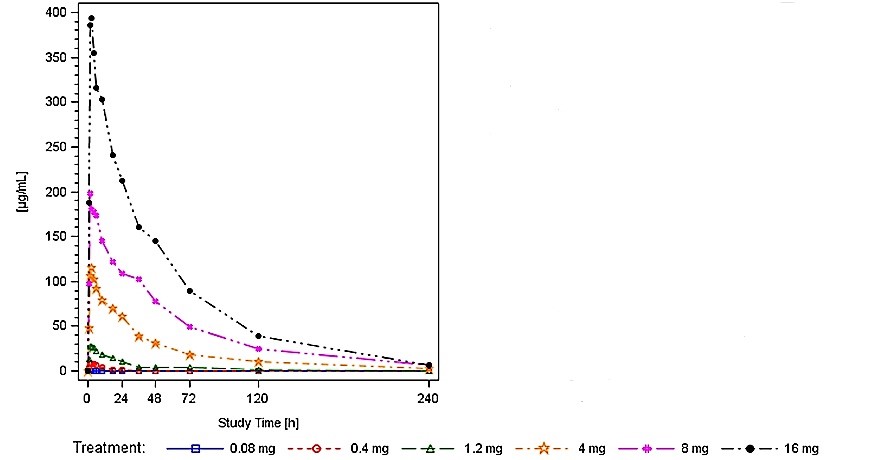

Supplement: S2 Fig — (JPG) [file pone.0212023.s004.jpg]

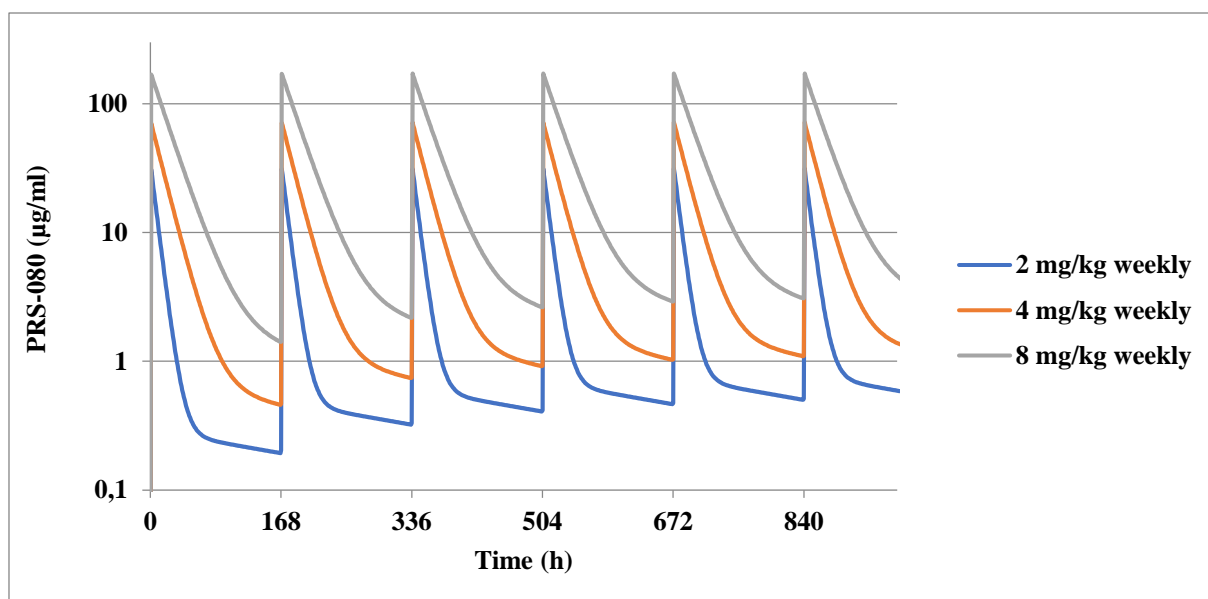

Supplement: S3 Fig — (PDF) [file pone.0212023.s005.pdf]

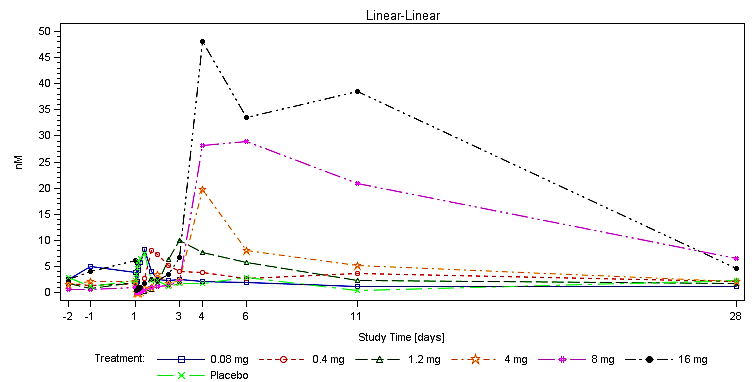

Supplement: S4 Fig — (PNG) [file pone.0212023.s006.png]

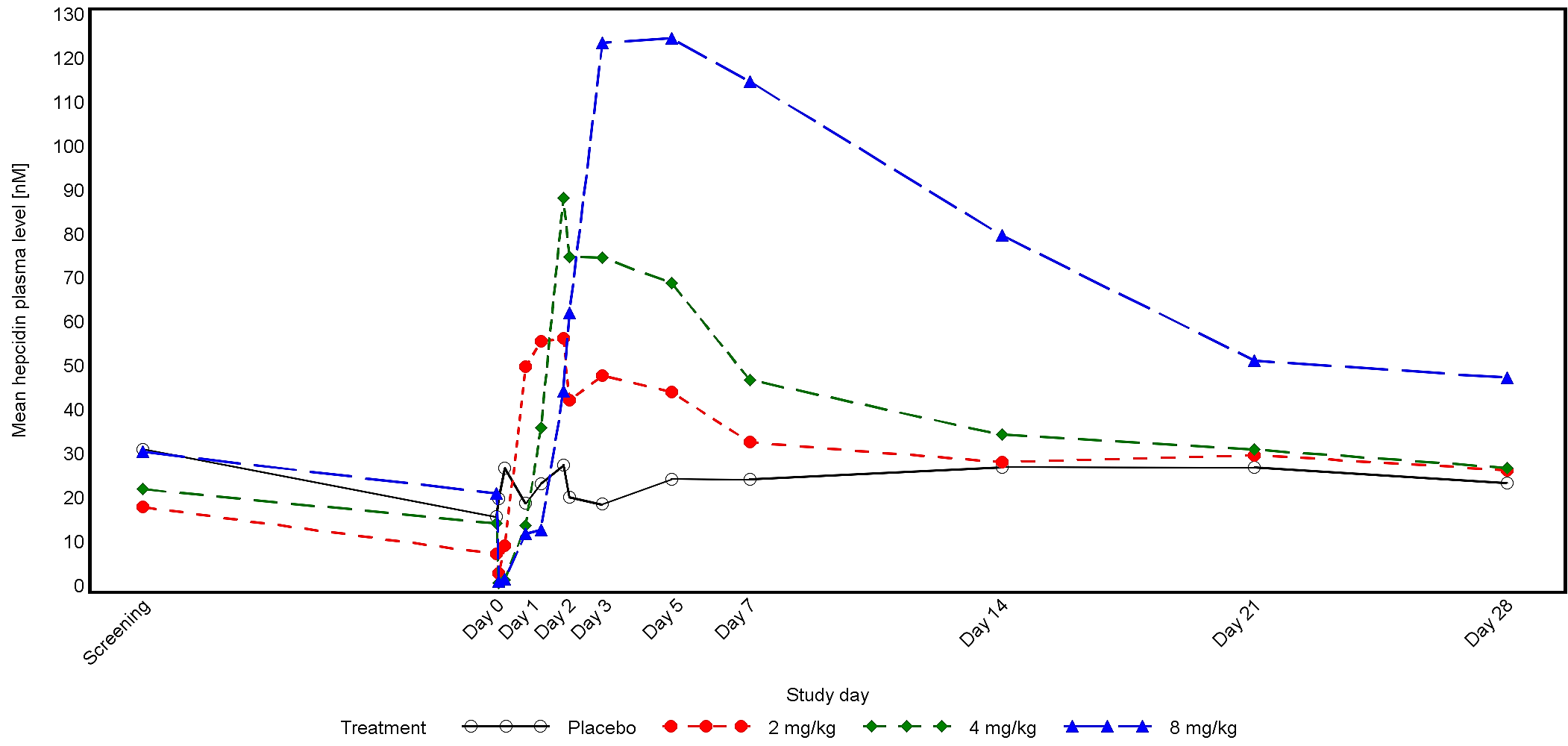

Supplement: S5 Fig — (PNG) [file pone.0212023.s007.png]
