# Supplementary material for: First-in-human Phase I studies of PRS-080#22, a hepcidin antagonist, in healthy volunteers and patients with chronic kidney disease undergoing hemodialysis
Source: PLoS One. 2019 Mar 27;14(3):e0212023. doi: 10.1371/journal.pone.0212023 (PMC6436791; doi:10.1371/journal.pone.0212023)
Supplement: S1 Table — (PDF) [file pone.0212023.s008.pdf]

Parameter	Mean	SD*	CV%*	Median	Range
Age (years)	36.1	9.6	26.6	36.5	18.0-58.0
Height (cm)	177.2	7.1	4.0	178.0	157.0-192.0
Weight (kg)	76.8	9.3	18.3	80.0	60.1-90.0
BMI (kg/m^2)	24.5	2.0	8.2	24.6	20.4-28.8
Gender	All volunteers were male				
Race	All volunteers were caucasian				

*SD: standard deviation, CV: coefficient of variation
