# Supplementary material for: First-in-human Phase I studies of PRS-080#22, a hepcidin antagonist, in healthy volunteers and patients with chronic kidney disease undergoing hemodialysis
Source: PLoS One. 2019 Mar 27;14(3):e0212023. doi: 10.1371/journal.pone.0212023 (PMC6436791; doi:10.1371/journal.pone.0212023)
Supplement: S2 Table — (PDF) [file pone.0212023.s009.pdf]

Parameter	Mean	SD	1st quartile	median	3rd quartile	Range
Age (years)	55.4	14.1	47.0	60.0	65.0	28-72
Height (cm)	170.0	9.2	163.0	170.0	178.0	150-185
Weight (kg)	77.05	14.43	66.3	77.9	84.8	50.0-107.4
BMI (kg/m^2)	26.6	4.9	22.7	25.1	30.5	18.3-35.5
Albumine (g/dL)	4.2	0.4	3.9	4.1	4.3	3.6-4.9
Creatinine (mg/dL)	8.6	2.8	6.6	8.4	10.5	4.2-14.5
Iron (µg/dL)	85.5	39.0	57.5	72.0	106.0	29-175
Ferritin (ng/mL)	732.7	347.1	438.0	707.5	900.0	265-1469
Gender	Male: 17 (70.8%)			Female: 7 (29.2%)		
Race	Caucasian: 23 (95.8%)			Other: 1 (4.2%)		
