# Supplementary material for: First-in-human Phase I studies of PRS-080#22, a hepcidin antagonist, in healthy volunteers and patients with chronic kidney disease undergoing hemodialysis
Source: PLoS One. 2019 Mar 27;14(3):e0212023. doi: 10.1371/journal.pone.0212023 (PMC6436791; doi:10.1371/journal.pone.0212023)
Supplement: S3 Table — (PDF) [file pone.0212023.s010.pdf]

Free Anticalin

Parameter	Statistics	0.08 mg (N=6)	0.4 mg (N=6)	1.2 mg (N=6)	4 mg (N=6)	8 mg (N=6)	16 mg (N=6)
AUCz [h*µg/mL]	n	6	6	6	6	6	6
	Mean	14.275	134.183	874.805	4955.308	10841.729	19609.450
	SD	1.7972	69.8435	527.5168	2365.6970	3932.4456	6324.7357
	CV%	12.6	52.1	60.3	47.7	36.3	32.3
	Minimum	11.69	81.39	323.70	2351.31	6082.81	8591.28
	Median	14.230	111.479	758.020	4698.254	10788.812	20407.231
	Maximum	16.35	265.40	1739.71	7660.54	15477.92	26364.24
	Geom. Mean	14.179	122.255	748.160	4459.664	10216.402	18496.712
	Geom. CV%	12.9	47.5	68.7	55.0	40.0	42.3
AUC0-inf [h*µg/mL]	n	5	2	5	6	5	5
	Mean	17.772	215.065	883.809	5144.200	11052.073	22383.549
	SD	2.3973	73.4732	686.1691	2585.1175	4961.6654	4363.5218
	CV%	13.5	34.2	77.6	50.3	44.9	19.5
	Minimum	14.18	163.11	333.92	2372.65	6128.16	17327.70
	Median	18.167	215.065	583.745	4727.800	8912.955	20810.650
	Maximum	20.78	267.02	2040.47	8249.13	17126.66	28580.96
	Geom. Mean	17.637	208.696	718.240	4580.755	10185.439	22051.377
	Geom. CV%	14.1	35.9	78.9	57.9	47.6	19.4
Cmax [µg/mL]	n	6	6	6	6	6	6
	Mean	0.7087	9.1283	29.2167	117.6833	205.0000	404.5000
	SD	0.58488	0.94529	5.65665	27.80593	39.91491	84.41268
	CV%	82.5	10.4	19.4	23.6	19.5	20.9
	Minimum	0.225	7.720	21.500	81.900	159.000	305.000
	Median	0.4870	9.1750	31.4000	121.5000	196.5000	410.5000
	Maximum	1.550	10.300	35.700	158.000	277.000	533.000
	Geom. Mean	0.5111	9.0866	28.7270	114.8727	202.0090	397.1909
	Geom. CV%	112.8	10.6	20.8	24.7	18.6	21.2
Tmax [h]	n	6	6	6	6	6	6
	Mean	2.0000	2.1723	2.8362	4.0000	2.8333	2.3333
	SD	0.00000	0.41386	0.75356	2.96648	1.60208	0.51640
	CV%	0.0	19.1	26.6	74.2	56.5	22.1
	Minimum	2.000	2.000	2.000	2.000	2.000	2.000
	Median	2.0000	2.0000	3.0000	3.0000	2.0000	2.0000
	Maximum	2.000	3.017	4.000	10.000	6.000	3.000

Parameter	Statistics	0.08 mg (N=6)	0.4 mg (N=6)	1.2 mg (N=6)	4 mg (N=6)	8 mg (N=6)	16 mg (N=6)
t1/2 [h]	n	5	2	5	6	5	5
	Mean	113.243	57.000	63.772	46.295	51.304	42.365
	SD	15.6437	58.8928	18.4769	14.5432	25.9799	13.5324
	CV%	13.8	103.3	29.0	31.4	50.6	31.9
	Minimum	99.50	15.36	40.12	32.64	31.25	33.88
	Median	108.323	57.000	64.432	39.017	34.695	36.452
	Maximum	138.39	98.64	90.25	67.77	87.34	66.23
	Geom. Mean	112.430	38.920	61.589	44.563	46.529	40.965
	Geom. CV%	13.3	215.4	30.6	30.3	51.5	28.1
Vss [L/kg]	n	5	2	5	6	5	5
	Mean	0.59097	0.09209	0.08941	0.04645	0.04704	0.04810
	SD	0.116070	0.072267	0.038254	0.015609	0.009330	0.003070
	CV%	19.6	78.5	42.8	33.6	19.8	6.4
	Minimum	0.4211	0.0410	0.0581	0.0320	0.0331	0.0443
	Median	0.60365	0.09209	0.07026	0.04158	0.04793	0.04690
	Maximum	0.7196	0.1432	0.1531	0.0767	0.0572	0.0520
	Geom. Mean	0.58115	0.07662	0.08395	0.04467	0.04623	0.04802
	Geom. CV%	21.2	108.9	39.7	30.0	21.5	6.4

Total Anticalin

Parameter	Statistics	0.08 mg (N=6)	0.4 mg (N=6)	1.2 mg (N=6)	4 mg (N=6)	8 mg (N=6)	16 mg (N=6)
AUCτ [h*µg/mL]	n	6	6	6	6	6	6
	Mean	141.5	680.6	2264.3	7490.5	15065.8	25572.2
	SD	15.29	105.59	167.47	730.35	2495.62	4074.59
	CV%	10.8	15.5	7.4	9.8	16.6	15.9
	Minimum	122	550	2060	6380	12363	17664
	Median	140.0	674.3	2298.8	7750.4	14195.3	26704.1
	Maximum	162	867	2509	8281	18295	29110
	Geom. Mean	140.8	674.1	2259.1	7459.7	14898.8	25253.3
	Geom. CV%	10.8	15.1	7.4	10.1	16.4	18.3
AUC0-inf [h*µg/mL]	n	6	6	6	6	6	5
	Mean	161.6	761.0	2570.1	8308.1	17131.9	27659.7
	SD	17.41	162.69	255.43	922.14	2836.67	4378.82
	CV%	10.8	21.4	9.9	11.1	16.6	15.8
	Minimum	140	599	2286	6744	14145	20029
	Median	163.2	706.6	2581.2	8617.3	16436.6	29183.0
	Maximum	183	1069	2975	9164	20601	30926
	Geom. Mean	160.8	748.2	2559.7	8262.6	16939.5	27339.3
	Geom. CV%	10.9	19.8	9.9	11.7	16.5	17.9
Cmax [µg/mL]	n	6	6	6	6	6	6
	Mean	2.092	10.590	33.900	120.383	246.333	366.167
	SD	0.3162	1.6193	4.3945	19.5469	56.7615	40.8726
	CV%	15.1	15.3	13.0	16.2	23.0	11.2
	Minimum	1.51	9.13	28.80	92.30	176.00	287.00
	Median	2.195	9.980	33.900	121.500	239.500	376.500
	Maximum	2.36	13.50	39.90	143.00	322.00	401.00
	Geom. Mean	2.069	10.495	33.663	119.012	240.882	364.050
	Geom. CV%	16.8	14.5	13.0	16.9	23.6	12.2
Tmax [h]	n	6	6	6	6	6	6
	Mean	2.8333	3.3333	2.6695	3.6667	4.3333	3.0000
	SD	0.40825	1.63299	0.81791	3.14113	2.80476	0.63246
	CV%	14.4	49.0	30.6	85.7	64.7	21.1
	Minimum	2.000	2.000	2.000	2.000	3.000	2.000
	Median	3.0000	3.0000	2.5000	2.5000	3.0000	3.0000
	Maximum	3.000	6.000	4.000	10.000	10.000	4.000

Parameter	Statistics	0.08 mg (N=6)	0.4 mg (N=6)	1.2 mg (N=6)	4 mg (N=6)	8 mg (N=6)	16 mg (N=6)
t1/2 [h]	n	6	6	6	6	6	5
	Mean	81.223	70.477	80.253	73.112	79.643	80.202
	SD	8.7443	27.6715	10.3387	8.9319	9.6881	11.5771
	CV%	10.8	39.3	12.9	12.2	12.2	14.4
	Minimum	66.62	21.75	68.52	60.33	71.82	62.95
	Median	82.728	73.791	80.964	75.813	75.740	81.950
	Maximum	92.60	104.87	91.95	81.84	98.25	92.55
	Geom. Mean	80.813	63.770	79.691	72.638	79.194	79.494
	Geom. CV%	11.2	59.8	13.1	12.7	11.4	15.2
Vss [L/kg]	n	6	6	6	6	6	5
	Mean	0.05622	0.05416	0.05132	0.04784	0.05330	0.06457
	SD	0.007985	0.009765	0.004079	0.005578	0.009250	0.014573
	CV%	14.2	18.0	7.9	11.7	17.4	22.6
	Minimum	0.0466	0.0363	0.0436	0.0407	0.0406	0.0476
	Median	0.05439	0.05549	0.05245	0.04766	0.05522	0.06171
	Maximum	0.0688	0.0640	0.0548	0.0549	0.0652	0.0869
	Geom. Mean	0.05577	0.05331	0.05118	0.04757	0.05260	0.06331
	Geom. CV%	14.0	20.5	8.4	11.8	18.1	22.4

AUC_z: AUC by trapezoidal rule, 0 – last quantifiable sample, AUC_{-inf}: AUC by trapezoidal rule, extrapolated to infinity, C_{max}: observed maximal concentration, T_{max}: time of observed maximum concentration, $t_{1/2}$: terminal half-life, V_{ss}: volume of distribution during the terminal phase
