# Supplementary material for: First-in-human Phase I studies of PRS-080#22, a hepcidin antagonist, in healthy volunteers and patients with chronic kidney disease undergoing hemodialysis
Source: PLoS One. 2019 Mar 27;14(3):e0212023. doi: 10.1371/journal.pone.0212023 (PMC6436791; doi:10.1371/journal.pone.0212023)
Supplement: S4 Table — (PDF) [file pone.0212023.s011.pdf]

Free Anticalin

Parameter	Statistics	Treatment 2 mg/kg	4 mg/kg	8 mg/kg
AUC0-t [h*µg/mL]	n	6	6	6
	Mean	513.190	1876.459	4432.260
	SD	182.9286	919.2034	1470.3440
	CV%	35.6	49.0	33.2
	Minimum	278.01	1031.22	2960.93
	Median	514.584	1663.782	4088.269
	Maximum	759.78	3498.85	6778.05
	Geom. n	6	6	6
	Geom. Mean	484.267	1713.093	4242.349
	Geom. SD	1.4661	1.5820	1.3782
	Geom. CV%	39.7	48.4	32.9
AUC0-inf [h*µg/mL]	n	5	6	5
	Mean	489.835	1921.713	4743.166
	SD	166.4978	918.1360	1496.5637
	CV%	34.0	47.8	31.6
	Minimum	289.64	1111.11	3051.44
	Median	537.182	1688.588	4409.445
	Maximum	708.23	3558.37	6863.88
	Geom. n	5	6	5
	Geom. Mean	466.056	1765.027	4558.198
	Geom. SD	1.4329	1.5541	1.3713
	Geom. CV%	37.2	46.3	32.4
Cmax [µg/mL]	n	6	6	6
	Mean	29.72	174.1	164.0
	SD	6.8221	154.69	41.718
	CV%	23.0	88.8	25.4
	Minimum	19.3	40.1	118
	Median	29.80	112.0	158.0
	Maximum	38.1	458	213
	Geom. n	6	6	6
	Geom. Mean	29.01	128.4	159.6
	Geom. SD	1.2786	2.3441	1.2913
	Geom. CV%	25.0	103.3	26.0
Tmax [h]	n	6	6	6
	Mean	1.6888	1.0110	1.6833
	SD	1.64669	0.01704	1.62515
	CV%	97.5	1.7	96.5
	Minimum	1.000	1.000	1.000
	Median	1.0250	1.0000	1.0085
	Maximum	5.050	1.033	5.000
	Geom. SD	1.92436	1.01691	1.91535
	Geom. CV%	73.1	1.7	72.5
t1/2 [h]	n	5	6	5
	Mean	293.201	259.676	231.189
	SD	77.0581	70.4599	99.5855
	CV%	26.3	27.1	43.1
	Minimum	235.68	161.25	163.45
	Median	252.576	253.226	192.274
	Maximum	420.54	369.17	407.38
	Geom. n	5	6	5
	Geom. Mean	286.080	251.551	218.080
	Geom. SD	1.2721	1.3231	1.4313
	Geom. CV%	24.4	28.6	37.0
Vss [L/kg]	n	5	6	5
	Mean	0.54779	0.21398	0.11579
	SD	0.234089	0.179132	0.051186
	CV%	42.7	83.7	44.2
	Minimum	0.2821	0.0731	0.0708
	Median	0.56756	0.13814	0.09013
	Maximum	0.8729	0.5267	0.1969
	Geom. n	5	6	5
	Geom. Mean	0.50653	0.16309	0.10790
	Geom. SD	1.570466	2.205106	1.505674
	Geom. CV%	47.5	93.2	42.7

Total Anticalin

Parameter	Statistics	Treatment: 2 mg/kg	4 mg/kg	8 mg/kg
AUC0-t [h*µg/mL]	n	6	6	6
	Mean	7141.09	16997.51	33961.94
	SD	1972.195	5117.045	8361.271
	CV%	27.6	30.1	24.6
	Minimum	5040.9	8250.7	28423.3
	Median	6982.28	17786.22	31121.63
	Maximum	9459.0	23611.7	50814.6
	Geom. n	6	6	6
	Geom. Mean	6911.75	16204.60	33265.05
	Geom. SD	1.325	1.435	1.236
	Geom. CV%	28.7	37.3	21.5
AUC0-inf [h*µg/mL]	n	6	4	6
	Mean	8507.06	19839.83	41589.51
	SD	2185.379	6987.032	11009.413
	CV%	25.7	35.2	26.5
	Minimum	6139.8	9841.0	34927.4
	Median	8612.93	21692.78	36489.19
	Maximum	11122.1	26132.8	63416.3
	Geom. n	6	4	6
	Geom. Mean	8267.91	18651.44	40601.11
	Geom. SD	1.302	1.545	1.257
	Geom. CV%	26.8	45.7	23.2
Cmax [µg/mL]	n	6	6	6
	Mean	44.67	281.9	255.0
	SD	13.768	231.35	116.73
	CV%	30.8	82.1	45.8
	Minimum	28.9	61.1	137
	Median	42.50	183.0	238.0
	Maximum	69.4	681	434
	Geom. n	6	6	6
	Geom. Mean	43.06	211.6	233.3
	Geom. SD	1.3390	2.3390	1.5913
	Geom. CV%	29.8	102.9	49.1
Tmax [h]	n	6	6	6
	Mean	4.8998	1.6777	4.9750
	SD	7.72677	1.64382	7.93151
	CV%	157.7	98.0	159.4
	Minimum	1.000	1.000	1.000
	Median	1.0330	1.0000	1.0085
	Maximum	20.333	5.033	20.833
	Geom. SD	3.54426	1.92941	3.59537
	Geom. CV%	199.0	73.5	203.5
t1/2 [h]	n	6	4	6
	Mean	265.135	239.656	275.823
	SD	59.1592	38.9693	66.4025
	CV%	22.3	16.3	24.1
	Minimum	192.04	203.43	225.11
	Median	257.574	230.860	246.640
	Maximum	368.53	293.47	404.05
	Geom. n	6	4	6
	Geom. Mean	259.947	237.397	270.190
	Geom. SD	1.2408	1.1702	1.2385
	Geom. CV%	21.8	15.8	21.6
Vss [L/kg]	n	6	4	6
	Mean	0.08866	0.07563	0.07328
	SD	0.028216	0.047482	0.019824
	CV%	31.8	62.8	27.1
	Minimum	0.0624	0.0394	0.0519
	Median	0.08163	0.05906	0.06750
	Maximum	0.1222	0.1450	0.1096
	Geom. n	6	4	6
	Geom. Mean	0.08501	0.06661	0.07129
	Geom. SD	1.372967	1.749253	1.285246
	Geom. CV%	32.5	60.6	25.5

$AUC_{0\text{-}t}$: AUC by trapezoidal rule, 0 – last quantifiable sample, $AUC_{0\text{--}inf}$: AUC by trapezoidal rule, extrapolated to infinity, C_{max}: observed maximal concentration, T_{max}: time of observed maximum concentration, $t_{1/2}$: terminal half-life, V_{ss}: volume of distribution during the terminal phase
