# Supplementary material for: First-in-human Phase I studies of PRS-080#22, a hepcidin antagonist, in healthy volunteers and patients with chronic kidney disease undergoing hemodialysis
Source: PLoS One. 2019 Mar 27;14(3):e0212023. doi: 10.1371/journal.pone.0212023 (PMC6436791; doi:10.1371/journal.pone.0212023)
Supplement: S5 Table — (PDF) [file pone.0212023.s012.pdf]

Time	CKD patients						Healthy Volunteers							
	2mg		4mg		8mg		1.2mg		4mg		8mg		16 mg	
	Iron µg/dl	TSAT %	Iron/ µg/dl	TSAT %	Iron µg/dl	TSAT %	Iron µg/dl	TSAT %	Iron µg/dl	TSAT %	Iron µg/dl	TSAT %	Iron µg/dl	TSAT %
0 h	60.2	23.3	79.5	28.7	104.3	32.8	21.8	37.7	20.1	36.0	21.5	40.7	19.6	33.0
4/5h*	117.3	49.3	150.0	58.3	184.7	62.2	24.5	45.0	22.9	42.5	27.6	52.8	24.6	42.7
18/19h*	149.6	63.6	205.7	79.7	251.5	84.0	27.2	48.7	20.8	36.7	19.9	39.0	20.5	35.2
24/29h*	109.0	46.0	162.0	80.8	247.0	85.7	27.2	47.3	23.2	38.5	22.1	42.0	22.0	37.5
44/48h*	64.0	27.3	157.2	64.0	234.0	83.2	16.6	29.0	30.4	53.0	29.2	54.0	31.9	54.2
72h	66.7	27.7	85.7	31.8	209.5	74.7	16.5	28.0	22.3	39.7	31.9	58.3	33.8	55.8
120h	59.7	25.2	71.5	29.2	70.0	24.3	21.2	36.8	21.1	36.0	18.5	33.8	35.5	60.0
168h	61.2	25.8	58.3	23.0	56.0	19.5								
240h							16.3	28.0	16.6	29.3	12.7	22.5	18.5	33.3
336h	66.3	27.8	57.2	21.8	77.2	26.7								
505h	55.7	24.2	59.0	21.5	66.5	22.3								
672h	58.3	24.3	61.7	22.8	71.0	24.2	17.1	27.2	21.2	35.3	13.0	22.2	17.0	27.8

*In CKD patients, Iron and TSAT were assessed at 5, 19, 29 and 44 hours, in healthy volunteers at 4, 18, 24 and 48 hours
