# Supplementary material for: First-in-human Phase I studies of PRS-080#22, a hepcidin antagonist, in healthy volunteers and patients with chronic kidney disease undergoing hemodialysis
Source: PLoS One. 2019 Mar 27;14(3):e0212023. doi: 10.1371/journal.pone.0212023 (PMC6436791; doi:10.1371/journal.pone.0212023)
Supplement: S6 Table — (PDF) [file pone.0212023.s013.pdf]

Time		CKD patients			Healthy volunteers			
	Placebo	2 mg	4 mg	8 mg	1.2 mg	4 mg	8 mg	16 mg
1/2 h*	-47.8	-27.2	43.8	19.2	-5.55	-2.83	1.08	-1.67
4/5h*	-12.2	-70,2	13.8	75.2	-2.95	0.08	1.05	1.95
18/19h*	-36.7	-20.4	46.5	51.2	0.17	2.33	1.87	12.93
24/29h*	-29.8	-43.0	1.3	55.0	1.30	1.45	0.70	17.23
44/48h*	-68.5	-55.3	4.3	29.7	-4.63	-9.53	-6.85	10.32
72h	-101.8	-67.7	-16.1	14.7	-1.37	-16.23	-7.67	0.47
120h	-194.7	-57.3	-15.1	-71.7	2.82	-12.75	-3.75	11.93
168h	-164.5	-93.5	-93.8	-148.8				
240h					-8.15	-15.03	5.43	-9.45
336h	-131.7	-79.9	-131.5	-120.5				
505h	-137.3	-129.7	-47.0	-146.2				
