# Supplementary material for: First-in-human Phase I studies of PRS-080#22, a hepcidin antagonist, in healthy volunteers and patients with chronic kidney disease undergoing hemodialysis
Source: PLoS One. 2019 Mar 27;14(3):e0212023. doi: 10.1371/journal.pone.0212023 (PMC6436791; doi:10.1371/journal.pone.0212023)
Supplement: S7 Table — (PDF) [file pone.0212023.s014.pdf]

Scheduled Study Time	Statistics	0.08 mg (N=6)	0.4 mg (N=6)	1.2 mg (N=6)	4 mg (N=6)	8 mg (N=6)	16 mg (N=6)	Placebo (N=12)
Day 1, pre-dose	n	6	6	6	6	6	6	12
	Mean	3.925	1.675	2.057	2.307	1.078	6.140	2.387
	SD	3.6515	0.9143	2.3484	2.1989	1.3822	12.0522	2.1719
	CV%	93.0	54.6	114.2	95.3	128.2	196.3	91.0
	Minimum	0.25	0.76	0.25	0.25	0.25	0.25	0.25
	Median	2.960	1.485	0.955	1.925	0.590	1.380	1.465
	Maximum	8.38	2.76	5.56	5.68	3.81	30.70	5.74
Day 1, 1h	n	6	6	6	6	6	6	12
	Mean	2.167	0.250	0.250	0.250	0.250	0.677	3.540
	SD	2.2006	0.0000	0.0000	0.0000	0.0000	1.0451	3.6645
	CV%	101.6	0.0	0.0	0.0	0.0	154.5	103.5
	Minimum	0.25	0.25	0.25	0.25	0.25	0.25	0.25
	Median	1.585	0.250	0.250	0.250	0.250	0.250	1.890
	Maximum	4.93	0.25	0.25	0.25	0.25	2.81	10.10
Day 1, 6h	n	6	6	6	6	6	6	12
	Mean	5.787	0.250	0.250	0.365	0.458	1.073	6.575
	SD	4.6589	0.0000	0.0000	0.2817	0.3262	1.2742	5.7550
	CV%	80.5	0.0	0.0	77.2	71.2	118.7	87.5
	Minimum	0.25	0.25	0.25	0.25	0.25	0.25	0.25
	Median	5.765	0.250	0.250	0.250	0.250	0.755	4.170
	Maximum	11.90	0.25	0.25	0.94	0.95	3.62	14.90
Day 2, 18h	n	6	6	6	6	6	6	12
	Mean	3.960	8.198	0.815	1.047	1.118	2.635	2.449
	SD	1.7639	7.7476	0.7213	0.9066	1.0164	2.3656	1.8513
	CV%	44.5	94.5	88.5	86.6	90.9	89.8	75.6
	Minimum	1.95	0.25	0.25	0.25	0.25	0.25	0.25
	Median	3.520	7.725	0.515	0.880	0.735	1.510	2.280
	Maximum	6.70	17.70	1.95	2.27	2.64	5.90	6.89
Day 3, 48h	n	6	6	6	6	6	6	12
	Mean	2.442	4.060	10.038	2.537	2.305	6.740	1.772
	SD	2.1846	1.9718	11.9604	2.5947	2.8913	12.5187	1.9017
	CV%	89.5	48.6	119.1	102.3	125.4	185.7	107.3
	Minimum	0.83	0.25	0.25	0.25	0.25	0.25	0.25
	Median	1.770	4.565	5.115	1.975	0.650	1.780	1.355
	Maximum	6.82	5.90	26.00	5.83	6.13	32.20	6.92

Scheduled Study Time	Statistics	0.08 mg (N=6)	0.4 mg (N=6)	1.2 mg (N=6)	4 mg (N=6)	8 mg (N=6)	16 mg (N=6)	Placebo (N=12)
Day 4, 72h	n	6	6	6	6	6	6	12
	Mean	2.178	3.870	7.840	19.790	28.247	48.177	1.831
	SD	1.6146	1.9401	8.3772	29.1053	66.0279	111.6203	2.6482
	CV%	74.1	50.1	106.9	147.1	233.8	231.7	144.6
	Minimum	1.04	0.25	0.25	0.25	0.25	0.54	0.25
	Median	1.645	4.285	6.620	2.220	1.465	3.340	0.875
	Maximum	5.43	6.05	19.60	63.70	163.00	276.00	9.78
Day 6, 120h	n	6	6	6	6	6	6	12
	Mean	2.017	2.668	5.808	8.157	28.930	33.532	3.057
	SD	1.5792	0.5524	5.2272	10.8022	41.4495	73.2353	4.7573
	CV%	78.3	20.7	90.0	132.4	143.3	218.4	155.6
	Minimum	0.66	1.88	0.25	0.25	0.25	2.16	0.25
	Median	1.765	2.830	5.705	2.220	6.540	3.620	0.765
	Maximum	4.97	3.38	13.20	22.40	98.40	183.00	13.00
Day 11, 240h	n	6	6	6	6	6	6	12
	Mean	1.133	3.665	2.443	5.218	21.045	38.527	0.572
	SD	0.8986	5.3707	2.2024	5.0327	23.1410	30.6146	0.4442
	CV%	79.3	146.5	90.1	96.4	110.0	79.5	77.7
	Minimum	0.25	0.66	0.25	0.25	0.25	2.42	0.25
	Median	0.985	1.415	2.325	4.675	17.985	42.500	0.250
	Maximum	2.87	14.50	5.92	13.30	62.30	83.50	1.35
Day 28	n	6	6	6	6	6	6	12
	Mean	1.162	2.140	2.012	2.183	6.593	4.760	2.531
	SD	0.7076	1.1040	3.8239	2.0151	10.8100	2.8559	4.6268
	CV%	60.9	51.6	190.1	92.3	164.0	60.0	182.8
	Minimum	0.25	0.94	0.25	0.25	0.25	0.25	0.25
	Median	0.960	2.050	0.250	1.650	3.335	5.020	0.515
	Maximum	2.09	3.32	9.77	4.86	28.40	7.43	14.10

Note that days were counted differently in the two studies, therefore hours are provided additionally, day 28 = 672 hours.
