# Supplementary material for: First-in-human Phase I studies of PRS-080#22, a hepcidin antagonist, in healthy volunteers and patients with chronic kidney disease undergoing hemodialysis
Source: PLoS One. 2019 Mar 27;14(3):e0212023. doi: 10.1371/journal.pone.0212023 (PMC6436791; doi:10.1371/journal.pone.0212023)
Supplement: S8 Table — (PDF) [file pone.0212023.s015.pdf]

		Hepcidin plasma level [nM]								
		N	missing	mean	SD	minimum	1st quartile	median	3rd quartile	maximum
**Visit**	**Treatment**									
Day 0 - pre-dose	**Treatment**									
	Placebo	6	0	15.880	9.010	6.20	9.380	13.450	22.400	30.40
	2 mg/kg	6	0	7.377	8.544	0.68	1.560	4.010	10.900	23.10
	4 mg/kg	6	0	14.367	17.291	2.06	2.410	7.215	20.700	46.60
	8 mg/kg	6	0	21.117	4.477	15.90	16.700	20.900	25.000	27.30
	**Total**	24	0	14.685	11.313	0.68	4.775	13.450	21.750	46.60
Day 0 - 1h	**Treatment**									
	Placebo	6	0	20.057	10.632	7.04	13.200	18.100	29.300	34.60
	2 mg/kg	6	0	3.045	6.235	0.00	0.000	0.490	1.590	15.70
	4 mg/kg	6	0	0.782	0.935	0.00	0.000	0.535	1.400	2.22
	8 mg/kg	6	0	1.137	0.672	0.00	0.970	1.125	1.690	1.91
	**Total**	24	0	6.255	10.017	0.00	0.000	1.325	10.120	34.60
Day 0 - 5h	**Treatment**									
	Placebo	6	0	27.000	14.598	10.40	18.900	23.350	34.400	51.60
	2 mg/kg	6	0	9.313	20.538	0.00	0.000	1.325	2.030	51.20
	4 mg/kg	6	0	1.563	2.275	0.00	0.000	0.685	2.190	5.82
	8 mg/kg	6	0	1.643	0.556	0.94	1.140	1.700	2.150	2.23
	**Total**	24	0	9.880	15.859	0.00	0.895	2.020	14.650	51.60
Day 1 - 19h	**Treatment**									
	Placebo	6	0	18.955	7.884	8.13	11.700	20.450	23.200	29.80
	2 mg/kg	5	0	50.084	31.568	8.82	38.100	40.900	74.000	88.60
	4 mg/kg	6	0	13.982	18.530	4.12	4.330	7.300	9.340	51.50
	8 mg/kg	6	0	12.078	3.063	8.48	9.890	11.300	15.700	15.80
	**Total**	23	0	22.631	22.388	4.12	8.820	12.000	29.800	88.60

		Hepcidin plasma level [nM]								
		N	missing	mean	SD	minimum	1st quartile	median	3rd quartile	maximum
Day 2 - 48h	**Treatment**									
	Placebo	6	0	20.303	12.708	7.32	11.800	15.700	31.200	40.10
	2 mg/kg	6	0	42.383	7.254	33.20	34.800	43.800	46.200	52.50
	4 mg/kg	5	1	75.042	45.185	9.31	53.800	81.100	109.000	122.00
	8 mg/kg	6	0	62.333	46.746	25.40	27.600	39.500	107.000	135.00
	**Total**	23	1	48.927	36.819	7.32	25.400	40.100	53.800	135.00
Day 3 - 72h	**Treatment**									
	Placebo	6	0	18.742	10.749	7.53	8.820	17.550	28.800	32.20
	2 mg/kg	6	0	48.017	22.190	22.60	38.600	44.400	49.500	88.60
	4 mg/kg	6	0	74.817	40.247	10.70	56.400	75.400	111.000	120.00
	8 mg/kg	6	0	123.783	52.823	73.80	89.500	111.700	137.000	219.00
	**Total**	24	0	66.340	51.469	7.53	26.100	52.950	90.950	219.00
Day 5 – 120h	**Treatment**									
	Placebo	6	0	24.503	14.676	7.94	9.980	23.850	38.600	42.80
	2 mg/kg	6	0	44.250	30.751	20.10	22.600	35.400	50.000	102.00
	4 mg/kg	6	0	69.067	32.577	34.30	48.700	58.250	91.900	123.00
	8 mg/kg	6	0	124.733	32.731	86.40	110.000	119.500	129.000	184.00
	**Total**	24	0	65.638	46.806	7.94	27.950	49.350	106.000	184.00
Day 14 – 240h	**Treatment**									
	Placebo	6	0	27.200	4.177	20.50	25.800	27.350	28.900	33.30
	2 mg/kg	6	0	28.300	13.167	12.70	15.700	27.350	41.500	45.20
	4 mg/kg	6	0	34.550	10.529	19.90	29.600	32.200	44.600	48.80
	8 mg/kg	6	0	79.883	23.065	36.20	71.900	91.100	94.100	94.90
	**Total**	24	0	42.483	25.999	12.70	26.550	32.200	47.000	94.90

		N	missing	mean	SD	minimum	1st quartile	median	3rd quartile	maximum
		**Hepcidin plasma level [nM]**								
Day 28 – 672 h	**Treatment**									
	Placebo	6	0	23.633	14.136	13.10	15.700	16.850	29.200	50.10
	2 mg/kg	6	0	26.522	20.578	8.53	9.400	18.550	48.400	55.70
	4 mg/kg	6	0	27.050	13.724	7.00	12.300	33.600	37.600	38.20
	8 mg/kg	6	0	47.517	11.502	32.00	35.400	50.300	56.200	60.90
	**Total**	24	0	31.180	17.315	7.00	14.800	32.500	48.650	60.90

Note that days were counted differently in the two studies, therefore hours are provided additionally.
