# Supplementary material for: First-in-human Phase I studies of PRS-080#22, a hepcidin antagonist, in healthy volunteers and patients with chronic kidney disease undergoing hemodialysis
Source: PLoS One. 2019 Mar 27;14(3):e0212023. doi: 10.1371/journal.pone.0212023 (PMC6436791; doi:10.1371/journal.pone.0212023)
Supplement: S9 Table — (PDF) [file pone.0212023.s016.pdf]

| Parameter | Time | CKD patients | | | Healthy volunteers | | | |
|---|---|---|---|---|---|---|---|---|
| | | 2 mg | 4 mg | 8 mg | 1.2 mg | 4 mg | 8 mg | 16 mg |
| Hb g/dl | 19/24h* | 0.62 | 1.32 | 1.23 | -0.05 | 0.23 | 0.10 | 0.12 |
| | 44/48h* | 0.02 | 0.42 | 0.57 | -0.17 | 0.1 | 0,13 | -0.12 |
| | 168h | -0.40 | 0.06 | 0.25 | | | | |
| | 240h | | | | -0.15 | -0.08 | 0.10 | -0.32 |
| | 672h | -0.58 | 0.30 | 0.48 | 0.10 | 0.12 | 0.22 | 0.07 |
| Ret 0/$_{00}$ | 19/24h* | -1.26 | -0.84 | 0.05 | 0.01 | 0.08 | 0.02 | -0.01 |
| | 44/48h* | -3.53 | -0.67 | -0.78 | 0.06 | 0.05 | 0.06 | 0.06 |
| | 168h | -1.40 | 0.96 | 2.90 | | | | |
| | 240h | | | | 0.15 | 0.18 | -0.06 | 0.44 |
| | 672h | -2.62 | -0.30 | -0 07 | 0.13 | -0.22 | -0.01 | 0.04 |
| RetHb pg | 19/24h* | -0.14 | -2.04 | 0.07 | -0.01 | 0.05 | 0.03 | -0.02 |
| | 44/48h* | -1.10 | -0.13 | 0.83 | 0.02 | -0.02 | 0.05 | 0.03 |
| | 168h | 0.5 | -0.16 | 0.83 | | | | |
| | 240h | | | | -0.03 | -0.05 | -0.03 | 0.05 |
| | 672h | -0.77 | 0.43 | 0.10 | -0.07 | -0.03 | -0.13 | -0.02 |

*In CKD patients, slow response parameters were assessed at 19 and 44 hours, in healthy volunteers at 24 and 48 hours
